# SCIPAC: quantitative estimation of cell-phenotype associations

**DOI:** 10.1186/s13059-024-03263-1

**Published:** 2024-05-13

**Authors:** Dailin Gan, Yini Zhu, Xin Lu, Jun Li

**Affiliations:** 1 Department of Applied and Computational Mathematics and Statistics, University of Notre Dame, Notre Dame, 46556 IN USA; 2 Department of Biological Sciences, Boler-Parseghian Center for Rare and Neglected Diseases, Harper Cancer Research Institute, Integrated Biomedical Sciences Graduate Program, University of Notre Dame, Notre Dame, 46556 IN USA; 3 Tumor Microenvironment and Metastasis Program, Indiana University Melvin and Bren Simon Comprehensive Cancer Center, Indianapolis, 46202 IN USA

**Keywords:** Phenotype association, Single cell, RNA sequencing, Cancer research

## Abstract

Numerous algorithms have been proposed to identify cell types in single-cell RNA sequencing data, yet a fundamental problem remains: determining associations between cells and phenotypes such as cancer. We develop SCIPAC, the first algorithm that quantitatively estimates the association between each cell in single-cell data and a phenotype. SCIPAC also provides a *p*-value for each association and applies to data with virtually any type of phenotype. We demonstrate SCIPAC’s accuracy in simulated data. On four real cancerous or noncancerous datasets, insights from SCIPAC help interpret the data and generate new hypotheses. SCIPAC requires minimum tuning and is computationally very fast.

**Supplementary Information:**

The online version contains supplementary material available at 10.1186/s13059-024-03263-1.

## Background

Single-cell RNA sequencing (scRNA-seq) technologies are revolutionizing biomedical research by providing comprehensive characterizations of diverse cell populations in heterogeneous tissues [1, 2]. Unlike bulk RNA sequencing (RNA-seq), which measures the average expression profile of the whole tissue, scRNA-seq gives the expression profiles of thousands of individual cells in the tissue [3–7]. Based on this rich data, cell types may be discovered/determined in an unsupervised (e.g., [8, 9]), semi-supervised (e.g., [10–13]), or supervised manner (e.g., [14–16]). Despite the fast development, there are still limitations with scRNA-seq technologies. Notably, the cost for each scRNA-seq experiment is still high; as a result, most scRNA-seq data are from a single or a few biological samples/tissues. Very few datasets consist of large numbers of samples with different phenotypes, e.g., cancer vs. normal. This places great difficulties in determining how a cell type contributes to a phenotype based on single-cell studies (especially if the cell type is discovered in a completely unsupervised manner or if people have limited knowledge of this cell type). For example, without having single-cell data from multiple cancer patients and multiple normal controls, it could be hard to computationally infer whether a cell type may promote or inhibit cancer development. However, such association can be critical for cancer research [17], disease diagnosis [18], cell-type targeted therapy development [19], etc.

Fortunately, this difficulty may be overcome by borrowing information from bulk RNA-seq data. Over the past decade, a considerable amount of bulk RNA-seq data from a large number of samples with different phenotypes have been accumulated and made available through databases like The Cancer Genome Atlas (TCGA) [20] and cBioPortal [21, 22]. Data in these databases often contain comprehensive patient phenotype information, such as cancer status, cancer stages, survival status and time, and tumor metastasis. Combining single-cell data from a single or a few individuals and bulk data from a relatively large number of individuals regarding a particular phenotype can be a cost-effective way to determine how a cell type contributes to the phenotype. A recent method Scissor [23] took an essential step in this direction. It uses single-cell and bulk RNA-seq data with phenotype information to classify the cells into three discrete categories: Scissor+, Scissor−, and null cells, corresponding to cells that are positively associated, negatively associated, and not associated with the phenotype.

Here, we present a method that takes another big step in this direction, which is called Single-Cell and bulk data-based Identifier for Phenotype Associated Cells or SCIPAC for short. SCIPAC enables quantitative estimation of the strength of association between each cell in a scRNA-seq data and a phenotype, with the help of bulk RNA-seq data with phenotype information. Moreover, SCIPAC also enables the estimation of the statistical significance of the association. That is, it gives a *p*-value for the association between each cell and the phenotype. Furthermore, SCIPAC enables the estimation of association between cells and an ordinal phenotype (e.g., different stages of cancer), which could be informative as people may not only be interested in the emergence/existence of cancer (cancer vs. healthy, a binary problem) but also in the progression of cancer (different stages of cancer, an ordinal problem).

To study the performance of SCIPAC, we first apply SCIPAC to simulated data under three schemes. SCIPAC shows high accuracy with low false positive rates. We further show the broad applicability of SCIPAC on real datasets across various diseases, including prostate cancer, breast cancer, lung cancer, and muscular dystrophy. The association inferred by SCIPAC is highly informative. In real datasets, some cell types have definite and well-studied functions, while others are less well-understood: their functions may be disease-dependent or tissue-dependent, and they may contain different sub-types with distinct functions. In the former case, SCIPAC’s results agree with current biological knowledge. In the latter case, SCIPAC’s discoveries inspire the generation of new hypotheses regarding the roles and functions of cells under different conditions.

## Results

### An overview of the SCIPAC algorithm

SCIPAC is a computational method that identifies cells in single-cell data that are associated with a given phenotype. This phenotype can be binary (e.g., cancer vs. normal), ordinal (e.g., cancer stage), continuous (e.g., quantitative traits), or survival (i.e., survival time and status). SCIPAC uses input data consisting of three parts: single-cell RNA-seq data that measures the expression of *p* genes in *m* cells, bulk RNA-seq data that measures the expression of the same set of *p* genes in *n* samples/tissues, and the statuses/values of the phenotype of the *n* bulk samples/tissues. The output of SCIPAC is the strength and the *p*-value of the association between each cell and the phenotype.

SCIPAC proposes the following definition of “association” between a cell and a phenotype: A group of cells that are likely to play a similar role in the phenotype (such as cells of a specific cell type or sub-type, cells in a particular state, cells in a cluster, cells with similar expression profiles, or cells with similar functions) is considered to be positively/negatively associated with a phenotype if an increase in their proportion within the tissue likely indicates an increased/decreased probability of the phenotype’s presence. SCIPAC assigns the same association to all cells within such a group. Taking cancer as the phenotype as an example, if increasing the proportion of a cell type indicates a higher chance of having cancer (binary), having a higher cancer stage (ordinal), or a higher hazard rate (survival), all cells in this cell type is positively associated with cancer.

The algorithm of SCIPAC follows the following four steps. First, the cells in the single-cell data are grouped into clusters according to their expression profiles. The Louvain algorithm from the Seurat package [24, 25] is used as the default clustering algorithm, but the user may choose any clustering algorithm they prefer. Or if information of the cell types or other groupings of cells is available a priori, it may be supplied to SCIPAC as the cell clusters, and this clustering step can be skipped. In the second step, a regression model is learned from bulk gene expression data with the phenotype. Depending on the type of the phenotype, this model can be logistic regression, ordinary linear regression, proportional odds model, or Cox proportional hazards model. To achieve a higher prediction power with less variance, by default, the elastic net (a blender of Lasso and ridge regression [26]) is used to fit the model. In the third step, SCIPAC computes the association strength $\Lambda$ between each cell cluster and the phenotype based on a mathematical formula that we derive. Finally, the *p*-values are computed. The association strength and its *p*-value between a cell cluster and the phenotype are given to all cells in the cluster.

SCIPAC requires minimum tuning. When the cell-type information is given in step 1, SCIPAC does not have any (hyper)parameter. Otherwise, the Louvain algorithm used in step 1 has a “resolution” parameter that controls the number of cell clusters: a larger resolution results in more clusters. SCIPAC inherits this parameter as its only parameter. Since SCIPAC gives the same association strength and *p*-value to cells from the same cluster, this parameter also determines the resolution of results provided by SCIPAC. Thus, we still call it “resolution” in SCIPAC. Because of its meaning, we recommend setting it so that the number of cell clusters given by the clustering algorithm is comparable to, or reasonably larger than, the number of cell types (or sub-types) in the data. We will see that the performance of SCIPAC is insensitive to this resolution parameter, and the default value 2.0 typically works well.

The details of the SCIPAC algorithm are given in the “Methods” section.

### Performance in simulated data

We assess the performance of SCIPAC in simulated data under three different schemes. The first scheme is simple and consists of only three cell types. The second scheme is more complicated and consists of seven cell types, which better imitates actual scRNA-seq data. In the third scheme, we simulate cells under different cell development stages to test the performance of SCIPAC under an ordinal phenotype. Details of the simulation are given in Additional file 1.

#### Simulation scheme I

**Fig. 1:**
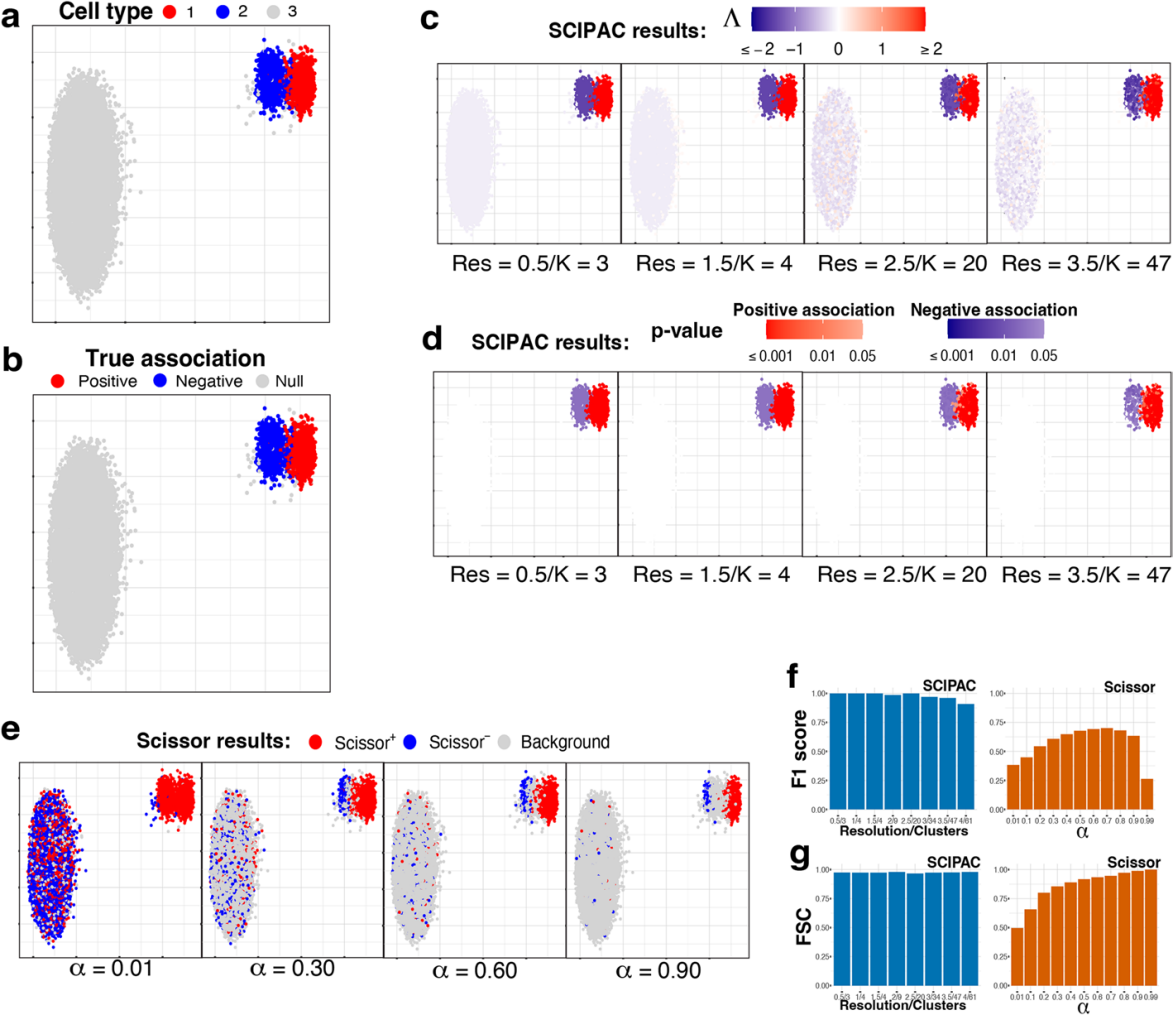
UMAP visualization and numeric measures of the simulated data under scheme I. All the plots in **a–e** are scatterplots of the two dimensional single-cell data given by UMAP. The *x* and *y* axes represent the two dimensions, and their scales are not shown as their specific values are not directly relevant. Points in the plots represents single cells, and they are colored differently in each subplot to reflect different information/results. **a** Cell types. **b** True associations. The association between cell types 1, 2, and 3 and the phenotype are positive, negative, and null, respectively. **c** Association strengths $\Lambda$ given by SCIPAC under different resolutions. Red/blue represents the sign of $\Lambda$, and the shade gives the absolute value of $\Lambda$. Every cell is colored red or blue since no $\Lambda$ is exactly zero. Below each subplot, Res stands for resolution, and K stands for the number of cell clusters given by this resolution. **d** *p*-values given by SCIPAC. Only cells with *p*-value $< 0.05$ are colored red (positive association) or blue (negative association); others are colored white. **e** Results given by Scissor under different $\alpha$ values. Red, blue, and light gray stands for Scissor+, Scissor−, and background (i.e., null) cells. **f** F1 scores and **g** FSC for SCIPAC and Scissor under different parameter values. For SCIPAC, each bar is the value under a resolution/number of clusters. For Scissor, each bar is the value under an $\alpha$

Under this scheme, the single-cell data consists of three cell types: one is positively associated with the phenotype, one is negatively associated, and the third is not associated (we call it “null”). Figure 1a gives the UMAP [27] plot of the three cell types, and Fig. 1b gives the true associations of these three cell types with the phenotype, with red, blue, and light gray denoting positive, negative, and null associations.

We apply SCIPAC to the simulated data. For the resolution parameter (see the “Methods” section), values 0.5, 1.0, and 1.5 give 3, 4, and 4 clusters, respectively, close to the actual number of cell types. They are good choices based on the guidance for choosing this parameter. To show how SCIPAC behaves under parameter misspecification, we also set the resolution up to 4.0, which gives a whopping 61 clusters. Figure 1c and d give the association strengths $\Lambda$ and the *p*-values given by four different resolutions (results under other resolutions are provided in Additional file 1: Fig. S1 and S2). In Fig. 1c, red and blue denote positive and negative associations, respectively, and the shade of the color represents the strength of the association, i.e., the absolute value of $\Lambda$. Every cell is colored blue or red, as none of $\Lambda$ is exactly zero. In Fig. 1d, red and blue denote positive and negative associations that are statistically significant (*p*-value $< 0.05$). Cells whose associations are not statistically significant (*p*-value $\ge 0.05$) are shown in white. To avoid confusion, it is worth repeating that cells that are colored in red/blue in Fig. 1c are shown in red/blue in Fig. 1d only if they are statistically significant; otherwise, they are colored white in Fig. 1d.

From Fig. 1c, d (as well as Additional file 1: Fig. S1 and S2), it is clear that the results of SCIPAC are highly consistent under different resolution values, including both the estimated association strengths and the *p*-values. It is also clear that SCIPAC is highly accurate: most truly associated cells are identified as significant, and most, if not all, truly null cells are identified as null.

As the first algorithm that quantitatively estimates the association strength and the first algorithm that gives the *p*-value of the association, SCIPAC does not have a real competitor. A previous algorithm, Scissor, is able to classify cells into three discrete categories according to their associations with the phenotype. So, we compare SCIPAC with Scissor in respect of the ability to differentiate positively associated, negatively associated, and null cells.

Running Scissor requires tuning a parameter called $\alpha$, which is a number between 0 and 1 that balances the amount of regularization for the smoothness and for the sparsity of the associations. The Scissor R package does not provide a default value for this $\alpha$ or a function to help select this value. The Scissor paper suggests the following criterion: “the number of Scissor-selected cells should not exceed a certain percentage of total cells (default 20%) in the single-cell data. In each experiment, a search on the above searching list is performed from the smallest to the largest until a value of $\alpha$ meets the above criteria.” In practice, we have found that this criterion does not often work properly, as the truly associated cells may not compose 20% of all cells in actual data. Therefore, instead of setting $\alpha$ to any particular value, we set $\alpha$ values that span the whole range of $\alpha$ to see the best possible performance of Scissor.

The performance of Scissor in our simulation data under four different $\alpha$ values are shown in Fig. 1e, and results under more $\alpha$ values are shown in Additional file 1: Fig. S3. In the figures, red, blue, and light gray denote Scissor+, Scissor−, and null (called “background” in Scissor) cells, respectively. The results of Scissor have several characteristics different from SCIPAC. First, Scissor does not give the strength or statistical significance of the association, and thus the colors of the cells in the figures do not have different shades. Second, different $\alpha$ values give very different results. Greater $\alpha$ values generally give fewer Scissor+ and Scissor− cells, but there are additional complexities. One complexity is that the Scissor+ (or Scissor−) cells under a greater $\alpha$ value are not a strict subset of Scissor+ (or Scissor−) cells under a smaller $\alpha$ value. For example, the number of truly negatively associated cells detected as Scissor− increases when $\alpha$ increases from 0.01 to 0.30. Another complexity is that the direction of the association may flip as $\alpha$ increases. For example, most cells of cell type 2 are identified as Scissor+ under $\alpha =0.01$, but many of them are identified as Scissor− under larger $\alpha$ values. Third, Scissor does not achieve high power and low false-positive rate at the same time under any $\alpha$. No matter what the $\alpha$ value is, there is only a small proportion of cells from cell type 2 that are correctly identified as negatively associated, and there is always a non-negligible proportion of null cells (i.e., cells from cell type 3) that are incorrectly identified as positively or negatively associated. Fourth, Scissor+ and Scissor− cells can be close to each other in the figure, even under a large $\alpha$ value. This means that cells with nearly identical expression profiles are detected to be associated with the phenotype in opposite directions, which can place difficulties in interpreting the results.

SCIPAC overcomes the difficulties of Scissor and gives results that are more informative (quantitative strengths with *p*-values), more accurate (both high power and low false-positive rate), less sensitive to the tuning parameter, and easier to interpret (cells with similar expression typically have similar associations to the phenotype).

SCIPAC’s higher accuracy in differentiating positively associated, negatively associated, and null cells than Scissors can also be measured numerically using the F1 score and the fraction of sign correctness (FSC). F1, which is the harmonic mean of precision and recall, is a commonly used measure of calling accuracy. Note that precision and recall are only defined for two-class problems, which try to classify desired signals/discoveries (so-called “positives”) against noises/trivial results (so-called “negatives”). Our case, on the other hand, is a three-class problem: positive association, negative association, and null. To compute F1, we combine the positive and negative associations and treat them as “positives,” and treat null as “negatives.” This F1 score ignores the direction of the association; thus, it alone is not enough to describe the performance of an association-detection algorithm. For example, an algorithm may have a perfect F1 score even if it incorrectly calls all negative associations positive. To measure an algorithm’s ability to determine the direction of the association, we propose a statistic called FSC, defined as the fraction of true discoveries that also have the correct direction of the association. The F1 score and FSC are numbers between 0 and 1, and higher values are preferred. A mathematical definition of these two measures is given in Additional file 1.

Figure 1f, g show the F1 score and FSC of SCIPAC and Scissor under different values of tuning parameters. The F1 score of Scissor is between 0.2 and 0.7 under different $\alpha$’s. The FSC of Scissor increases from around 0.5 to nearly 1 as $\alpha$ increases, but Scissor does not achieve high F1 and FSC scores at the same time under any $\alpha$. On the other hand, the F1 score of SCIPAC is close to perfection when the resolution parameter is properly set, and it is still above 0.90 even if the resolution parameter is set too large. The FSC of SCIPAC is always above 0.96 under different resolutions. That is, SCIPAC achieves high F1 and FSC scores simultaneously under a wide range of resolutions, representing a much higher accuracy than Scissor.

#### Simulation scheme II

**Fig. 2:**
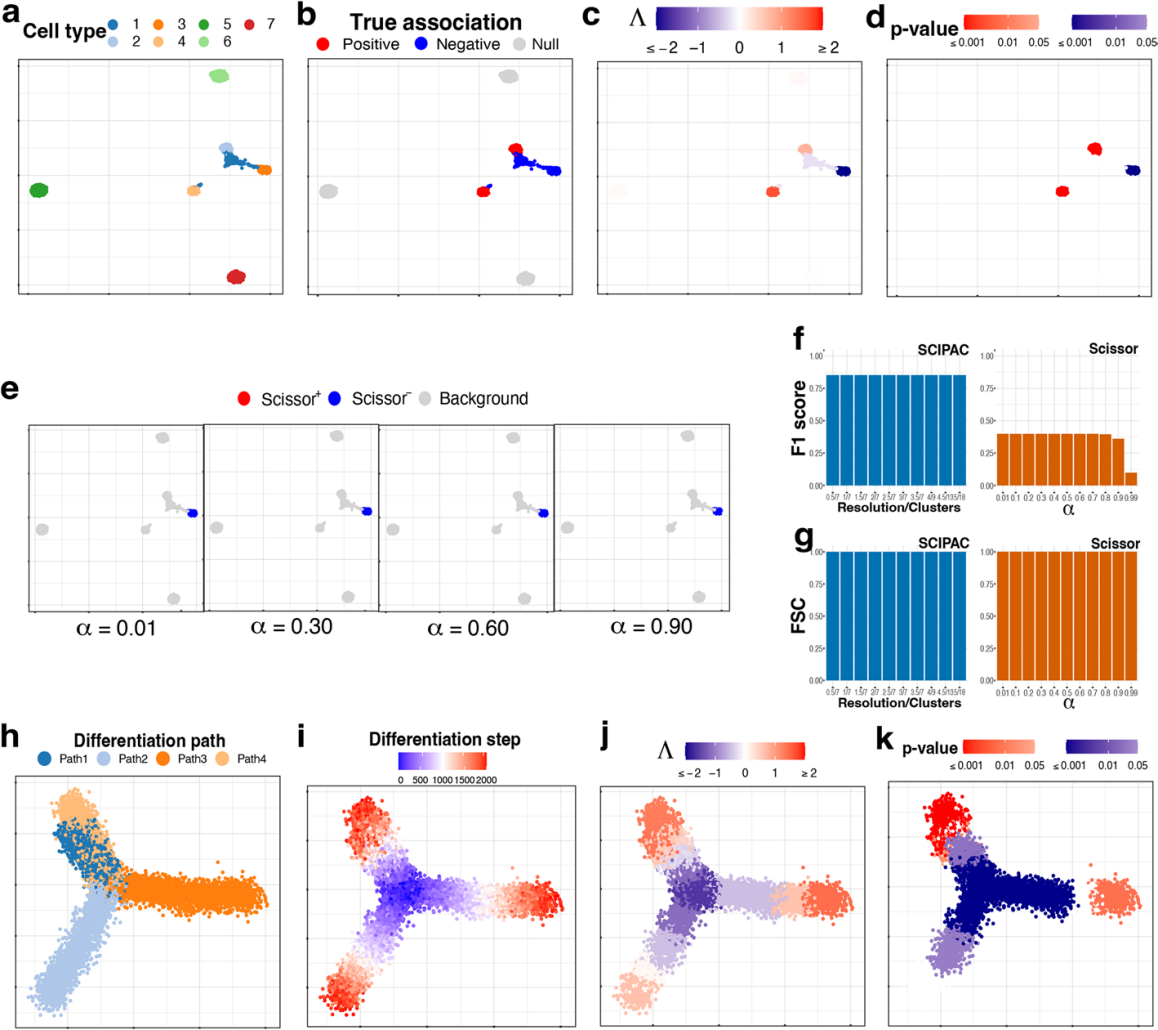
UMAP visualization of the simulated data under **a–g** scheme II and **h–k** scheme III. **a** Cell types. **b** True associations. **c**, **d** Association strengths $\Lambda$ and *p*-values given by SCIPAC under the default resolution. **e** Results given by Scissor under different $\alpha$ values. **f** F1 scores and **g** FSC for SCIPAC and Scissor under different parameter values. **h** Cell differentiation paths. The four paths have the same starting location, which is in the center, but different ending locations. This can be considered as a progenitor cell type differentiating into four specialized cell types. **i** Cell differentiation steps. These steps are used to create four stages, each containing 500 steps. Thus, this plot of differentiation steps can also be viewed as the plot of true association strengths. **j**, **k** Association strengths $\Lambda$ and *p*-values given by SCIPAC under the default resolution

This more complicated simulation scheme has seven cell types, which are shown in Fig. 2a. As shown in Fig. 2b, cell types 1 and 3 are negatively associated (colored blue), 2 and 4 are positively associated (colored red), and 5, 6, and 7 are not associated (colored light gray).

The association strengths and *p*-values given by SCIPAC under the default resolution are illustrated in Fig. 2c, d, respectively. Results under several other resolutions are given in Additional file 1: Fig. S4 and S5. Again, we find that SCIPAC gives highly consistent results under different resolutions. SCIPAC successfully identifies three out of the four truly associated cell types. For the other truly associated cell type, cell type 1, SCIPAC correctly recognizes its association with the phenotype as negative, although the *p*-values are not significant enough. The F1 score is 0.85, and the FSC is greater than 0.99, as shown in Fig. 2f, g.

The results of Scissor under four different $\alpha$ values are given in Fig. 2e. (More shown in Additional file 1: Fig. S6.) Under this highly challenging simulation scheme, Scissor can only identify one out of four truly associated cell types. Its F1 score is below 0.4.

#### Simulation scheme III

This simulation scheme is to assess the performance of SCIPAC for ordinal phenotypes. We simulate cells along four cell-differentiation paths with the same starting location but different ending locations, as shown in Fig. 2h. These cells can be considered as a progenitor cell population differentiating into four specialized cell types. In Fig. 2i, the “step” reflects their position in the differentiation path, with step 0 meaning the start and step 2000 meaning the end of the differentiation. Then, the “stage” is generated according to the step: cells in steps 0$\sim$ 500, 501$\sim$1000, 1001$\sim$1500, and 1501$\sim$2000 are assigned to stages I, II, III, and IV, respectively. This stage is treated as the ordinal phenotype. Under this simulation scheme, Fig. 2i also gives the actual associations, and all cells are associated with the phenotype.

The results of SCIPAC under the default resolution are shown in Fig. 2j, k. Clearly, the associations SCIPAC identifies are highly consistent with the truth. Particularly, it successfully identifies the cells in the center as early-stage cells and most cells at the end of branches as last-stage cells. The results of SCIPAC under other resolutions are given in Additional file 1: Fig. S7 and S8, which are highly consistent. Scissor does not work with ordinal phenotypes; thus, no results are reported here.

### Performance in real data

We consider four real datasets: a prostate cancer dataset, a breast cancer dataset, a lung cancer dataset, and a muscular dystrophy dataset. The bulk RNA-seq data of the three cancer datasets are obtained from the TCGA database, and that of the muscular dystrophy dataset is obtained from a published paper [28]. A detailed description of these datasets is given in Additional file 1. We will use these datasets to assess the performance of SCIPAC on different types of phenotypes. The cell type information (i.e., which cell belongs to which cell type) is available for the first three datasets, but we ignore this information so that we can make a fair comparison with Scissor, which cannot utilize this information.

#### Prostate cancer data with a binary phenotype

**Fig. 3:**
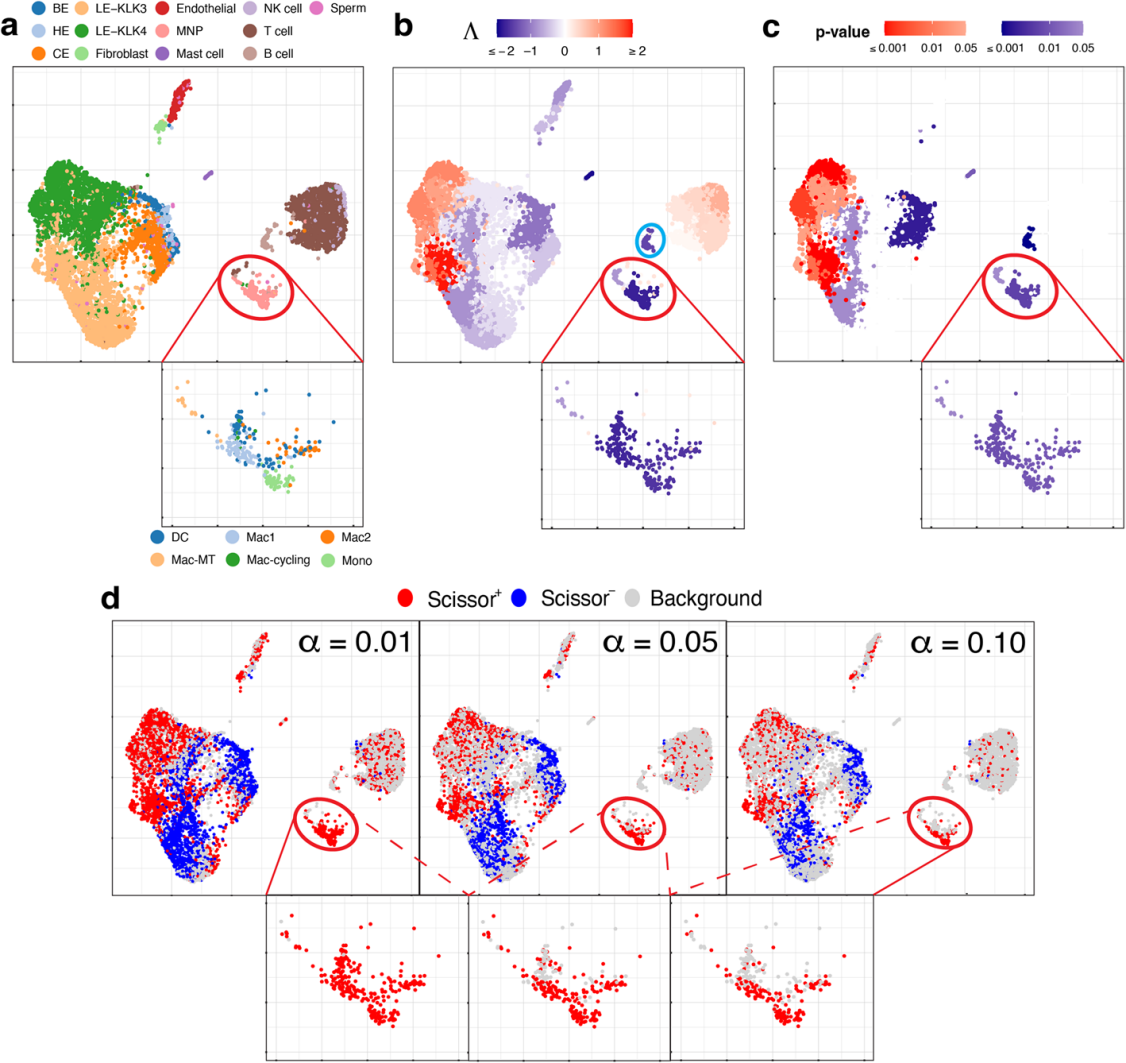
UMAP visualization of the prostate cancer data, with a zoom-in view for the red-circled region (cell type MNP). **a** True cell types. BE, HE, and CE stand for basal, hillock, club epithelial cells, LE-KLK3 and LE-KLK4 stand for luminal epithelial cells with high levels of kallikrein related peptidase 3 and 4, and MNP stands for mononuclear phagocytes. In the zoom-in view, the sub-types of MNP cells are given. **b** Association strengths $\Lambda$ given by SCIPAC under the default resolution. The cyan-circled cells are B cells, which are estimated by SCIPAC as negatively associated with cancer but estimated by Scissor as Scissor+ or null. **c** *p*-values given by SCIPAC. The MNP cell type, which is red-circled in the plot, is estimated by SCIPAC to be strongly negatively associated with cancer but estimated by Scissor to be positively associated with cancer. **d** Results given by Scissor under different $\alpha$ values

We use the single-cell expression of 8,700 cells from prostate-cancer tumors sequenced by [29]. The cell types of these cells are known and given in Fig. 3a. The bulk data comprises 550 TCGA-PRAD (prostate adenocarcinoma) samples with phenotype (cancer vs. normal) information. Here the phenotype is cancer, and it is binary: present or absent.

Results from SCIPAC with the default resolution are shown in Fig. 3b, c (results with other resolutions, given in Additional file 1: Fig. S9 and S10, are highly consistent with results here.) Compared with results from Scissor, shown in Fig. 3d, results from SCIPAC again show three advantages. First, results from SCIPAC are richer and more comprehensive. SCIPAC gives estimated associations and the corresponding *p*-values, and the estimated associations are quantitative (shown in Fig. 3b as different shades to the red or blue color) instead of discrete (shown in Fig. 3d as a uniform shade to the red, blue, or light gray color). Second, SCIPAC’s results can be easier to interpret as the red and blue colors are more block-wise instead of scattered. Third, unlike Scissor, which produces multiple sets of results varying based on the parameter $\alpha$—a parameter without a default value or tuning guidance—typically, a single set of results from SCIPAC under its default settings suffices.

Comparing the results from our SCIPAC method with those from Scissor is non-trivial, as the latter’s outcomes are scattered and include multiple sets. We propose the following solutions to summarize the inferred association of a known cell type with the phenotype using a specific method (Scissor under a specific $\alpha$ value, or SCIPAC with the default setting). We first calculate the proportion of cells in this cell type identified as Scissor+ (by Scissor at a specific $\alpha$ value) or as significantly positively associated (by SCIPAC), denoted by $p_{+}$. We also calculate the proportion of all cells, encompassing any cell type, which are identified as Scissor+ or significantly positively associated, serving as the average background strength, denoted by $p_{a}$. Then, we compute the log odds ratio for this cell type to be positively associated with the phenotype compared to the background, represented as:

$$\begin{aligned} \rho _{+} = \log \frac{p_+ / (1 - p_+)}{p_a / (1 - p_a)}. \end{aligned}$$

Similarly, the log odds ratio for the cell type to be negatively associated with the phenotype, $\rho _-$, is computed in a parallel manner.

For SCIPAC, a cell type is summarized as positively associated with the phenotype if $\rho _+ \ge 1$ and $\rho _- < 1$ and negatively associated if $\rho _- \ge 1$ and $\rho _+ < 1$. If neither condition is met, the association is inconclusive. For Scissor, we apply it under six different $\alpha$ values: 0.01, 0.05, 0.10, 0.15, 0.20, and 0.25. A cell type is summarized as positively associated with the phenotype if $\rho _+ \ge 1$ and $\rho _- < 1$ in at least four of these $\alpha$ values and negatively associated if $\rho _- \ge 1$ and $\rho _+ < 1$ in at least four $\alpha$ values. If these criteria are not met, the association is deemed inconclusive. The above computation of log odds ratios and the determination of associations are performed only on cell types that each compose at least 1% of the cell population, to ensure adequate power.

For the prostate cancer data, the log odds ratios for each cell type using each method are presented in Tables S1 and S2. The final associations determined for each cell type are summarized in Table S3. In the last column of this table, we also indicate whether the conclusions drawn from SCIPAC and Scissor are consistent or not.

We find that SCIPAC’s results agree with Scissor on most cell types. However, there are three exceptions: mononuclear phagocytes (MNPs), B cells, and LE-KLK4.

MNPs are red-circled and zoomed in in each sub-figure of Fig. 3. Most cells in this cell type are colored red in Fig. 3d but colored dark blue in Fig. 3b. In other words, while Scissor determines that this cell type is Scissor+, SCIPAC makes the opposite inference. Moreover, SCIPAC is confident about its judgment by giving small *p*-values, as shown in Fig. 3c. To see which inference is closer to the biological fact is not easy, as biologically MNPs contain a number of sub-types that each have different functions [30, 31]. Fortunately, this cell population has been studied in detail in the original paper that generated this dataset [29], and the sub-type information of each cell is provided there: this MNP population contains six sub-types, which are dendritic cells (DC), M1 macrophages (Mac1), metallothionein-expressing macrophages (Mac-MT), M2 macrophages (Mac2), proliferating macrophages (Mac-cycling), and monocytes (Mono), as shown in the zoom-in view of Fig. 3a. Among these six sub-types, DC, Mac1, and Mac-MT are believed to inhibit cancer development and can serve as targets in cancer immunotherapy [29]; they compose more than 60% of all MNP cells in this dataset. SCIPAC makes the correct inference on this majority of MNP cells. Another cell type, Mac2, is reported to promote tumor development [32], but it only composes less than $15\%$ of the MNPs. How the other two cell types, Mac-cycling and Mono, are associated with cancer is less studied. Overall, the results given by SCIPAC are more consistent with the current biological knowledge.

B cells are cyan-circled in Fig. 3b. B cells are generally believed to have anti-tumor activity by producing tumor-reactive antibodies and forming tertiary lymphoid structures [29, 33]. This means that B cells are likely to be negatively associated with cancer. SCIPAC successfully identifies this negative association, while Scissor fails.

LE-KLK4, a subtype of cancer cells, is thought to be positively associated with the tumor phenotype [29]. SCIPAC successfully identified this positive association, in contrast to Scissor, which failed to do so (in the figure, a proportion of LE-KLK4 cells are identified as Scissor+, especially under the smallest $\alpha$ value; however, this proportion is not significantly higher than the background Scissor+ level under the majority of $\alpha$ values).

In summary, across all three cell types, the results from SCIPAC appear to be more consistent with current biological knowledge. For more discussions regarding this dataset, refer to Additional file 1.

#### Breast cancer data with an ordinal phenotype

**Fig. 4:**
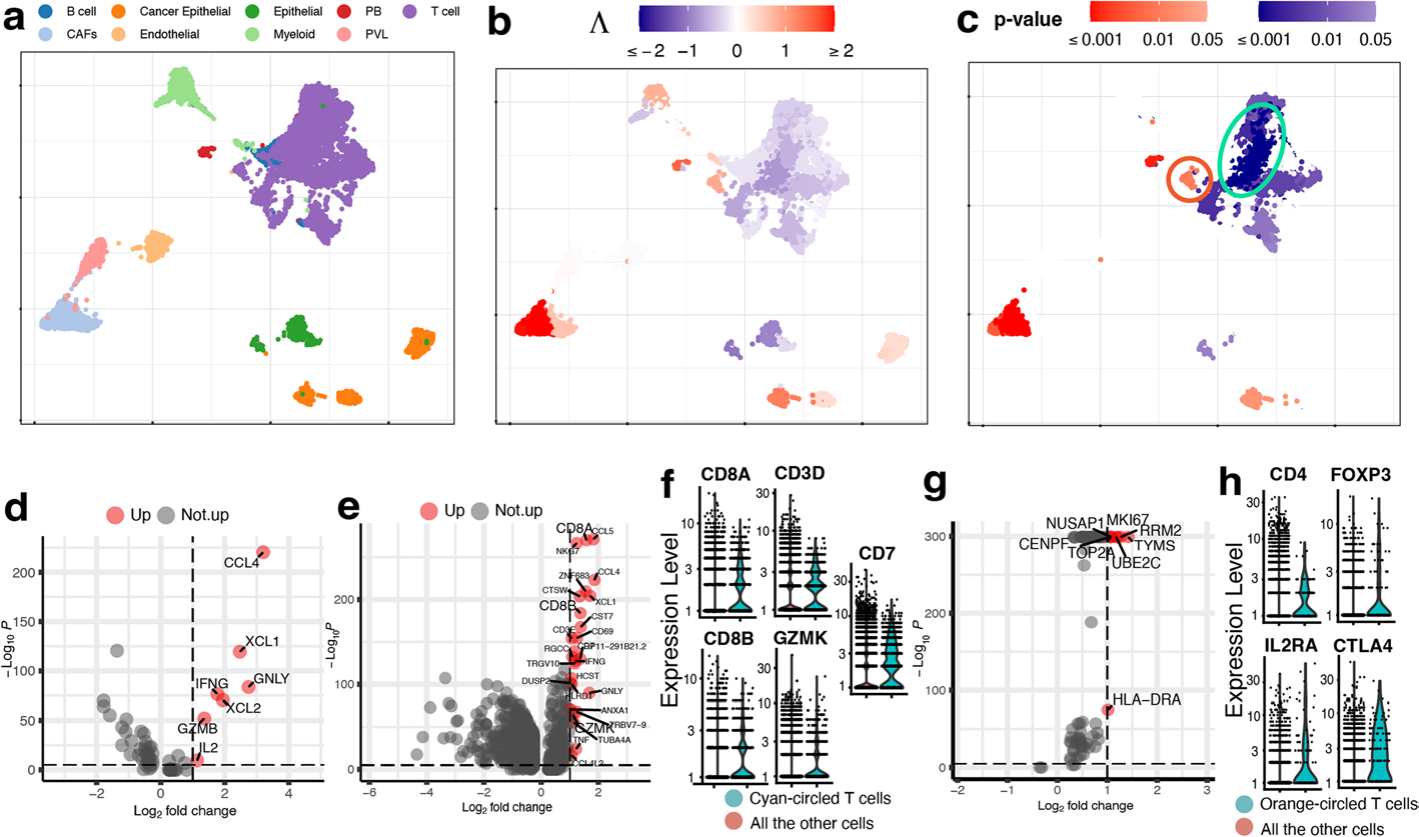
UMAP visualization of the breast cancer data. **a** True cell types. CAFs stand for cancer-associated fibroblasts, PB stands for plasmablasts and PVL stands for perivascular-like cells. **b**, **c** Association strengths $\Lambda$ and *p*-values given by SCIPAC under the default resolution. Cyan-circled are a group of T cells that are estimated by SCIPAC to be most significantly associated with the cancer stage in the negative direction, and orange-circled are a group of T cells that are estimated by SCIPAC to be significantly positively associated with the cancer stage. **d** DE analysis of the cyan-circled T cells vs. all the other T cells. **e** DE analysis of the cyan-circled T cells vs. all the other cells. **f** Expression of CD8+ T cell marker genes in the cyan-circled cells and all the other cells. **g** DE analysis of the orange-circled T cells vs. all the other cells. **h** Expression of regulatory T cell marker genes in the orange-circled cells and all the other cells

The scRNA-seq data for breast cancer are from [34], and we use the 19,311 cells from the five HER2+ tumor tissues. The true cell types are shown in Fig. 4a. The bulk data include 1215 TCGA-BRCA samples with information on the cancer stage (I, II, III, or IV), which is treated as an ordinal phenotype.

Association strengths and *p*-values given by SCIPAC under the default resolution are shown in Fig. 4b, c. Results under other resolutions are given in Additional file 1: Fig. S11 and S12, and again they are highly consistent with results under the default resolution. We do not present the results from Scissor, as Scissor does not take ordinal phenotypes.

In the SCIPAC results, cells that are most strongly and statistically significantly associated with the phenotype in the positive direction are the cancer-associated fibroblasts (CAFs). This finding agrees with the literature: CAFs contribute to therapy resistance and metastasis of cancer cells via the production of secreted factors and direct interaction with cancer cells [35], and they are also active players in breast cancer initiation and progression [36–39]. Another large group of cells identified as positively associated with the phenotype is the cancer epithelial cells. They are malignant cells in breast cancer tissues and are thus expected to be associated with severe cancer stages.

Of the cells identified as negatively associated with severe cancer stages, a large portion of T cells is the most noticeable. Biologically, T cells contain many sub-types, including CD4+, CD8+, regulatory T cells, and more, and their functions are diverse in the tumor microenvironment [40]. To explore SCIPAC’s discoveries, we compare T cells that are identified as most statistically significant, with *p*-values $< 10^{-6}$ and circled in Fig. 4d, with the other T cells. Differential expression (DE) analysis (details about DE analysis and other analyses are given in Additional file 1) identifies seven genes upregulated in these most significant T cells. Of these seven genes, at least five are supported by the literature: CCL4, XCL1, IFNG, and GZMB are associated with CD8+ T cell infiltration; they have been shown to have anti-tumor functions and are involved in cancer immunotherapy [41–43]. Also, IL2 has been shown to serve an important role in combination therapies for autoimmunity and cancer [44]. We also perform an enrichment analysis [45], in which a pathway called Myc stands out with a $\textit{p}\text{-value}<10^{-7}$, much smaller than all other pathways. Myc is downregulated in the T cells that are identified as most negatively associated with cancer stage progress. This agrees with current biological knowledge about this pathway: Myc is known to contribute to malignant cell transformation and tumor metastasis [46–48].

On the above, we have compared T cells that are most significantly associated with cancer stages in the negative direction with the other T cells using DE and pathway analysis, and the results could suggest that these cells are tumor-infiltrated CD8+ T cells with tumor-inhibition functions. To check this hypothesis, we perform DE analysis of these cells against all other cells (i.e., the other T cells and all the other cell types). The DE genes are shown in Fig. 4e. It can be noted that CD8+ T cell marker genes such as CD8A, CD8B, and GZMK are upregulated. We further obtain CD8+ T cell marker genes from *CellMarker* [49] and check their expression, as illustrated in Fig. 4f. Marker genes CD8A, CD8B, CD3D, GZMK, and CD7 show significantly higher expression in these T cells. This again supports our hypothesis that these cells are tumor-infiltrated CD8+ T cells that have anti-tumor functions.

Interestingly, not all T cells are identified as negatively associated with severe cancer stages; a group of T cells is identified as positively associated, as circled in Fig. 4c. To explore the function of this group of T cells, we perform DE analysis of these T cells against the other T cells. The DE genes are shown in Fig. 4g. Based on the literature, six out of eight over-expressed genes are associated with cancer development. The high expression of NUSAP1 gene is associated with poor patient overall survival, and this gene also serves as a prognostic factor in breast cancer [50–52]. Gene MKI67 has been treated as a candidate prognostic prediction for cancer proliferation [53, 54]. The over-expression of RRM2 has been linked to higher proliferation and invasiveness of malignant cells [55, 56], and the upregulation of RRM2 in breast cancer suggests it to be a possible prognostic indicator [57–62]. The high expression of UBE2C gene always occurs in cancers with a high degree of malignancy, low differentiation, and high metastatic tendency [63]. For gene TOP2A, it has been proposed that the HER2 amplification in HER2 breast cancers may be a direct result of the frequent co-amplification of TOP2A [64–66], and there is a high correlation between the high expressions of TOP2A and the oncogene HER2 [67, 68]. Gene CENPF is a cell cycle-associated gene, and it has been identified as a marker of cell proliferation in breast cancers [69]. The over-expression of these genes strongly supports the correctness of the association identified by SCIPAC. To further validate this positive association, we perform DE analysis of these cells against all the other cells. We find that the top marker genes obtained from *CellMarker* [49] for the regulatory T cells, which are known to be immunosuppressive and promote cancer progression [70], are over-expressed with statistical significance, as shown in Fig. 4h. This finding again provides strong evidence that the positive association identified by SCIPAC for this group of T cells is correct.

#### Lung cancer data with survival information

**Fig. 5:**
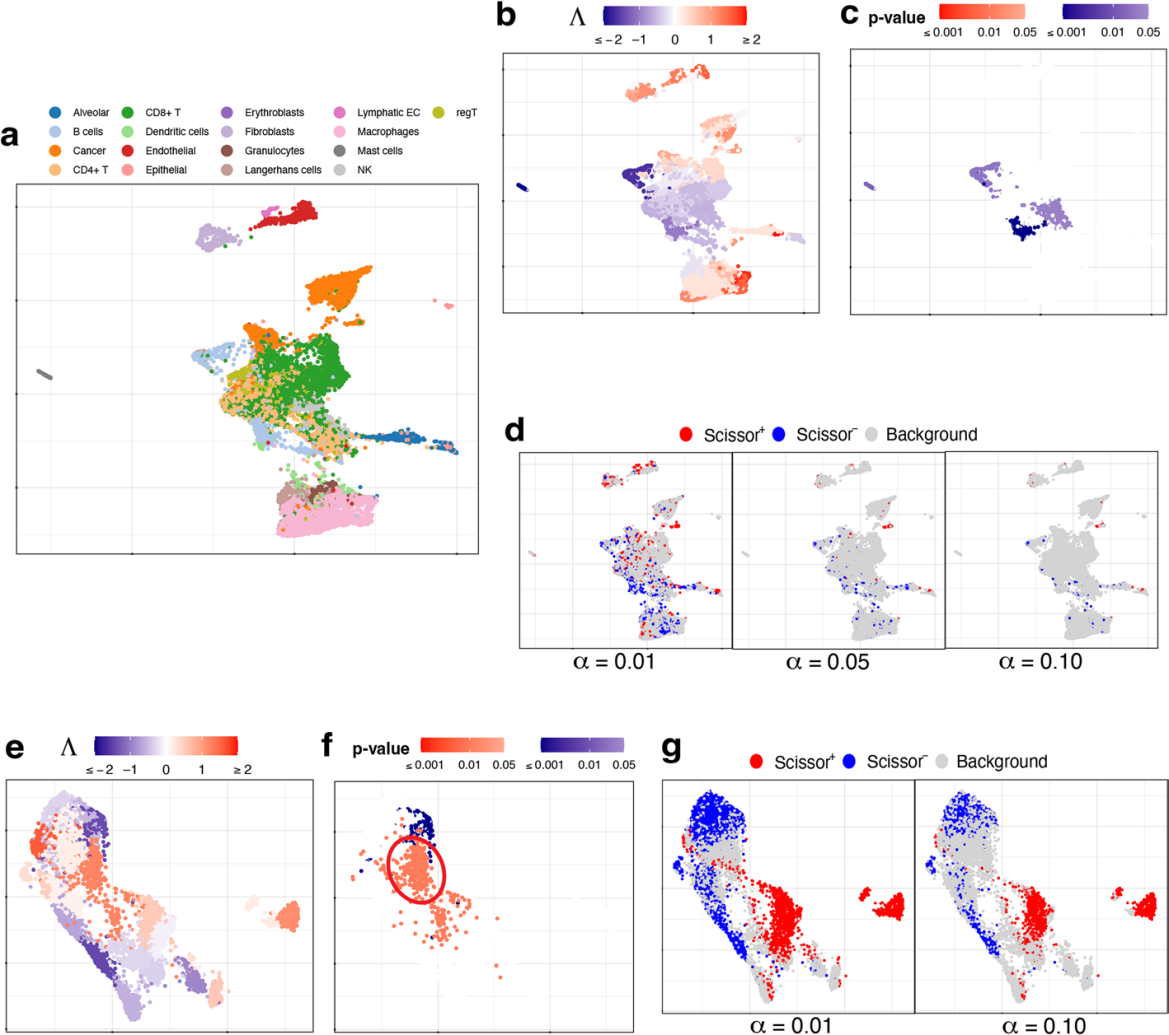
UMAP visualization of **a–d** the lung cancer data and **e–g** the muscular dystrophy data. **a** True cell types. **b**, **c** Association strengths $\Lambda$ and *p*-values given by SCIPAC under the default resolution. **d** Results given by Scissor under different $\alpha$ values. **e**, **f** Association strengths $\Lambda$ and *p*-values given by SCIPAC under the default resolution. Circled are a group of cells that are identified by SCIPAC as significantly positively associated with the disease but identified by Scissor as null. **g** Results given by Scissor under different $\alpha$ values

The scRNA-seq data for lung cancer are from [71], and we use two lung adenocarcinoma (LUAD) patients’ data with 29,888 cells. The true cell types are shown in Fig. 5a. The bulk data consist of 576 TCGA-LUAD samples with survival status and time.

Association strengths and *p*-values given by SCIPAC are given in Fig. 5b, c (results under other resolutions are given in Additional file 1: Fig. S13 and S14). In Fig. 5c, most cells with statistically significant associations are CD4+ T cells or B cells. These associations are negative, meaning that the abundance of these cells is associated with a reduced death rate, i.e., longer survival time. This agrees with the literature: CD4+ T cells primarily mediate anti-tumor immunity and are associated with favorable prognosis in lung cancer patients [72–74]; B cells also show anti-tumor functions in all stages of human lung cancer development and play an essential role in anti-tumor responses [75, 76].

The results by Scissor under different $\alpha$ values are shown in Fig. 5d. The highly scattered Scissor+ and Scissor− cells make identifying and interpreting meaningful phenotype-associated cell groups difficult.

#### Muscular dystrophy data with a binary phenotype

This dataset contains cells from four facioscapulohumeral muscular dystrophy (FSHD) samples and two control samples [77]. We pool all the 7047 cells from these six samples together. The true cell types of these cells are unknown. The bulk data consists of 27 FSHD patients and eight controls from [28]. Here the phenotype is FSHD, and it is binary: present or absent.

The results of SCIPAC with the default resolution are given in Fig. 5e, f. Results under other resolutions are highly similar (shown in Additional file 1: Fig. S15 and S16). For comparison, results given by Scissor under different $\alpha$ values are presented in Fig. 5g. The agreements between the results of SCIPAC and Scissor are clear. For example, both methods identify cells located at the top and lower left part of UMAP plots to be negatively associated with FSHD, and cells located at the center and right parts of UMAP plots to be positively associated. However, the discrepancies in their results are also evident. The most pronounced one is a large group of cells (circled in Fig. 5f) that are identified by SCIPAC as significantly positively associated but are completely ignored by Scissor. Checking into this group of cells, we find that over 90% (424 out of 469) come from the FSHD patients, and less than 10% come from the control samples. However, cells from FSHD patients only compose 73% (5133) of all the 7047 cells. This statistically significant (*p*-value $<10^{-15}$, Fisher’s exact test) over-representation (odds ratio = 3.51) suggests that the positive association identified SCIPAC is likely to be correct.

## Discussion

SCIPAC is computationally highly efficient. On an 8-core machine with 2.50 GHz CPU and 16 GB RAM, SCIPAC takes 7, 24, and 2 s to finish all the computation and give the estimated association strengths and *p*-values on the prostate cancer, lung cancer, and muscular dystrophy datasets, respectively. As a reference, Scissor takes 314, 539, and 171 seconds, respectively.

SCIPAC works with various phenotype types, including binary, continuous, survival, and ordinal. It can easily accommodate other types by using a proper regression model with a systematic component in the form of Eq. 3 (see the “Methods” section). For example, a Poisson or negative binomial log-linear model can be used if the phenotype is a count (i.e., non-negative integer).

In SCIPAC’s definition of association, a cell type is associated with the phenotype if increasing the proportion of this cell type leads to a change of probability of the phenotype occurring. The strength of association represents the extent of the increase or decrease in this probability. In the case of binary-response, this change is measured by the log odds ratio. For example, if the association strength of cell type A is twice that of cell type B, increasing cell type A by a certain proportion leads to twice the amount of change in the log odds ratio of having the phenotype compared to increasing cell type B by the same proportion. The association strength under other types of phenotypes can be interpreted similarly, with the major difference lying in the measure of change in probability. For quantitative, ordinal, and survival outcomes, the difference in the quantitative outcome, log odds ratio of the right-tail probability, and log hazard ratio respectively are used. Despite the differences in the exact form of the association strength under different types of phenotypes, the underlying concept remains the same: a larger (absolute value of) association strength indicates that the same increase/decrease in a cell type leads to a larger change in the occurrence of the phenotype.

As SCIPAC utilizes both bulk RNA-seq data with phenotype and single-cell RNA-seq data, the estimated associations for the cells are influenced by the choice of the bulk data. Although different bulk data can yield varying estimations of the association for the same single cells, the estimated associations appear to be reasonably robust even when minor changes are made to the bulk data. See Additional file 1 for further discussions.

When using the Louvain algorithm in the Seurat package to cluster cells, SCIPAC’s default resolution is 2.0, larger than the default setting of Seurat. This allows for the identification of potential subtypes within the major cell type and enables the estimation of individual association strengths. Consequently, a more detailed and comprehensive description of the association between single cells and the phenotype can be obtained by SCIPAC.

When applying SCIPAC to real datasets, we made a deliberate choice to disregard the cell annotation provided by the original publications and instead relied on the inferred cell clusters produced by the Louvain algorithm. We made this decision for several reasons. Firstly, we aimed to ensure a fair comparison with Scissor, as it does not utilize cell-type annotations. Secondly, the original annotation might not be sufficiently comprehensive or detailed. Presumed cell types could potentially encompass multiple subtypes, each of which may exhibit distinct associations with the phenotype under investigation. In such cases, employing the Louvain algorithm with a relatively high resolution, which is the default setting in SCIPAC, enables us to differentiate between these subtypes and allows SCIPAC to assign varying association strengths to each subtype.

SCIPAC fits the regression model using the elastic net, a machine-learning algorithm that maximizes a penalized version of the likelihood. The elastic net can be replaced by other penalized estimates of regression models, such as SCAD [78], without altering the rest of the SCIPAC algorithm. The combination of a regression model and a penalized estimation algorithm such as the elastic net has shown comparable or higher prediction power than other sophisticated methods such as random forests, boosting, or neural networks in numerous applications, especially for gene expression data [79]. However, there can still be datasets where other models have higher prediction power. It will be future work to incorporate these models into SCIPAC.

The use of metacells is becoming an efficient way to handle large single-cell datasets [80–83]. Conceptually, SCIPAC can incorporate metacells and their representatives as an alternative to its default setting of using cell clusters/types and their centroids. We have explored this aspect using metacells provided by SEACells [81]. Details are given in Additional file 1. Our comparative analysis reveals that combining SCIPAC with SEACells results in significantly reduced performance compared to using SCIPAC directly on original single-cell data. The primary reason for this appears to be the subpar performance of SEACells in cell grouping, especially when contrasted with the Louvain algorithm. Given these findings, we do not suggest using metacells provided by SEACells for SCIPAC applications in the current stage.

## Conclusions

SCIPAC is a novel algorithm for studying the associations between cells and phenotypes. Compared to the previous algorithm, SCIPAC gives a much more detailed and comprehensive description of the associations by enabling a quantitative estimation of the association strength and by providing a quality control—the *p*-value. Underlying SCIPAC are a general statistical model that accommodates virtually all types of phenotypes, including ordinal (and potentially count) phenotypes that have never been considered before, and a concise and closed-form mathematical formula that quantifies the association, which minimizes the computational load. The mathematical conciseness also largely frees SCIPAC from parameter tuning. The only parameter (i.e., the resolution) barely changes the results given by SCIPAC. Overall, compared with its predecessor, SCIPAC represents a substantially more capable software by being much more informative, versatile, robust, and user-friendly.

The improvement in accuracy is also remarkable. In simulated data, SCIPAC achieves high power and low false positives, which is evident from the UMAP plot, F1 score, and FSC score. In real data, SCIPAC gives results that are consistent with current biological knowledge for cell types whose functions are well understood. For cell types whose functions are less studied or more multifaceted, SCIPAC gives support to certain biological hypotheses or helps identify/discover cell sub-types.

## Methods

SCIPAC’s identification of cell-phenotype associations closely follows its definition of association: when increasing the fraction of a cell type increases (or decreases) the probability for a phenotype to be present, this cell type is positively (or negatively) associated with the phenotype.

### The increase of the fraction of a cell type

For a bulk sample, let vector $\varvec{G} \in \mathbb {R}^p$ be its expression profile, that is, its expression on the *p* genes. Suppose there are *K* cell types in the tissue, and let $\varvec{g}_{k}$ be the representative expression of the *k*’th cell type. Usually, people assume that $\varvec{G}$ can be decomposed by

1 $$\begin{aligned} \varvec{G} = \sum \limits _{k = 1}^{K}\gamma _{k}\varvec{g}_{k}, \end{aligned}$$

where $\gamma _{k}$ is the proportion of cell type *k* in the bulk tissue, with $\sum _{k = 1}^{K}\gamma _{k} = 1$. This equation links the bulk and single-cell expression data.

Now consider increasing cells from cell type *k* by $\Delta \gamma$ proportion of the original number of cells. Then, the new proportion of cell type *k* becomes $\frac{\gamma _{k} + \Delta \gamma }{1 + \Delta \gamma }$, and the new proportion of cell type $j \ne k$ becomes $\frac{\gamma _{j}}{1 + \Delta \gamma }$ (note that the new proportions of all cell types should still add up to 1). Thus, the bulk expression profile with the increase of cell type *k* becomes

$$\begin{aligned} \varvec{G}^* = \frac{\gamma _{k} + \Delta \gamma }{1 + \Delta \gamma }\varvec{g}_{k} + \sum \limits _{1\le j\le K, j\ne k}\frac{\gamma _{j}}{1 + \Delta \gamma }\varvec{g}_{j} = \frac{1}{1 + \Delta \gamma }\left(\sum \limits _{j = 1}^{K}\gamma _{j}\varvec{g}_{j} + \Delta \gamma \varvec{g}_{k}\right). \end{aligned}$$

Plugging Eq. 1, we get

2 $$\begin{aligned} \varvec{G}^* = \frac{1}{1 + \Delta \gamma }(\varvec{G} + \Delta \gamma \varvec{g}_{k}). \end{aligned}$$

Interestingly, this expression of $\varvec{G}^*$ does not include $\gamma _{1}, \ldots , \gamma _{K}$. This means that there is no need actually to compute $\gamma _{1}, \ldots , \gamma _{K}$ in Eq. 1, which could otherwise be done using a cell-type-decomposition software, but an accurate and robust decomposition is non-trivial [84–86]. See Additional file 1 for a more in-depth discussion on the connections of SCIPAC with decomposition/deconvolution.

### The change in chance of a phenotype

In this section, we consider how the increase in the fraction of a cell type will change the chance for a binary phenotype such as cancer to occur. Other types of phenotypes will be considered in the next section.

Let $\pi (\varvec{G})$ be the chance of an individual with gene expression profile $\varvec{G}$ for this phenotype to occur. We assume a logistic regression model to describe the relationship between $\pi (\varvec{G})$ and $\varvec{G}$:

3 $$\begin{aligned} \log \left( \frac{\pi (\varvec{G})}{1 - \pi (\varvec{G})}\right) = \beta _{0} + \varvec{\beta }^T\varvec{G}, \end{aligned}$$

here the left-hand side is the log odds of $\pi (\varvec{G})$, $\beta _{0}$ is the intercept, and $\varvec{\beta }$ is a length-*p* vector of coefficients. In the section after the next, we will describe how we obtain $\beta _{0}$ and $\varvec{\beta }$ from the data.

When increasing cells from cell type *k* by $\Delta \gamma$, $\varvec{G}$ becomes $\varvec{G}^*$ in Eq. 3. Plugging Eq. 2, we get

4 $$\begin{aligned} \log \left( \frac{\pi (\varvec{G}^*)}{1 - \pi (\varvec{G}^*)}\right) = \beta _{0} + \varvec{\beta }^T\frac{1}{1 + \Delta \gamma }(\varvec{G} + \Delta \gamma \varvec{g}_{k}). \end{aligned}$$

We further take the difference between Eqs. 4 and 3 and get

5 $$\begin{aligned} \log \left( \frac{\pi (\varvec{G}^*)}{1 - \pi (\varvec{G}^*)}\right) - \log \left( \frac{\pi (\varvec{G})}{1 - \pi (\varvec{G})}\right) = \frac{\Delta \gamma }{1 + \Delta \gamma }\varvec{\beta }^T(\varvec{g}_{k} - \varvec{G}). \end{aligned}$$

The left-hand side of this equation is the log odds ratio (i.e., the change of log odds). On the right-hand side, $\frac{\Delta \gamma }{1 + \Delta \gamma }$ is an increasing function with respect to $\Delta \gamma$, and $\varvec{\beta }^T(\varvec{g}_{k} - \varvec{G})$ is independent of $\Delta \gamma$. This indicates that given any specific $\Delta \gamma$, the log odds ratio under over-representation of cell type *k* is proportional to

6 $$\begin{aligned} \lambda _k = \varvec{\beta }^T(\varvec{g}_{k} - \varvec{G}) . \end{aligned}$$

$\lambda _k$ describes the strength of the effect of increasing cell type *k* to a bulk sample with expression profile $\varvec{G}$. Given the presence of numerous bulk samples, employing multiple $\lambda _k$’s could be cumbersome and obscure the overall effect of a particular cell type. To concisely summarize the association of cell type *k*, we propose averaging their effects. The average effect on all bulk samples can be obtained by

7 $$\begin{aligned} \Lambda _k = \varvec{\beta }^T(\varvec{g}_{k} - \bar{\varvec{G}}) , \end{aligned}$$

where $\bar{\varvec{G}}$ is the average expression profile of all bulk samples.

$\Lambda _k$ gives an overall impression of how strong the effect is when cell type *k* over-represents to the probability for the phenotype to be present. Its sign represents the direction of the change: a positive value means an increase in probability, and a negative value means a decrease in probability. Its absolute value represents the strength of the effect. In SCIPAC, we call $\Lambda _k$ the association strength of cell type *k* and the phenotype.

Note that this derivation does not involve likelihood, although the computation of $\varvec{\beta }$ does. Here, it serves more as a definitional approach.

### Definition of the association strength for other types of phenotype

Our definition of $\Lambda _k$ relies on vector $\varvec{\beta }$. In the case of a binary phenotype, $\varvec{\beta }$ are the coefficients of a logistic regression that describes a linear relationship between the expression profile and the log odds of having the phenotype, as shown in Eq. 3. For other types of phenotype, $\varvec{\beta }$ can be defined/computed similarly.

For a quantitative (i.e., continuous) phenotype, an ordinary linear regression can be used, and the left-hand side of Eq. 3 is changed to the quantitative value of the phenotype.

For a survival phenotype, a Cox proportional hazards model can be used, and the left-hand side of Eq. 3 is changed to the log hazard ratio.

For an ordinal phenotype, we use a proportional odds model

$$\begin{aligned} \log \frac{\Pr (Y_{i} \ge j + 1 | X)}{1 - \Pr (Y_{i} \ge j + 1 | X)} = \beta _{0, j} + \varvec{\beta }^T\varvec{x}_{i}, \end{aligned}$$

where $j \in \{1, 2, ..., (J - 1)\}$ and *J* is the number of ordinal levels. It should be noted that here we use the right-tail probability $\Pr (Y_{i} \ge j + 1 | X)$ instead of the commonly used cumulative probability (left-tail probability) $\Pr (Y_{i} \le j | X)$. Such a change makes the interpretation consistent with other types of phenotypes: in our model, a larger value on the right-hand side indicates a larger chance for $Y_{i}$ to have a higher level, which in turn guarantees that the sign of the association strength defined according to this $\varvec{\beta }$ has the usual meaning: a positive $\Lambda _k$ value means a positive association with the phenotype-using the cancer stage as an example. A positive $\Lambda _k$ means the over-representation of cell type *k* increases the chance of a higher cancer stage. In contrast, using the commonly used cumulative probability leads to a counter-intuitive, reversed interpretation.

### Computation of the association strength in practice

In practice, $\varvec{\beta }$ in Eq. 3 needs to be learned from the bulk data. By default, SCIPAC uses the elastic net, a popular and powerful penalized regression method:

$$\begin{aligned} \min _{\beta _{0}, \varvec{\beta }} - \frac{1}{n}l(\beta _{0}, \varvec{\beta }) + \lambda \Bigl \{ \frac{1 - \alpha }{2}||\varvec{\beta }||_{2}^2 + \alpha ||\varvec{\beta }||_{1}\Bigl \}. \end{aligned}$$

In this model, $l(\beta _{0}, \varvec{\beta })$ is a log-likelihood of the linear model (i.e., logistic regression for a binary phenotype, ordinary linear regression for a quantitative phenotype, Cox proportional odds model for a survival phenotype, and proportional odds model for an ordinal phenotype). $\alpha$ is a number between 0 and 1, denoting a combination of $\ell _1$ and $\ell _2$ penalties, and $\lambda$ is the penalty strength. SCIPAC fixes $\alpha$ to be 0.4 (see Additional file 1 for discussions on this choice) and uses 10-fold cross-validation to decide $\lambda$ automatically. This way, they do not become hyperparameters.

In SCIPAC, the fitting and cross-validation of the elastic net are done by calling the *ordinalNet* [87] R package for the ordinal phenotype and by calling the *glmnet* R package [88–91] for other types of phenotypes.

The computation of the association strength, as defined by Eq. 7, does not only require $\varvec{\beta }$, but also $\varvec{g}_k$ and $\bar{\varvec{G}}$. $\bar{\varvec{G}}$ is simply the average expression profile of all bulk samples. On the other hand, $\varvec{g}_k$ requires knowing the cell type of each cell. By default, SCIPAC does not assume this information to be given, and it uses the Louvain clustering implemented in the Seurat [24, 25] R package to infer it. This clustering algorithm has one tuning parameter called “resolution.” SCIPAC sets its default value as 2.0, and the user can use other values. With the inferred or given cell types, $\varvec{g}_k$ is computed as the centroid (i.e., the mean expression profile) of cells in cluster *k*.

Given $\varvec{\beta }$, $\bar{\varvec{G}}$, and $\varvec{g}_k$, the association strength can be computed using Eq. 7. Knowing the association strength for each cell type and the cell-type label for each cell, we also know the association strength for every single cell. In practice, we standardize the association strengths for all cells. That is, we compute the mean and standard deviation of the association strengths of all cells and use them to centralize and scale the association strength, respectively. We have found such standardization makes SCIPAC more robust to the possible unbalance in sample size of bulk data in different phenotype groups.

### Computation of the *p*-value

SCIPAC uses non-parametric bootstrap [92] to compute the standard deviation and hence the *p*-value of the association. Fifty bootstrap samples, which are believed to be enough to compute the standard error of most statistics [93], are generated for the bulk expression data, and each is used to compute (standardized) $\Lambda$ values for all the cells. For cell *i*, let its original $\Lambda$ values be $\Lambda _i$, and the bootstrapped values be $\Lambda _i^{(1)}, \ldots , \Lambda _i^{(50)}$. A *z*-score is then computed using

$$\begin{aligned} z_i = \frac{\Lambda _i}{\text{ standard } \text{ deviation }\left(\Lambda _i^{(1)}, \ldots , \Lambda _i^{(50)}\right)}, \end{aligned}$$

and then the *p*-value is computed according to the cumulative distribution function of the standard Gaussian distribution. See Additional file 1 for more discussions on the calculation of *p*-value.

### Supplementary information

Additional file 1. Supplementary Materials that include additional results and plots.

Additional file 2. A vignette of the SCIPAC package.

Additional file 3. Review history.

## Data Availability

The simulated datasets [94] under three schemes are available at Zenodo with DOI 10.5281/zenodo.11013320 [95]. The SCIPAC package is available at GitHub website https://github.com/RavenGan/SCIPAC under the MIT license [96]. The source code of SCIPAC is also deposited at Zenodo with DOI 10.5281/zenodo.11013696 [97]. A vignette of the R package is available on the GitHub page and in the Additional file 2. The prostate cancer scRNA-seq data is obtained from the Prostate Cell Atlas https://www.prostatecellatlas.org [29]; the scRNA-seq data for the breast cancer are from the Gene Expression Omnibus (GEO) under accession number GSE176078 [34, 98]; the scRNA-seq data for the lung cancer are from E-MTAB-6149 [99] and E-MTAB-6653 [71, 100]; the scRNA-seq data for facioscapulohumeral muscular dystrophy data are from the GEO under accession number GSE122873 [101]. The bulk RNA-seq data are obtained from the TCGA database via TCGAbiolinks (ver. 2.25.2) R package [102]. More details about the simulated and real scRNA-seq and bulk RNA-seq data can be found in the Additional file 1.

## References

[1] Yofe I, Dahan R, Amit I (2020). Single-cell genomic approaches for developing the next generation of immunotherapies. Nat Med..

[2] Zhang Q, He Y, Luo N, Patel SJ, Han Y, Gao R (2019). Landscape and dynamics of single immune cells in hepatocellular carcinoma. Cell..

[3] Fan J, Slowikowski K, Zhang F (2020). Single-cell transcriptomics in cancer: computational challenges and opportunities. Exp Mol Med..

[4] Klein AM, Mazutis L, Akartuna I, Tallapragada N, Veres A, Li V (2015). Droplet barcoding for single-cell transcriptomics applied to embryonic stem cells. Cell..

[5] Macosko EZ, Basu A, Satija R, Nemesh J, Shekhar K, Goldman M (2015). Highly parallel genome-wide expression profiling of individual cells using nanoliter droplets. Cell..

[6] Rosenberg AB, Roco CM, Muscat RA, Kuchina A, Sample P, Yao Z (2018). Single-cell profiling of the developing mouse brain and spinal cord with split-pool barcoding. Science..

[7] Zheng GX, Terry JM, Belgrader P, Ryvkin P, Bent ZW, Wilson R (2017). Massively parallel digital transcriptional profiling of single cells. Nat Commun..

[8] Abdelaal T, Michielsen L, Cats D, Hoogduin D, Mei H, Reinders MJ (2019). A comparison of automatic cell identification methods for single-cell RNA sequencing data. Genome Biol..

[9] Luecken MD, Theis FJ (2019). Current best practices in single-cell RNA-seq analysis: a tutorial. Mol Syst Biol..

[10] Guo H, Li J (2021). scSorter: assigning cells to known cell types according to marker genes. Genome Biol..

[11] Pliner HA, Shendure J, Trapnell C (2019). Supervised classification enables rapid annotation of cell atlases. Nat Methods..

[12] Zhang AW, O’Flanagan C, Chavez EA, Lim JL, Ceglia N, McPherson A (2019). Probabilistic cell-type assignment of single-cell RNA-seq for tumor microenvironment profiling. Nat Methods..

[13] Zhang Z, Luo D, Zhong X, Choi JH, Ma Y, Wang S (2019). SCINA: a semi-supervised subtyping algorithm of single cells and bulk samples. Genes..

[14] Johnson TS, Wang T, Huang Z, Yu CY, Wu Y, Han Y (2019). LAmbDA: label ambiguous domain adaptation dataset integration reduces batch effects and improves subtype detection. Bioinformatics..

[15] Ma F, Pellegrini M (2020). ACTINN: automated identification of cell types in single cell RNA sequencing. Bioinformatics..

[16] Tan Y, Cahan P (2019). SingleCellNet: a computational tool to classify single cell RNA-Seq data across platforms and across species. Cell Syst..

[17] Salcher S, Sturm G, Horvath L, Untergasser G, Kuempers C, Fotakis G (2022). High-resolution single-cell atlas reveals diversity and plasticity of tissue-resident neutrophils in non-small cell lung cancer. Cancer Cell..

[18] Good Z, Sarno J, Jager A, Samusik N, Aghaeepour N, Simonds EF (2018). Single-cell developmental classification of B cell precursor acute lymphoblastic leukemia at diagnosis reveals predictors of relapse. Nat Med..

[19] Wagner J, Rapsomaniki MA, Chevrier S, Anzeneder T, Langwieder C, Dykgers A (2019). A single-cell atlas of the tumor and immune ecosystem of human breast cancer. Cell..

[20] Weinstein JN, Collisson EA, Mills GB, Shaw KR, Ozenberger BA, Ellrott K (2013). The cancer genome atlas pan-cancer analysis project. Nat Genet..

[21] Cerami E, Gao J, Dogrusoz U, Gross BE, Sumer SO, Aksoy BA (2012). The cBio cancer genomics portal: an open platform for exploring multidimensional cancer genomics data. Cancer Disc..

[22] Gao J, Aksoy BA, Dogrusoz U, Dresdner G, Gross B, Sumer SO (2013). Integrative analysis of complex cancer genomics and clinical profiles using the cBioPortal. Sci Signal..

[23] Sun D, Guan X, Moran AE, Wu LY, Qian DZ, Schedin P (2022). Identifying phenotype-associated subpopulations by integrating bulk and single-cell sequencing data. Nat Biotechnol..

[24] Blondel VD, Guillaume JL, Lambiotte R, Lefebvre E (2008). Fast unfolding of communities in large networks. J Stat Mech Theory Exp..

[25] Stuart T, Butler A, Hoffman P, Hafemeister C, Papalexi E, Mauck WM (2019). Comprehensive integration of single-cell data. Cell..

[26] Zou H, Hastie T (2005). Regularization and variable selection via the elastic net. J R Stat Soc Ser B Stat Methodol..

[27] McInnes L, Healy J, Melville J. UMAP: Uniform Manifold Approximation and Projection for Dimension Reduction. 2018. arXiv preprint arXiv:1802.03426.

[28] Wong CJ, Wang LH, Friedman SD, Shaw D, Campbell AE, Budech CB (2020). Longitudinal measures of RNA expression and disease activity in FSHD muscle biopsies. Hum Mol Genet..

[29] Tuong ZK, Loudon KW, Berry B, Richoz N, Jones J, Tan X (2021). Resolving the immune landscape of human prostate at a single-cell level in health and cancer. Cell Rep..

[30] Hume DA (2006). The mononuclear phagocyte system. Curr Opin Immunol..

[31] Hume DA, Ross IL, Himes SR, Sasmono RT, Wells CA, Ravasi T (2002). The mononuclear phagocyte system revisited. J Leukoc Biol..

[32] Raggi F, Bosco MC (2020). Targeting mononuclear phagocyte receptors in cancer immunotherapy: New perspectives of the triggering receptor expressed on myeloid cells (TREM-1). Cancers..

[33] Largeot A, Pagano G, Gonder S, Moussay E, Paggetti J (2019). The B-side of cancer immunity: the underrated tune. Cells..

[34] Wu SZ, Al-Eryani G, Roden DL, Junankar S, Harvey K, Andersson A (2021). A single-cell and spatially resolved atlas of human breast cancers. Nat Genet..

[35] Fernández-Nogueira P, Fuster G, Gutierrez-Uzquiza Á, Gascón P, Carbó N, Bragado P (2021). Cancer-associated fibroblasts in breast cancer treatment response and metastasis. Cancers..

[36] Ao Z, Shah SH, Machlin LM, Parajuli R, Miller PC, Rawal S (2015). Identification of Cancer-Associated Fibroblasts in Circulating Blood from Patients with Metastatic Breast CancerIdentification of cCAFs from Metastatic Cancer Patients. Cancer Res..

[37] Arcucci A, Ruocco MR, Granato G, Sacco AM, Montagnani S. Cancer: an oxidative crosstalk between solid tumor cells and cancer associated fibroblasts. BioMed Res Int. 2016;2016. https://pubmed.ncbi.nlm.nih.gov/27595103/. 10.1155/2016/4502846 PMC4993917 27595103

[38] Buchsbaum RJ, Oh SY (2016). Breast cancer-associated fibroblasts: where we are and where we need to go. Cancers..

[39] Ruocco MR, Avagliano A, Granato G, Imparato V, Masone S, Masullo M (2018). Involvement of breast cancer-associated fibroblasts in tumor development, therapy resistance and evaluation of potential therapeutic strategies. Curr Med Chem..

[40] Savas P, Virassamy B, Ye C, Salim A, Mintoff CP, Caramia F (2018). Single-cell profiling of breast cancer T cells reveals a tissue-resident memory subset associated with improved prognosis. Nat Med..

[41] Bassez A, Vos H, Van Dyck L, Floris G, Arijs I, Desmedt C (2021). A single-cell map of intratumoral changes during anti-PD1 treatment of patients with breast cancer. Nat Med..

[42] Romero JM, Grünwald B, Jang GH, Bavi PP, Jhaveri A, Masoomian M (2020). A Four-Chemokine Signature Is Associated with a T-cell-Inflamed Phenotype in Primary and Metastatic Pancreatic CancerChemokines in Pancreatic Cancer. Clin Cancer Res..

[43] Tamura R, Yoshihara K, Nakaoka H, Yachida N, Yamaguchi M, Suda K (2020). XCL1 expression correlates with CD8-positive T cells infiltration and PD-L1 expression in squamous cell carcinoma arising from mature cystic teratoma of the ovary. Oncogene..

[44] Hernandez R, Põder J, LaPorte KM, Malek TR. Engineering IL-2 for immunotherapy of autoimmunity and cancer. Nat Rev Immunol. 2022:22:1–15. https://pubmed.ncbi.nlm.nih.gov/35217787/. 10.1038/s41577-022-00680-w 35217787

[45] Korotkevich G, Sukhov V, Budin N, Shpak B, Artyomov MN, Sergushichev A. Fast gene set enrichment analysis. BioRxiv. 2016:060012. https://www.biorxiv.org/content/10.1101/060012v3.abstract.

[46] Dang CV (2012). MYC on the path to cancer. Cell..

[47] Gnanaprakasam JR, Wang R (2017). MYC in regulating immunity: metabolism and beyond. Genes..

[48] Oshi M, Takahashi H, Tokumaru Y, Yan L, Rashid OM, Matsuyama R (2020). G2M cell cycle pathway score as a prognostic biomarker of metastasis in estrogen receptor (ER)-positive breast cancer. Int J Mol Sci..

[49] Zhang X, Lan Y, Xu J, Quan F, Zhao E, Deng C (2019). Cell Marker: a manually curated resource of cell markers in human and mouse. Nucleic Acids Res..

[50] Chen L, Yang L, Qiao F, Hu X, Li S, Yao L (2015). High levels of nucleolar spindle-associated protein and reduced levels of BRCA1 expression predict poor prognosis in triple-negative breast cancer. PLoS ONE..

[51] Li M, Yang B. Prognostic value of NUSAP1 and its correlation with immune infiltrates in human breast cancer. Crit Rev^TM^ Eukaryot Gene Expr. 2022;32(3). https://pubmed.ncbi.nlm.nih.gov/35695609/. 10.1615/CritRevEukaryotGeneExpr.2021040248 35695609

[52] Zhang X, Pan Y, Fu H, Zhang J (2018). Nucleolar and spindle associated protein 1 (NUSAP1) inhibits cell proliferation and enhances susceptibility to epirubicin in invasive breast cancer cells by regulating cyclin D kinase (CDK1) and DLGAP5 expression. Med Sci Monit: Int Med J Exp Clin Res..

[53] Geyer FC, Rodrigues DN, Weigelt B, Reis-Filho JS (2012). Molecular classification of estrogen receptor-positive/luminal breast cancers. Adv Anat Pathol..

[54] Karamitopoulou E, Perentes E, Tolnay M, Probst A (1998). Prognostic significance of MIB-1, p53, and bcl-2 immunoreactivity in meningiomas. Hum Pathol..

[55] Duxbury MS, Whang EE. RRM2 induces NF-$$\kappa$$B-dependent MMP-9 activation and enhances cellular invasiveness. Biochem Biophys Res Commun. 2007;354(1):190–6. 10.1016/j.bbrc.2006.12.177 17222798

[56] Zhou BS, Tsai P, Ker R, Tsai J, Ho R, Yu J (1998). Overexpression of transfected human ribonucleotide reductase M2 subunit in human cancer cells enhances their invasive potential. Clin Exp Metastasis..

[57] Zhang H, Liu X, Warden CD, Huang Y, Loera S, Xue L (2014). Prognostic and therapeutic significance of ribonucleotide reductase small subunit M2 in estrogen-negative breast cancers. BMC Cancer..

[58] Putluri N, Maity S, Kommagani R, Creighton CJ, Putluri V, Chen F (2014). Pathway-centric integrative analysis identifies RRM2 as a prognostic marker in breast cancer associated with poor survival and tamoxifen resistance. Neoplasia..

[59] Koleck TA, Conley YP (2016). Identification and prioritization of candidate genes for symptom variability in breast cancer survivors based on disease characteristics at the cellular level. Breast Cancer Targets Ther..

[60] Li Jp, Zhang Xm, Zhang Z, Zheng Lh, Jindal S, Liu Yj. Association of p53 expression with poor prognosis in patients with triple-negative breast invasive ductal carcinoma. Medicine. 2019;98(18). https://pubmed.ncbi.nlm.nih.gov/31045815/. 10.1097/MD.0000000000015449 PMC6504250 31045815

[61] Gong MT, Ye SD, Lv WW, He K, Li WX (2018). Comprehensive integrated analysis of gene expression datasets identifies key anti-cancer targets in different stages of breast cancer. Exp Ther Med..

[62] Chen Wx, Yang Lg, Xu Ly, Cheng L, Qian Q, Sun L, et al. Bioinformatics analysis revealing prognostic significance of RRM2 gene in breast cancer. Biosci Rep. 2019;39(4). https://pubmed.ncbi.nlm.nih.gov/30898978/. 10.1042/BSR20182062 PMC6454020 30898978

[63] Hao Z, Zhang H, Cowell J (2012). Ubiquitin-conjugating enzyme UBE2C: molecular biology, role in tumorigenesis, and potential as a biomarker. Tumor Biol..

[64] Arriola E, Rodriguez-Pinilla SM, Lambros MB, Jones RL, James M, Savage K (2007). Topoisomerase II alpha amplification may predict benefit from adjuvant anthracyclines in HER2 positive early breast cancer. Breast Cancer Res Treat..

[65] Knoop AS, Knudsen H, Balslev E, Rasmussen BB, Overgaard J, Nielsen KV (2005). Retrospective analysis of topoisomerase IIa amplifications and deletions as predictive markers in primary breast cancer patients randomly assigned to cyclophosphamide, methotrexate, and fluorouracil or cyclophosphamide, epirubicin, and fluorouracil: Danish Breast Cancer Cooperative Group. J Clin Oncol..

[66] Tanner M, Isola J, Wiklund T, Erikstein B, Kellokumpu-Lehtinen P, Malmstrom P, et al. Topoisomerase II$$\alpha$$ gene amplification predicts favorable treatment response to tailored and dose-escalated anthracycline-based adjuvant chemotherapy in HER-2/neu-amplified breast cancer: Scandinavian Breast Group Trial 9401. J Clin Oncol. 2006;24(16):2428–36. 10.1200/JCO.2005.02.9264 16682728

[67] Arriola E, Moreno A, Varela M, Serra JM, Falo C, Benito E, et al. Predictive value of HER-2 and topoisomerase II$$\alpha$$ in response to primary doxorubicin in breast cancer. Eur J Cancer. 2006;42(17):2954–60. 10.1016/j.ejca.2006.06.013 16935488

[68] Järvinen TA, Tanner M, Bärlund M, Borg Å, Isola J. Characterization of topoisomerase II$$\alpha$$ gene amplification and deletion in breast cancer. Gene Chromosome Cancer. 1999;26(2):142–50. 10469452

[69] Landberg G, Erlanson M, Roos G, Tan EM, Casiano CA (1996). Nuclear autoantigen p330d/CENP-F: a marker for cell proliferation in human malignancies. Cytom J Int Soc Anal Cytol..

[70] Bettelli E, Carrier Y, Gao W, Korn T, Strom TB, Oukka M (2006). Reciprocal developmental pathways for the generation of pathogenic effector TH17 and regulatory T cells. Nature..

[71] Lambrechts D, Wauters E, Boeckx B, Aibar S, Nittner D, Burton O (2018). Phenotype molding of stromal cells in the lung tumor microenvironment. Nat Med..

[72] Bremnes RM, Busund LT, Kilvær TL, Andersen S, Richardsen E, Paulsen EE (2016). The role of tumor-infiltrating lymphocytes in development, progression, and prognosis of non-small cell lung cancer. J Thorac Oncol..

[73] Schalper KA, Brown J, Carvajal-Hausdorf D, McLaughlin J, Velcheti V, Syrigos KN, et al. Objective measurement and clinical significance of TILs in non–small cell lung cancer. J Natl Cancer Inst. 2015;107(3):dju435. 10.1093/jnci/dju435 PMC4565530 25650315

[74] Tay RE, Richardson EK, Toh HC. Revisiting the role of CD4+ T cells in cancer immunotherapy—new insights into old paradigms. Cancer Gene Ther. 2021;28(1):5–17. 10.1038/s41417-020-0183-x PMC7886651 32457487

[75] Dieu-Nosjean MC, Goc J, Giraldo NA, Sautès-Fridman C, Fridman WH (2014). Tertiary lymphoid structures in cancer and beyond. Trends Immunol..

[76] Wang Ss, Liu W, Ly D, Xu H, Qu L, Zhang L. Tumor-infiltrating B cells: their role and application in anti-tumor immunity in lung cancer. Cell Mol Immunol. 2019;16(1):6–18. 10.1038/s41423-018-0027-x PMC6318290 29628498

[77] van den Heuvel A, Mahfouz A, Kloet SL, Balog J, van Engelen BG, Tawil R (2019). Single-cell RNA sequencing in facioscapulohumeral muscular dystrophy disease etiology and development. Hum Mol Genet..

[78] Fan J, Li R (2001). Variable selection via nonconcave penalized likelihood and its oracle properties. J Am Stat Assoc..

[79] Hastie T, Tibshirani R, Friedman JH, Friedman JH (2009). The elements of statistical learning: data mining, inference, and prediction.

[80] Baran Y, Bercovich A, Sebe-Pedros A, Lubling Y, Giladi A, Chomsky E (2019). MetaCell: analysis of single-cell RNA-seq data using K-nn graph partitions. Genome Biol..

[81] Persad S, Choo ZN, Dien C, Sohail N, Masilionis I, Chaligné R, et al. SEACells infers transcriptional and epigenomic cellular states from single-cell genomics data. Nat Biotechnol. 2023;41:1–12. https://pubmed.ncbi.nlm.nih.gov/36973557/. 10.1038/s41587-023-01716-9 PMC10713451 36973557

[82] Ben-Kiki O, Bercovich A, Lifshitz A, Tanay A (2022). Metacell-2: a divide-and-conquer metacell algorithm for scalable scRNA-seq analysis. Genome Biol..

[83] Bilous M, Tran L, Cianciaruso C, Gabriel A, Michel H, Carmona SJ (2022). Metacells untangle large and complex single-cell transcriptome networks. BMC Bioinformatics..

[84] Avila Cobos F, Alquicira-Hernandez J, Powell JE, Mestdagh P, De Preter K (2020). Benchmarking of cell type deconvolution pipelines for transcriptomics data. Nat Commun..

[85] Jin H, Liu Z (2021). A benchmark for RNA-seq deconvolution analysis under dynamic testing environments. Genome Biol..

[86] Wang X, Park J, Susztak K, Zhang NR, Li M (2019). Bulk tissue cell type deconvolution with multi-subject single-cell expression reference. Nat Commun..

[87] Wurm MJ, Rathouz PJ, Hanlon BM. Regularized ordinal regression and the ordinalNet R package. 2017. arXiv preprint arXiv:1706.05003. 10.18637/jss.v099.i06 PMC8432594 34512213

[88] Friedman J, Hastie T, Tibshirani R (2010). Regularization paths for generalized linear models via coordinate descent. J Stat Softw..

[89] Simon N, Friedman J, Hastie T. A blockwise descent algorithm for group-penalized multiresponse and multinomial regression. 2013. arXiv preprint arXiv:1311.6529.

[90] Simon N, Friedman J, Hastie T, Tibshirani R (2011). Regularization paths for Cox’s proportional hazards model via coordinate descent. J Stat Softw..

[91] Tibshirani R, Bien J, Friedman J, Hastie T, Simon N, Taylor J (2012). Strong rules for discarding predictors in lasso-type problems. J R Stat Soc Ser B Stat Methodol..

[92] Efron B. Bootstrap methods: another look at the jackknife. In: Breakthroughs in statistics. New York: Springer; 1992. pp. 569–593.

[93] Efron B, Tibshirani RJ (1994). An introduction to the bootstrap.

[94] Zappia L, Phipson B, Oshlack A (2017). Splatter: simulation of single-cell RNA sequencing data. Genome Biol..

[95] Gan D, Zhu Y, Lu X, Li J (2024). Simulated datasets used in SCIPAC analysis. Zenodo..

[96] Gan D, Zhu Y, Lu X, Li J. SCIPAC R package. GitHub. 2024. https://github.com/RavenGan/SCIPAC. Accessed 24 Apr 2024.

[97] Gan D, Zhu Y, Lu X, Li J. SCIPAC source code. Zenodo. 2024. 10.5281/zenodo.11013696.

[98] Wu SZ, Al-Eryani G, Roden DL, Junankar S, Harvey K, Andersson A, et al. A single-cell and spatially resolved atlas of human breast cancers. Datasets. 2021. https://www.ncbi.nlm.nih.gov/geo/query/acc.cgi?acc=GSE176078. Gene Expression Omnibus. Accessed 1 Oct 2022. 10.1038/s41588-021-00911-1 PMC9044823 34493872

[99] Lambrechts D, Wauters E, Boeckx B, Aibar S, Nittner D, Burton O, et al. Phenotype molding of stromal cells in the lung tumor microenvironment. Datasets. 2018. https://www.ebi.ac.uk/biostudies/arrayexpress/studies/E-MTAB-6149. ArrayExpress. Accessed 24 July 2022. 10.1038/s41591-018-0096-5 29988129

[100] Lambrechts D, Wauters E, Boeckx B, Aibar S, Nittner D, Burton O, et al. Phenotype molding of stromal cells in the lung tumor microenvironment. Datasets. 2018. https://www.ebi.ac.uk/biostudies/arrayexpress/studies/E-MTAB-6653. ArrayExpress. Accessed 24 July 2022. 10.1038/s41591-018-0096-5 29988129

[101] van den Heuvel A, Mahfouz A, Kloet SL, Balog J, van Engelen BG, Tawil R, et al. Single-cell RNA sequencing in facioscapulohumeral muscular dystrophy disease etiology and development. Datasets. 2019. https://www.ncbi.nlm.nih.gov/geo/query/acc.cgi?acc=GSE122873. Gene Expression Omnibus. Accessed 13 Aug 2022. 10.1093/hmg/ddy400 PMC6423425 30445587

[102] Colaprico A, Silva TC, Olsen C, Garofano L, Cava C, Garolini D (2016). TCGAbiolinks: an R/Bioconductor package for integrative analysis of TCGA data. Nucleic Acids Res..

