# Supplementary material for: SCIPAC: quantitative estimation of cell-phenotype associations
Source: Genome Biol. 2024 May 13;25:119. doi: 10.1186/s13059-024-03263-1 (PMC11089691; doi:10.1186/s13059-024-03263-1)
Supplement: Supplementary file 1 — Additional file 1. Supplementary Materials that include additional results and plots. [file 13059_2024_3263_MOESM1_ESM.pdf]

Supplementary Material for Quantitative estimation of cell-phenotype associations

1 Simulation setup

We use *Splatter* R package [1] to simulate scRNA-seq data and use the scRNA-seq data to create the corresponding bulk RNA-seq data. These data are then pre-processed following the same procedure (described in the next section) as that for the real datasets. For example, only 1,000 high-variable genes are retained for further analysis.

1.1 Scheme I

We simulate 10,000 cells and 10,000 genes. There are three simulated cell types with group probabilities of 0.8, 0.1, and 0.1, respectively, resulting in one large cell population with around 8,000 cells and two small cell populations with around 1,000 cells each. The probabilities for a gene to be differentially expressed in the three cell types are set as 0.1, 0.01, and 0.01, respectively. Then, we assign these cells to two different phenotypes, named phenotype I and II. To achieve this, we randomly choose half of the cells from the large cell population and combine them with one of the small cell populations to form a phenotype. By doing so, the two small cell populations are unique to each phenotype and can be viewed as the ground truth of phenotype-associated cell populations. To simulate the expression profile of bulk samples for a phenotype, we randomly select 10,000 cells with replacement from the phenotype and take the average of their expressions. In this way, we generate bulk data with 100 samples, 50 with phenotype I and 50 with phenotype II. These two phenotypes correspond to the presence and absence of a binary phenotype.

1.2 Scheme II

We simulate 10,000 cells and 10,000 genes. There are seven simulated cell types with group probability 0.1, 0.1, 0.1, 0.1, 0.2, 0.2, and 0.2, respectively, resulting in four cell types with roughly 1,000 cells each and three cells types with roughly 2,000 cells each. The probabilities for a gene to be differentially expressed in the seven cell types are set as 0.01, 0.01, 0.1, 0.1, 0.15, 0.15, and 0.15, respectively. To assign cell types to two different phenotypes, named phenotype I and II, we perform a similar procedure as that in Scheme I. For phenotype I, we randomly choose half of the cells from cell types 5, 6, and 7 and combine them with cell types 1 and 3. For phenotype II, we combine the remaining half of the cells from cell types 5, 6, and 7 with cell types 2 and 4. By doing so, cell types 1 and 3 are unique to phenotype I, cell types 2 and 4 are unique to phenotype II, and they can be viewed as the ground truth of phenotype-associated cell populations. To simulate the expression profile of bulk samples for a phenotype, we randomly select 10,000 cells with replacement from each phenotype and take the average of their expressions. In this way, we generate bulk data with 200 samples, 100 with phenotype I and 100 with phenotype II. These two phenotypes correspond to the presence and absence of a binary phenotype.

1.3 Scheme III

We simulate 10,000 cells and 10,000 genes. There are four simulated paths with group probability of 0.25, 0.25, 0.25, and 0.25, respectively, resulting in roughly 2,500 cells for each path. The probability of a gene being differentially expressed is set to be 0.5 for all four paths. All four paths start from the same location, and the maximal number of steps is set to be 2,000 to discretize the cell developmental stages. To create ordinal phenotype, we treat cells with steps between 0 and 500 as Stage I cells, steps between 501 and 1,000 as Stage II cells, steps between 1,001 and 1,500 as Stage III cells, and steps between 1,501 and 2,000 as Stage IV cells. To simulate the expression profile of bulk samples for each level, we randomly select 10,000 cells with replacement from each level and take the average of their expressions. In this way, we generate bulk data with 200 samples, 50 coming from each stage.

2 Pre-processing of the single-cell and bulk RNA-sequencing data

We use genes present in both the scRNA-seq and bulk RNA-seq data. We apply functions in *Seurat* (ver. 4.1.1) R package [2] to pre-process the scRNA-seq data. We filter out genes with low expressions in cells and normalize the filtered expression matrix using the NormalizeData function with the default parameters. We then use the FindVariableFeatures function with the default "vst" method to identify 1,000 highly variable genes. We use these 1,000 genes for both the scRNA-seq and bulk RNA-seq data. For dimension reduction, we perform the principal component analysis (PCA) on the bulk RNA-seq data and use the same rotation matrix obtained from PCA on the scRNA-seq data. Performing PCA on the bulk RNA-seq data requires much less time to obtain the dimension-reduced data matrices. The first 60 PCs are kept in both the scRNA-seq and bulk RNA-seq data for downstream analysis. If the sample size of the bulk RNA-seq data is less than 60, it is impossible to use 60 PCs on the bulk RNA-seq data, and hence we first perform PCA on the scRNA-seq data and then use the rotation matrix on the bulk RNA-seq data.

3 Definition of the F1 score and FSC

Suppose the numbers of truly positively associated cells that are identified as positively associated, negatively associated, and null are m_{11}, m_{12}, and m_{10}, respectively. Suppose the numbers of truly negatively associated cells that are identified as positively associated, negatively associated, and null are m_{21}, m_{22}, and m_{20}, respectively. Suppose the numbers of truly null cells that are identified as positively associated, negatively associated, and null are m_{01}, m_{02}, and m_{00}, respectively. Then precision = $(m_{11}+m_{12}+m_{21}+m_{22})/(m_{11}+m_{12}+m_{21}+m_{22}+m_{01}+m_{02})$, recall = $(m_{11}+m_{12}+m_{21}+m_{22})/(m_{11}+m_{12}+m_{21}+m_{22}+m_{10}+m_{20})$, and F1 = $2\times$ precision $\times$ recall$/($precision $+$ recall$)$. FSC=$(m_{11}+m_{22})/(m_{11}+m_{12}+m_{21}+m_{22})$.

4 Other details about analyses

We apply the uniform manifold approximation and projection (UMAP) algorithm [3, 1] to visualize cells in a two-dimensional space. For all the analyses, we use the cosine distance metric with $k = 30$ nearest neighbors and min_dist = 0.3.

The pathway enrichment analysis is performed by using *fgsea* (ver. 1.20.0) R package

[4]. The input pre-decided gene rank in the fgsea function is evaluated by calculating the log2 fold change. The hallmark gene sets are obtained from *msigdb* (ver. 1.2.0) R package [5].

The bulk RNA-seq data of the three cancer types are obtained from the TCGA database via *TCGAbiolinks* (ver. 2.25.2) R package [6], while the bulk data of the muscular dystrophy is obtained from a published paper [7].

DE analyses are done using the FindMarkers function in *Seurat* with the default Wilcoxon rank sum test. The volcano plot is obtained by using *EnhancedVolcano* R package [8].

For Scissor, we follow the suggested pipeline from the primary GitHub repository (ver. 2.0.0) [9]. We set the family parameter to be "cox" or "binomial" in our comparison.

5 Data description

Prostate cancer data The scRNA-seq data for the prostate cancer are from [10]. The original study contains both normal and tumor tissues, and we use the tumor tissues with 8,700 cells from different cell types. The bulk RNA-seq data are obtained from the TCGA database via *TCGAbiolinks* (ver. 2.25.2) R package [6]. There are 550 TCGA-PRAD bulk samples with sample types (Cancer vs. normal tissues) information.

Breast cancer data The scRNA-seq data for the breast cancer are from [11]. The original study contains 11 ER+, five HER2+, and 10 TNBC breast tumor tissues, and we use the five HER2+ tumor tissues with 19,311 cells from different cell types. The bulk RNA-seq data are obtained from the TCGA database via *TCGAbiolinks* (ver. 2.25.2) R package [6]. There are 1,215 TCGA-BRCA bulk samples with cancer stages (Stage I, II, III, and IV) information.

Lung cancer data The scRNA-seq data for the lung cancer are from [12]. The original study contains five patients' scRNA-seq data and we use two lung adenocarcinoma (LUAD) patients' data with 29,888 cells from different cell types. The bulk RNA-seq data are obtained from the TCGA database via *TCGAbiolinks* (ver. 2.25.2) R package [6]. There are 576 TCGA-LUAD bulk samples with survival status and time information.

Facioscapulohumeral muscular dystrophy (FSHD) data The scRNA-seq data for FSHD are from [13]. This study contains six tissue samples, among which two are control samples the remaining four are FSHD samples, resulting in 7,047 cells. We use all the six samples as

the input scRNA-seq data. The bulk RNA-seq data are from [7]. This study contains eight control samples and 27 FSHD samples.

6 Connections of SCIPAC with decomposition/deconvolution

As shown in the main text, executing the deconvolution process is not necessary to obtain Equation (2). Even if deconvolution is performed and the composition coefficients (γ_k for $k = 1, \ldots, K$) are obtained, these coefficients do not aid in the computation of $\boldsymbol{G}^*$. The $\boldsymbol{G}^*$ computed using these coefficients will still be identical to that computed using Equation (2), which does not rely on the coefficients.

One may suggest a more straightforward approach than SCIPAC, which deconvolutes each bulk sample first and then compares the differences in proportions of each cell type between samples with different phenotypes (e.g., cancer vs. normal). However, this two-step deconvolution-based approach does not address the same problem as SCIPAC. It tests each cell type separately and independently, whereas SCIPAC (implicitly) utilizes the compositions of all cell types simultaneously to construct a classifier, regression model, or survival model. The difference between them is somewhat similar to the distinction between many single-variable linear regression models, each considering the relationship between one input variable and the outcome, versus a multi-variable linear regression model that considers the relationship between all input variables and the outcome. Thus, SCIPAC provides a more comprehensive solution.

Despite the conceptual advantages of SCIPAC, we implemented the deconvolution-based approach and applied it to our simulation data 1. We deconvoluted the bulk samples using MuSiC[14], which is one of the most popular reference-based deconvolution methods [15], to obtain the cell type proportions for each bulk sample. Subsequently, we performed a two-sample t-test on each cell type to determine whether the proportions differed between the two sample groups. The results are presented in Figure S17. It is worth noting that although this deconvolution-based strategy correctly captures the truly positive/negative-associated cells in the upper right corner of the plots, it yields an unacceptably large number of false positive discoveries. Notably, a significant proportion of cells from cell type 3 (the largest cluster of cells, which should be null) are falsely identified as significant.

This outcome is not entirely unexpected. Firstly, deconvolution methods typically tackle a constrained optimization problem, where one constraint requires the proportions of all cell

types to be non-negative, and another constraint requires that the proportions sum up to 1. These constraints are likely to introduce biases in the solutions and truncate the distributions of each proportion. Consequently, the subsequent two-sample tests are compromised. Secondly, in the deconvolution-then-test strategy, the proportions of cell types estimated in the deconvolution step have variances. However, these estimation variances are disregarded in the second step, where the proportions are treated as actual/observed values to compute the t-statistic. As a result, these t-statistics become inflated, leading to a significant number of false positive discoveries.

7 How association strength impacts the performance of SCIPAC

To examine how the strength of association impacts the performance of SCIPAC, we extended our analysis to modified simulation scheme I following these steps:

1. Generate single-cell expression using the same approach as described in the manuscript.
2. Generate 100 random numbers $a_i \sim$ Uniform$(-0.5, 0.5)$, for $i = 1, 2, \ldots, 100$.
3. Generate pseudo-bulk samples by mixing $1 - |a_i|$ proportion of cells from cell type 3 with $|a_i|$ proportion of cells from cell type 1 (when $a_i > 0$) or cell type 2 (when $a_i < 0$), where $|\cdot|$ represents the absolute value.
4. Generate the phenotype label y_i using a Bernoulli$(\exp^{a_i t}/(1+\exp^{a_i t}))$ distribution.

Within this simulation scheme, the parameter t serves as a proxy for the true association strength between the phenotype and cell types 1 & 2: for a sample with a fixed positive/negative a_i value, a larger value of t signifies a more definitive categorization as cancer/normal. We provide the estimated association strengths and p-values for different t values in Figures S18 and S19, respectively. As observed, as t increases, the estimated association becomes increasingly significant.

8 Robustness to the choice of bulk data

To examine the robustness of SCIPAC to the choice of bulk data, we divided the TCGA-PRAD bulk data used in our paper into two separate parts based on their collection years. Each

part was treated as an independent (bulk) RNA-seq dataset. Specifically, the bulk samples were collected between the years 2000 and 2013. We allocated the samples collected in the last three years to part 1, and samples collected in other years to part 2. By doing so, we ensured that both parts contained a similar number of samples.

The association strengths estimated by SCIPAC on these two divided parts are presented in Figure S20 a and c, respectively, while the corresponding p-values are shown in Figure S20 b and d. Despite some minor discrepancies, we observed that both the estimated association strengths and p-values were largely similar between the two parts. This indicates that SCIPAC demonstrates reasonable robustness when it comes to the selection of bulk samples.

9 The choice of α

The elastic net model has two tuning parameters, λ and α. λ controls the overall penalty strength, while α determines the mixing proportions of the ℓ_1 and ℓ_2 penalties. Tuning these two parameters can be time-consuming, and people have found that the choice of λ is typically more critical than that of α. Consequently, we opted to fix the α value (although the SCIPAC R package allows users to use non-default α values) and only tune λ. Through our experiments, we found that $\alpha = 0.4$ performed well across all scenarios. We have also found that SCIPAC demonstrated limited sensitivity to the choice of α. Figure S21 illustrates the results of SCIPAC under three different α values: 0.3, 0.4, and 0.5, which agree well with each other.

10 More discussions regarding the prostate cancer dataset—relationship between being cancer-specific and cancer-associated

Many studies [16, 17] have suggested that the B cell infiltration and the formation of Tertiary lymphoid structures (TLS) could be cancer-specific, but this does not have to contradict with SCIPAC's inference, since being cancer-specific does not necessarily imply a cell type is positively cancer-associated. In the context of SCIPAC, a positive association of a cell type means that increasing this cell type will lead to increasing the probability of the presence of a phenotype. Mathematically, increasing a cancer-specific cell type may dilute the concentrations of cell types that have even stronger associations with the phenotype, and hence decrease the probability of the presence of cancer. Biologically, cell types found exclusively

in cancer tissues encompass both cells that initiate or promote cancer development and cells employed by the immune system to combat cancer. Thus, cancer-specific cell types can exhibit either positive or negative associations with cancer, according to SCIPAC's definition of phenotype association. In fact, accumulating research has indicated that there is a close association between tumor-associated TLSs and better prognosis, as well as favorable clinical outcomes following immunotherapy [16, 17, 18], which align with our analysis.

11 Discussions on the calculation of the p-value

Our computation of the p-value assumes that the bootstrapped p-values follow a Gaussian distribution. To examine whether this assumption holds in real data, we plot histograms of the bootstrapped Λ_k's for each cluster in our first real dataset, the prostate cancer dataset. These histograms are presented in Fig. S22. Further, we test whether the bootstrapped Λ_k's for each cluster are normally distributed using the Shapiro-Wilk's (S-W) test, and we provide the corresponding p-values in the subplot titles. After multiple-test correction using Benjamini & Hochberg's false discovery rate (FDR) [19], none of the 27 clusters have FDR < 0.05. This suggests that the normal assumption is not significantly violated in this real dataset.

Our p-value calculation lacks size correction. Applying size correction in our case is challenging due to the strong correlation among the p-values from different clusters. Standard techniques for p-value correction, such as Benjamini & Hochberg's FDR, do not yield accurate results in this scenario and tend to be overly conservative. Consequently, we made the decision not to apply size correction and instead report the uncorrected p-values. In practice, this approach has not yielded excessively liberal results. For example, in our simulation data 1, even with a high-resolution 3.5 that generates 47 clusters, which corresponds to 47 tests, the uncorrected p-values do not result in a significant number of false positives, as demonstrated in Figure 1d and f in the main text. In summary, the uncorrected p-value is theoretically imperfect, but based on empirical evidence, it performs adequately and is a preferable choice compared to improperly corrected p-values using methods such as Benjamini & Hochberg's FDR.

12 Exploring the Use of Metacells in SCIPAC

I used metacells implemented in the SEACells Python package, as cited in [20] and available on its GitHub repository (https://github.com/dpeerlab/SEACells).

Metacells are distinct and homogeneous groups of cells [20, 21, 22, 23]. They serve to simplify large single-cell datasets by representing the original data with a reduced number of metacells. We detail below how we integrate metacells into our SCIPAC algorithm.

As outlined in the "Results" section of our main text, SCIPAC involves four steps. The initial step uses the Louvain algorithm from the Seurat package to cluster cells and employs cluster centroids as their representatives. These centroids are then inputted into the subsequent steps (2 to 4) of SCIPAC for computing association strength and assessing statistical significance.

For incorporating metacells into SCIPAC, we propose two approaches. The first approach treats each metacell as a representative of a cell cluster, essentially replacing SCIPAC's first step with SEACells. Hence, metacells become the input for SCIPAC's steps 2 to 4. The second approach considers metacells as a condensed single-cell dataset, which is then clustered using SCIPAC's Louvain algorithm. Here, metacells are inputted into the entire SCIPAC process, from steps 1 to 4.

12.1 Results from the first strategy

In our study, we consider Simulation dataset 1, which comprises three cell types: one positively associated, one negatively associated, and one not associated. We used SEACells on this dataset to create three metacells. These metacells are treated as representatives of their corresponding cell groups and are integrated into steps 2 to 4 of the SCIPAC process. The results are shown in Figure S23.

Surprisingly, no cells were identified as significantly associated with the phenotype. This raises the question: Why did the SEACells+SCIPAC combination fail? To investigate, we examined how the metacells grouped cells.

Figure S24 displays cells categorized into distinct metacells, each represented by a unique color. The centroids of these metacells are labeled as 1, 2, and 3. Metacells 1 and 3 predominantly consist of the null cell type, whereas metacell 2 is a composite of the positively associated, negatively associated, and a fraction of the null cell types. Consequently, none of these metacells should be linked to the phenotype, corroborating the accurate inference by our SCIPAC algorithm. The ineffectiveness of the SEACells+SCIPAC combination is attributed to SEACells' erroneous cell grouping.

We can quantitatively measure the agreement of cell assignments (using either Louvain clustering or metacells) to the true cell labels using the Adjusted Rand Index (ARI), which is a commonly used measure of similarity between two data clusterings. ARI ranges from -1 to 1, where 1 indicates perfect agreement, 0 no agreement, and -1 complete disparity. The

ARI for Louvain clustering compared to the true clustering is 0.9951, while for metacells it is only 0.0320.

Could increasing the number of metacells enhance the SEACells+SCIPAC combination's effectiveness? Possibly, as a greater number of metacells increases the chances of positively and negatively associated cells being segregated into different metacells, decreasing their mixture with other cell types.

Initially, we raised the number of metacells to five. The outcomes are depicted in Figure S25, and the cell distribution by metacells is illustrated in Figure S26. This led to successful identification of many positively associated cells, primarily because metacell 5, to which they are assigned, contains few cells of other types. Conversely, negatively associated cells remain unidentified. They are mainly grouped into metacell 3, which has a significant number of null cells and a substantial fraction of positively associated cells. The centroid of metacell 3 aligns more closely with the null cell type than the negatively associated cell type, suggesting its overall association is nearly null. Thus, the subpar performance of the SEACells+SCIPAC combination can still be ascribed to the inefficiencies of SEACells.

Subsequently, we expanded the number of metacells to 20. The results of this increase are displayed in Figure S27, with the cell grouping within these metacells detailed in Figure S28. Following this adjustment, a small fraction of negatively associated cells is now accurately identified. This improvement is attributed to their assignment to metacell 4, which contains few cells from other cell types. However, the majority of negatively associated cells continue to be unrecognized. This is because they are predominantly grouped into metacells that include a high proportion of cells from other types.

Unlike SEACells/metacells, which struggles with effectively grouping cells, the Louvain algorithm employed by SCIPAC demonstrates superior performance in this task. Figure S29 shows how the Louvain algorithm clustered the cells into 3, 5, and 20 clusters. The corresponding centroids are also shown. In the 3-cluster result, each cluster closely matched a cell type. In the 5-cluster result, three larger clusters (clusters 1, 2, and 3) closely aligned with the original three cell types, while the remaining two clusters contained a small number of cells. In the 20-cluster results, we found that the cell clusters corresponding to the negatively/positively associated cells typically did not contain cells from the other two types.

Finally, we checked whether the reason for the failure of the SEACells+SCIPAC combination—the metacells being inferior clusters of cells—applies to real data. Figure S30 shows cells colored according to true cell types (subfigure a, 13 cell types), cells colored according to metacells given by SEACells (subfigure b, 13 metacells), and cells colored according to clusters given by the Louvain algorithm (subfigure, 13 clusters). The centroids are also

illustrated.

It is clear that the clusters given by the Louvain algorithm are closer to the true cell types than those given by SEACells, which show much more mixing. Again, we used ARI to quantitatively measure the agreements. The ARI between the cell clusters given by the Louvain algorithm is 0.4914, while the ARI for the metacell-based clusters is only 0.2883.

12.2 Results from the second strategy

In this strategy, we use metacells to reduce the size of the original single-cell data, and then use these metacells as input for the SCIPAC algorithm. The default parameter in SEACells generates 130 metacells for our simulation dataset 1, which consists of 10,000 cells. These metacells are further clustered by the Louvain algorithm in the first step of SCIPAC, and the number of metacell clusters depends on the resolution ("Res") parameter in the Louvain algorithm.

Figure S31 presents the ground truth of how these metacells are associated with the phenotype. In the plot, each metacell is colored according to the majority cell type of the cells it contains. This majority cell type also reflects how each metacell should be associated with the phenotype, as the three cell types have different associations with the phenotype: one positive, one negative, and one neutral. Out of the 130 metacells, 22 predominantly contain cells that are either positively or negatively associated with the phenotype. All of these metacells are located in the top-right corner of the plot.

In Figure S32a and Figure S33a, the results for estimated association strengths and p-values are shown, respectively, at a resolution of 1.0, which groups the 130 metacells into two clusters. At this resolution, no cells are identified as significantly associated.

Figures S32b & c and S33b & c show the outcomes at Res = 1.1 and 1.2, yielding four and seven clusters, respectively. However, these settings also fail to yield any significant findings.

Increasing the resolution further to 1.4, we observe a substantial increase in clusters, totaling 21, as shown in Figure S32d and Figure S33d. Despite this increase, the results still do not reveal any significant associations.

Do these poor results still stem from the inadequate performance of metacells in grouping cells? To explore this, we examine how the Louvain algorithm groups these 130 metacells into clusters at different resolutions, as shown in Figure S34.

Remarkably, in all scenarios, including at a resolution of 1.4 with 21 clusters, all of the 22 metacells that mainly contain cells (either positively or negatively) associated with the phenotype, which are located in the top-right corner of each subplot, consistently fall into a

single cluster. This cluster, because it encompasses all of the 22 metacells, contains a nearly equal mix of positively and negatively associated cells. Therefore, this cluster should not be linked to the phenotype. SCIPAC's analysis supports this conclusion, correctly inferring the non-association of this cluster with the phenotype.

We may delve further and question why the Louvain algorithm consistently groups all these 22 metacells into a single cluster. This is particularly puzzling given that the Louvain algorithm effectively clustered the original single cells, separating positively and negatively associated cells into different clusters even when $K = 3$, as detailed in our manuscript. Is this due to those metacells each containing both positively and negatively associated cells, making these metacells not very distinct from each other? Upon examining the cell type labels within each metacell, we confirm this to be the case. Among the 22 metacells where the majority of cells are associated with the phenotype, only three (13.6%) are not a mix of both positively and negatively associated cells. In 11 of these 22 metacells (50.0%), the mix ratio is at least 10:90. In 4 of these 22 metacells (18.2%), the mix ratio is at least 40:60. Clearly, metacells performed poorly in grouping cells according to their cell type, and thus, by extension, their function.

Quick summary

Our comparative analysis reveals that combining SCIPAC with SEACells results in significantly reduced performance compared to using SCIPAC directly on original single-cell data. The primary reason for this appears to be the subpar performance of SEACells/metacells in cell grouping, especially when contrasted with the Louvain algorithm. This issue is attributed to SEACells/metacells, not to any inherent problem with our SCIPAC algorithm. Given these findings, we currently advise against using metacells for SCIPAC applications. We have revised our manuscript to reflect these insights, and have added our comprehensive analysis, as discussed above, to the Supplementary Material.

On a positive note, SCIPAC, along with the embedded Louvain algorithm, demonstrates sufficient computational efficiency and manageable memory usage even with large single-cell datasets. This eliminates the need for metacells to reduce data size. To demonstrate this, we conducted an experiment using the prostate cancer dataset, artificially expanding the sample size from 8,657 to 200,000 cells. This was achieved by oversampling pre-processed data with replacement and adding random Gaussian noise. SCIPAC was then run on this data using a MacBook Pro with an Apple M1 chip, 8 cores, and 16GB RAM. The initial clustering step using the Louvain algorithm took approximately 46 minutes, while the subsequent SCIPAC processes were completed in under two minutes. The peak memory usage

was only 2.5 GB.

References

[1] Luke Zappia, Belinda Phipson, and Alicia Oshlack. Splatter: simulation of single-cell rna sequencing data. *Genome biology*, 18(1):1–15, 2017.

[2] Tim Stuart, Andrew Butler, Paul Hoffman, Christoph Hafemeister, Efthymia Papalexi, William M Mauck, Yuhan Hao, Marlon Stoeckius, Peter Smibert, and Rahul Satija. Comprehensive integration of single-cell data. *Cell*, 177(7):1888–1902, 2019.

[3] Leland McInnes, John Healy, and James Melville. Umap: Uniform manifold approximation and projection for dimension reduction. *arXiv preprint arXiv:1802.03426*, 2018.

[4] Gennady Korotkevich, Vladimir Sukhov, Nikolay Budin, Boris Shpak, Maxim N Artyomov, and Alexey Sergushichev. Fast gene set enrichment analysis. *BioRxiv*, page 060012, 2021.

[5] Dharmesh D. Bhuva, Gordon K. Smyth, and Alexandra Garnham. *msigdb: An ExperimentHub Package for the Molecular Signatures Database (MSigDB)*, 2021. R package version 1.2.0.

[6] Antonio Colaprico, Tiago C Silva, Catharina Olsen, Luciano Garofano, Claudia Cava, Davide Garolini, Thais S Sabedot, Tathiane M Malta, Stefano M Pagnotta, Isabella Castiglioni, et al. Tcgabiolinks: an r/bioconductor package for integrative analysis of tcga data. *Nucleic acids research*, 44(8):e71–e71, 2016.

[7] Chao-Jen Wong, Leo H Wang, Seth D Friedman, Dennis Shaw, Amy E Campbell, Chris B Budech, Leann M Lewis, Richard JFL Lemmers, Jeffrey M Statland, Silvère M van der Maarel, et al. Longitudinal measures of rna expression and disease activity in fshd muscle biopsies. *Human molecular genetics*, 29(6):1030–1043, 2020.

[8] Kevin Blighe, Sharmila Rana, and Myles Lewis. Enhancedvolcano: Publication-ready volcano plots with enhanced colouring and labeling. *R package version*, 1(0), 2019.

[9] Duanchen Sun, Xiangnan Guan, Amy E Moran, Ling-Yun Wu, David Z Qian, Pepper Schedin, Mu-Shui Dai, Alexey V Danilov, Joshi J Alumkal, Andrew C Adey, et al.

Identifying phenotype-associated subpopulations by integrating bulk and single-cell sequencing data. *Nature biotechnology*, 40(4):527–538, 2022.

[10] Zewen Kelvin Tuong, Kevin W Loudon, Brendan Berry, Nathan Richoz, Julia Jones, Xiao Tan, Quan Nguyen, Anne George, Satoshi Hori, Sarah Field, et al. Resolving the immune landscape of human prostate at a single-cell level in health and cancer. *Cell Reports*, 37(12), 2021.

[11] Sunny Z Wu, Ghamdan Al-Eryani, Daniel Lee Roden, Simon Junankar, Kate Harvey, Alma Andersson, Aatish Thennavan, Chenfei Wang, James R Torpy, Nenad Bartonicek, et al. A single-cell and spatially resolved atlas of human breast cancers. *Nature genetics*, 53(9):1334–1347, 2021.

[12] Diether Lambrechts, Els Wauters, Bram Boeckx, Sara Aibar, David Nittner, Oliver Burton, Ayse Bassez, Herbert Decaluwé, Andreas Pircher, Kathleen Van den Eynde, et al. Phenotype molding of stromal cells in the lung tumor microenvironment. *Nature medicine*, 24(8):1277–1289, 2018.

[13] Anita van den Heuvel, Ahmed Mahfouz, Susan L Kloet, Judit Balog, Baziel GM van Engelen, Rabi Tawil, Stephen J Tapscott, and Silvère M van der Maarel. Single-cell rna sequencing in facioscapulohumeral muscular dystrophy disease etiology and development. *Human molecular genetics*, 28(7):1064–1075, 2019.

[14] Xuran Wang, Jihwan Park, Katalin Susztak, Nancy R Zhang, and Mingyao Li. Bulk tissue cell type deconvolution with multi-subject single-cell expression reference. *Nature communications*, 10(1):380, 2019.

[15] Haijing Jin and Zhandong Liu. A benchmark for rna-seq deconvolution analysis under dynamic testing environments. *Genome biology*, 22:1–23, 2021.

[16] Ton N Schumacher and Daniela S Thommen. Tertiary lymphoid structures in cancer. *Science*, 375(6576):eabf9419, 2022.

[17] Wendi Kang, Zhichao Feng, Jianwei Luo, Zhenhu He, Jun Liu, Jianzhen Wu, and Pengfei Rong. Tertiary lymphoid structures in cancer: The double-edged sword role in antitumor immunity and potential therapeutic induction strategies. *Frontiers in immunology*, 12:689270, 2021.

[18] Zhan Zhao, Hui Ding, Zheng-bin Lin, Sheng-hui Qiu, Yi-ran Zhang, Yan-guan Guo, Xiao-dong Chu, Loi I Sam, Jing-hua Pan, and Yun-long Pan. Relationship between

tertiary lymphoid structure and the prognosis and clinicopathologic characteristics in solid tumors. *International Journal of Medical Sciences*, 18(11):2327, 2021.

[19] Yoav Benjamini and Yosef Hochberg. Controlling the false discovery rate: a practical and powerful approach to multiple testing. *Journal of the Royal statistical society: series B (Methodological)*, 57(1):289–300, 1995.

[20] Sitara Persad, Zi-Ning Choo, Christine Dien, Noor Sohail, Ignas Masilionis, Ronan Chaligné, Tal Nawy, Chrysothemis C Brown, Roshan Sharma, Itsik Pe'er, et al. Seacells infers transcriptional and epigenomic cellular states from single-cell genomics data. *Nature Biotechnology*, pages 1–12, 2023.

[21] Yael Baran, Akhiad Bercovich, Arnau Sebe-Pedros, Yaniv Lubling, Amir Giladi, Elad Chomsky, Zohar Meir, Michael Hoichman, Aviezer Lifshitz, and Amos Tanay. Metacell: analysis of single-cell rna-seq data using k-nn graph partitions. *Genome biology*, 20(1):1–19, 2019.

[22] Oren Ben-Kiki, Akhiad Bercovich, Aviezer Lifshitz, and Amos Tanay. Metacell-2: a divide-and-conquer metacell algorithm for scalable scrna-seq analysis. *Genome biology*, 23(1):1–18, 2022.

[23] Mariia Bilous, Loc Tran, Chiara Cianciaruso, Aurélie Gabriel, Hugo Michel, Santiago J Carmona, Mikael J Pittet, and David Gfeller. Metacells untangle large and complex single-cell transcriptome networks. *BMC bioinformatics*, 23(1):336, 2022.

Supplementary tables

Table S1: Log odds ratio for each cell type on the prostate cancer data

	SCIPAC		Scissor $\alpha = 0.01$		Scissor $\alpha = 0.05$		Scissor $\alpha = 0.10$	
	ρ_+	ρ_-	ρ_+	ρ_-	ρ_+	ρ_-	ρ_+	ρ_-
BE	-4.0564	1.1675	-2.6568	2.5287	-2.2179	1.9976	-2.533	1.8683
HE	-Inf	2.06	-4.6335	3.4902	-3.7736	2.7619	-3.3803	2.6185
CE	-3.5231	0.9539	-0.5748	0.3551	-0.5334	0.2348	-0.6382	0.2162
LE-KLK3	0.0479	0.1019	-0.2112	0.9292	0.0849	0.8482	0.1039	0.7851
LE-KLK4	1.349	-0.3655	0.559	-0.6871	0.2528	-1.0613	0.2293	-1.0984
Endothelial	-Inf	-Inf	-0.4108	-1.9342	-0.743	-1.4059	-0.6799	-1.3037
MNP	-Inf	4.2997	3.5965	-Inf	2.1398	-Inf	1.8219	-Inf
NK cell	-Inf	-Inf	0.432	-Inf	0.2395	-Inf	0.2251	-Inf
T cell	-Inf	-4.3215	-0.4809	-3.2746	-0.7756	-3.2432	-0.8428	-3.205
B cell	-Inf	3.0356	-0.6692	-3.3585	-0.8496	-Inf	-0.6788	-Inf

Table S2: Log odds ratio for each cell type on the prostate cancer data (Cont'd).

	SCIPAC		Scissor $\alpha = 0.15$		Scissor $\alpha = 0.20$		Scissor $\alpha = 0.25$	
	ρ_+	ρ_-	ρ_+	ρ_-	ρ_+	ρ_-	ρ_+	ρ_-
BE	-4.0564	1.1675	-2.2687	1.7723	-2.0712	1.7124	-1.9079	1.6852
HE	-Inf	2.06	-3.116	2.5236	-2.9185	2.5302	-Inf	2.544
CE	-3.5231	0.9539	-0.5965	0.2776	-0.5986	0.2139	-0.5366	0.0881
LE-KLK3	0.0479	0.1019	0.1432	0.7402	0.1344	0.7258	0.1624	0.7128
LE-KLK4	1.349	-0.3655	0.2123	-1.0927	0.1641	-1.1057	0.0833	-1.0894
Endothelial	-Inf	-Inf	-0.8167	-1.1072	-0.7309	-1.1489	-0.6918	-1.4592
MNP	-Inf	4.2997	1.7557	-Inf	1.8078	-Inf	1.8524	-Inf
NK cell	-Inf	-Inf	0.1863	-Inf	0.0708	-Inf	0.2341	-Inf
T cell	-Inf	-4.3215	-0.9836	-3.3463	-0.9457	-4.7708	-0.9881	-4.6018
B cell	-Inf	3.0356	-1.2777	-Inf	-1.378	-Inf	-1.6302	-Inf

Table S3: Agreement of the results by SCIPAC and Scissor on the prostate cancer data

	SCIPAC	Scissor	Agreement
BE	Negative	Negative	Agree
HE	Negative	Negative	Agree
CE	Inconclusive	Inconclusive	Agree
LE-KLK3	Inconclusive	Inconclusive	Agree
LE-KLK4	Positive	Inconclusive	Disagree
Endothelial	Inconclusive	Inconclusive	Agree
MNP	Negative	Positive	Disagree
NK cell	Inconclusive	Inconclusive	Agree
T cell	Inconclusive	Inconclusive	Agree
B cell	Negative	Inconclusive	Disagree

Supplementary figures

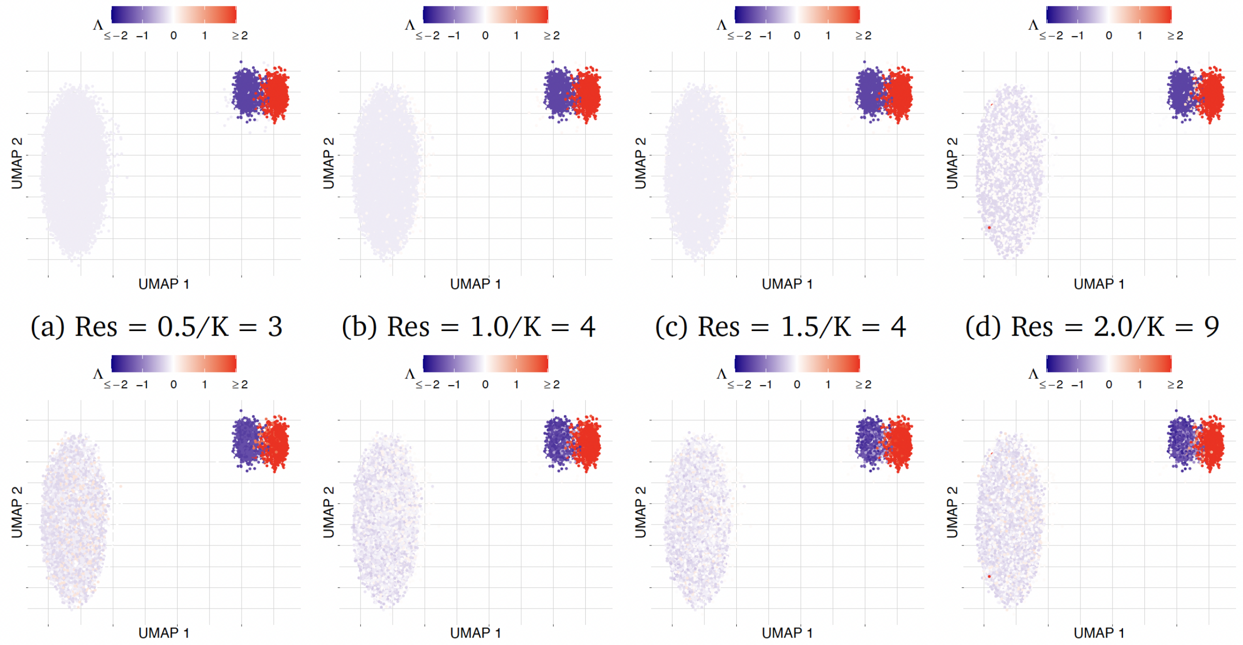

(a) Res = 0.5/K = 3 (b) Res = 1.0/K = 4 (c) Res = 1.5/K = 4 (d) Res = 2.0/K = 9

(e) Res = 2.5/K = 20 (f) Res = 3.0/K = 34 (g) Res = 3.5/K = 47 (h) Res = 4.0/K = 61

Fig S1. UMAP visualization of the estimated association strengths Λ given by SCIPAC under different resolutions on simulated data under scheme I. Res stands for resolution, and K stands for the number of cell clusters given by this resolution.

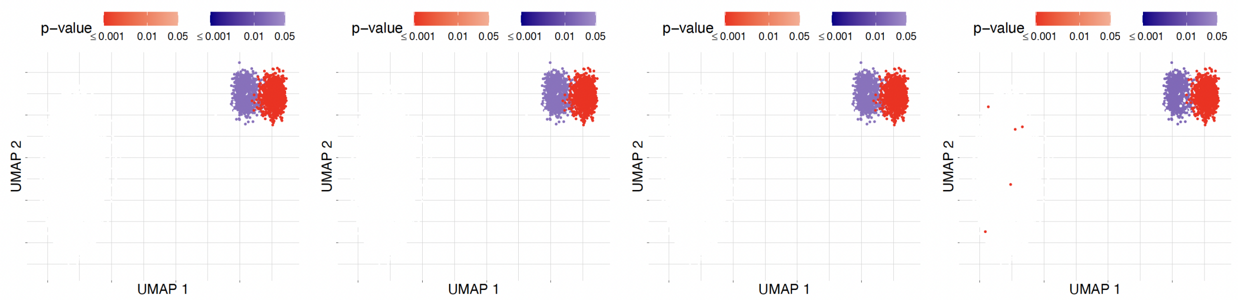

(a) Res = 0.5/K = 3 (b) Res = 1.0/K = 4 (c) Res = 1.5/K = 4 (d) Res = 2.0/K = 9

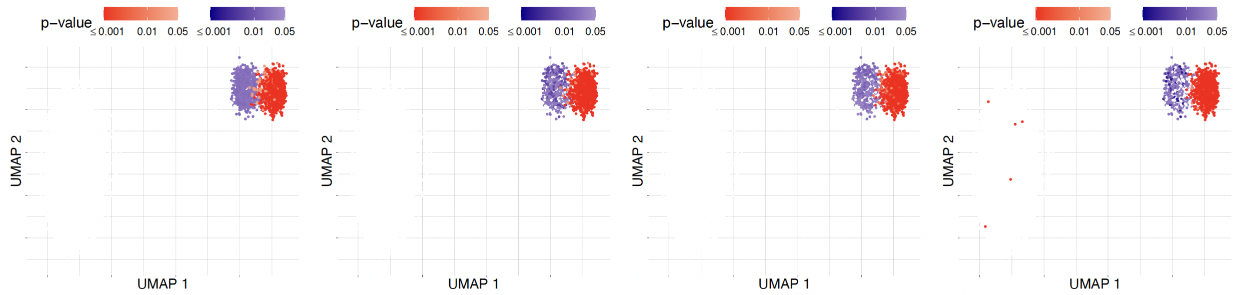

Fig S2. UMAP visualization of the p-values given by SCIPAC under different resolutions on simulated data under scheme I. Res stands for resolution, and K stands for the number of cell clusters given by this resolution.

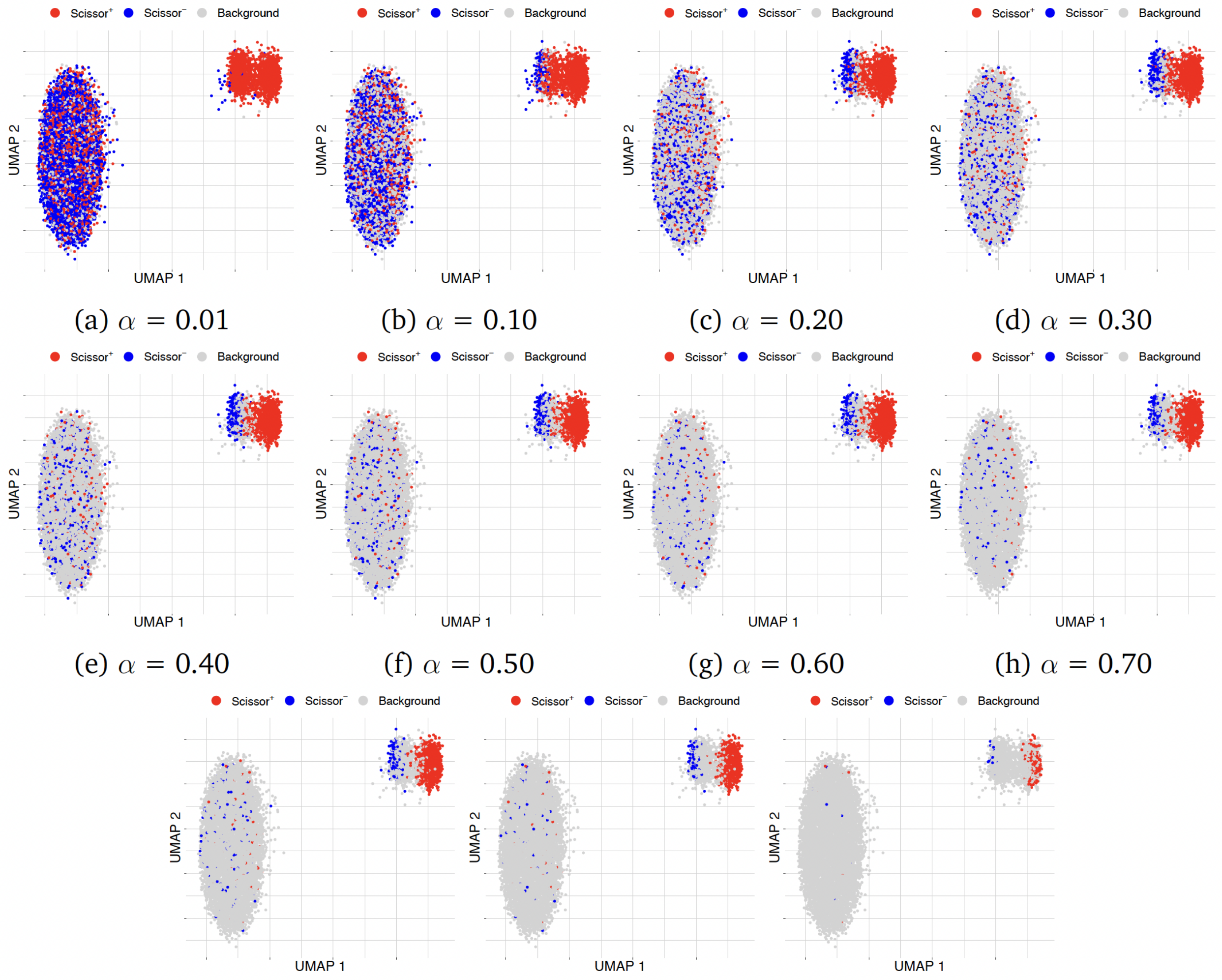

(a) α = 0.01 (b) α = 0.10 (c) α = 0.20 (d) α = 0.30

(e) α = 0.40 (f) α = 0.50 (g) α = 0.60 (h) α = 0.70

(i) α = 0.80 (j) α = 0.90 (k) α = 0.99

Fig S3. UMAP visualization of results given by Scissor under different α values on simulated data under scheme I.

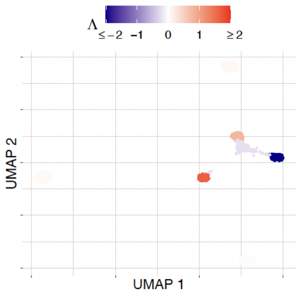

(a) Res = 0.5/K = 7

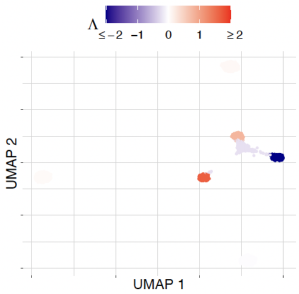

(b) Res = 1.0/K = 7

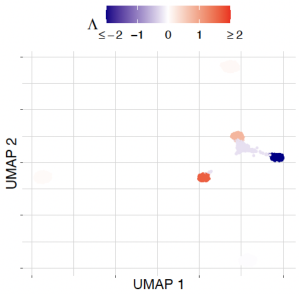

(c) Res = 1.5/K = 7

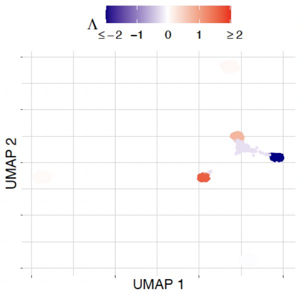

(d) Res = 2.0/K = 7

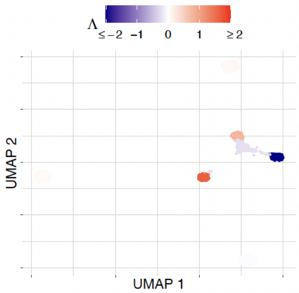

(e) Res = 2.5/K = 7

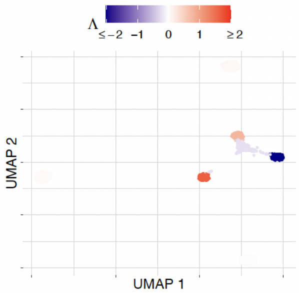

(f) Res = 3.0/K = 7

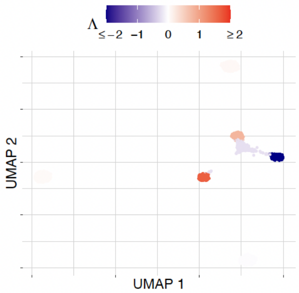

(g) Res = 3.5/K = 7

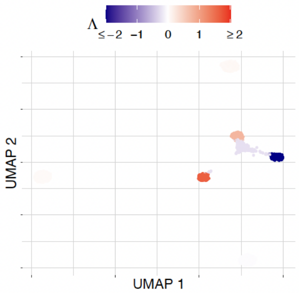

(h) Res = 4.0/K = 9

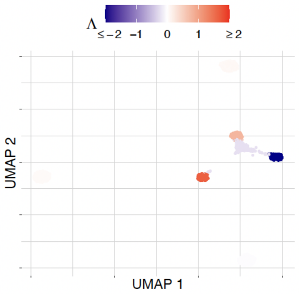

(i) Res = 4.5/K = 13

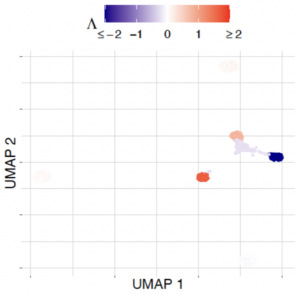

(j) Res = 5.0/K = 18

Fig S4. UMAP visualization of the estimated association strengths Λ given by SCIPAC under different resolutions on simulated data under scheme II. Res stands for resolution, and K stands for the number of cell clusters given by this resolution.

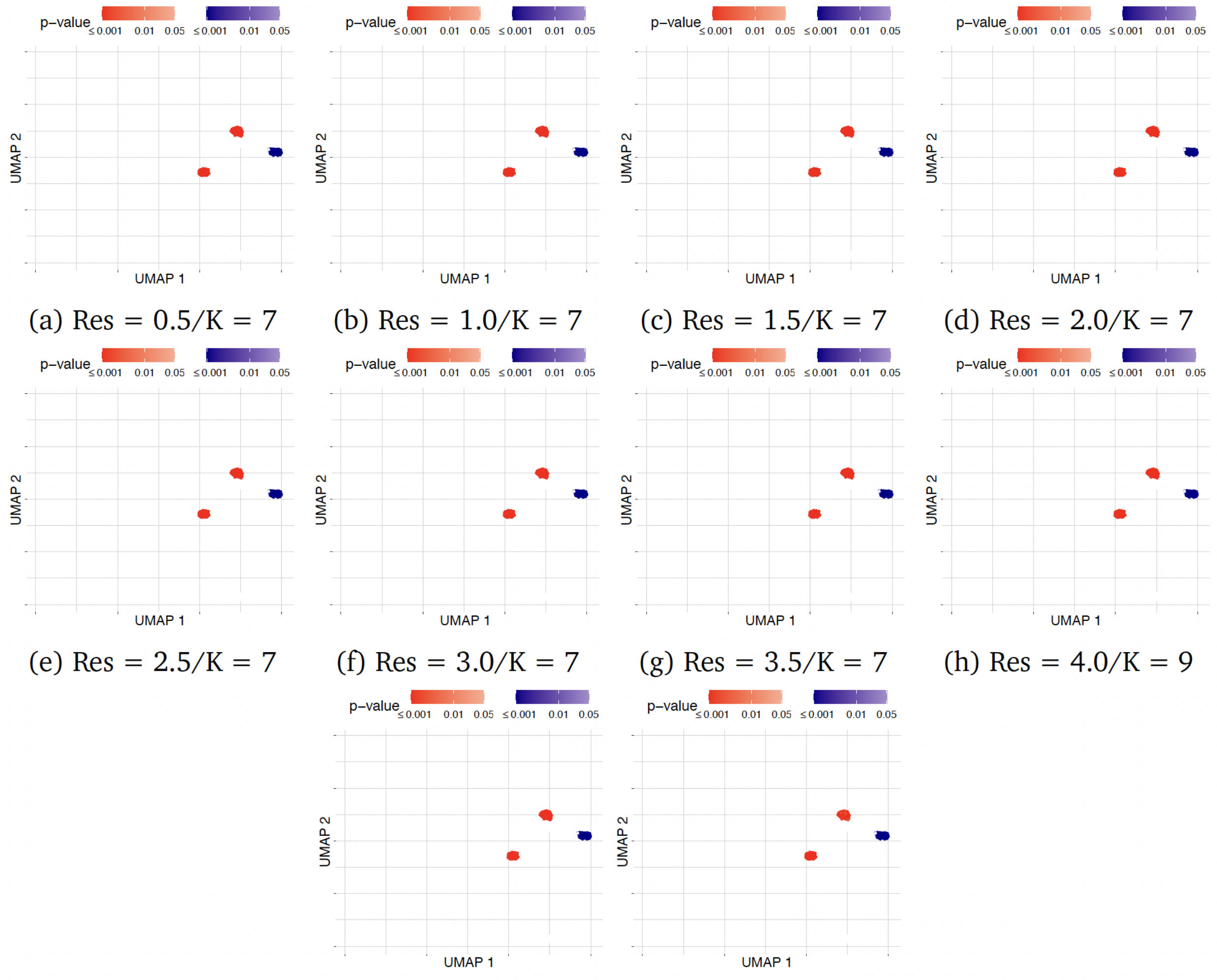

(a) Res = 0.5/K = 7 (b) Res = 1.0/K = 7 (c) Res = 1.5/K = 7 (d) Res = 2.0/K = 7

(e) Res = 2.5/K = 7 (f) Res = 3.0/K = 7 (g) Res = 3.5/K = 7 (h) Res = 4.0/K = 9

(i) Res = 4.5/K = 13 (j) Res = 5.0/K = 18

Fig S5. UMAP visualization of the p-values given by SCIPAC under different resolutions on simulated data under scheme II. Res stands for resolution, and K stands for the number of cell clusters given by this resolution.

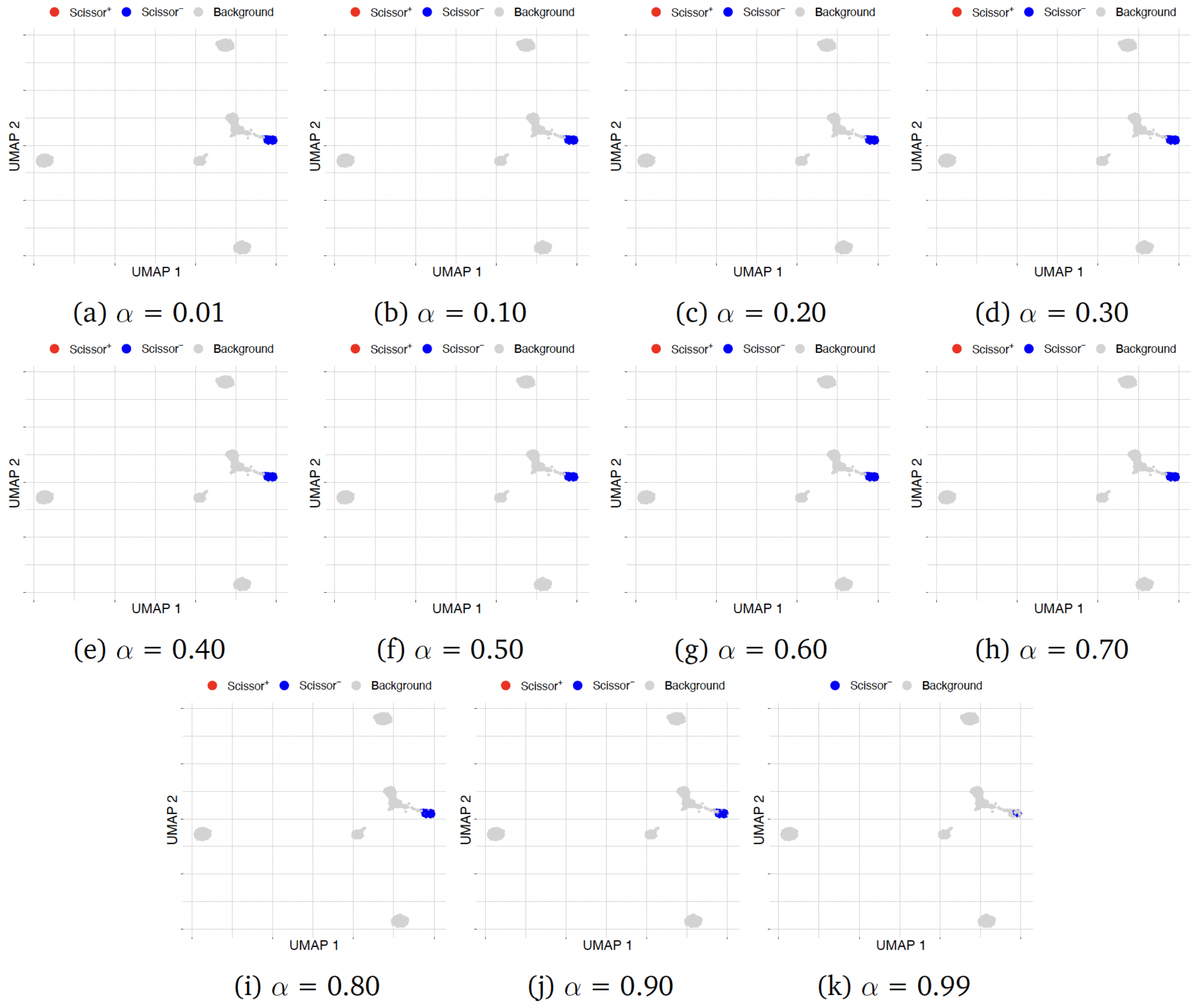

(a) $\alpha = 0.01$ (b) $\alpha = 0.10$ (c) $\alpha = 0.20$ (d) $\alpha = 0.30$

(e) $\alpha = 0.40$ (f) $\alpha = 0.50$ (g) $\alpha = 0.60$ (h) $\alpha = 0.70$

(i) $\alpha = 0.80$ (j) $\alpha = 0.90$ (k) $\alpha = 0.99$

Fig S6. UMAP visualization of results given by Scissor under different α values on simulated data under scheme II.

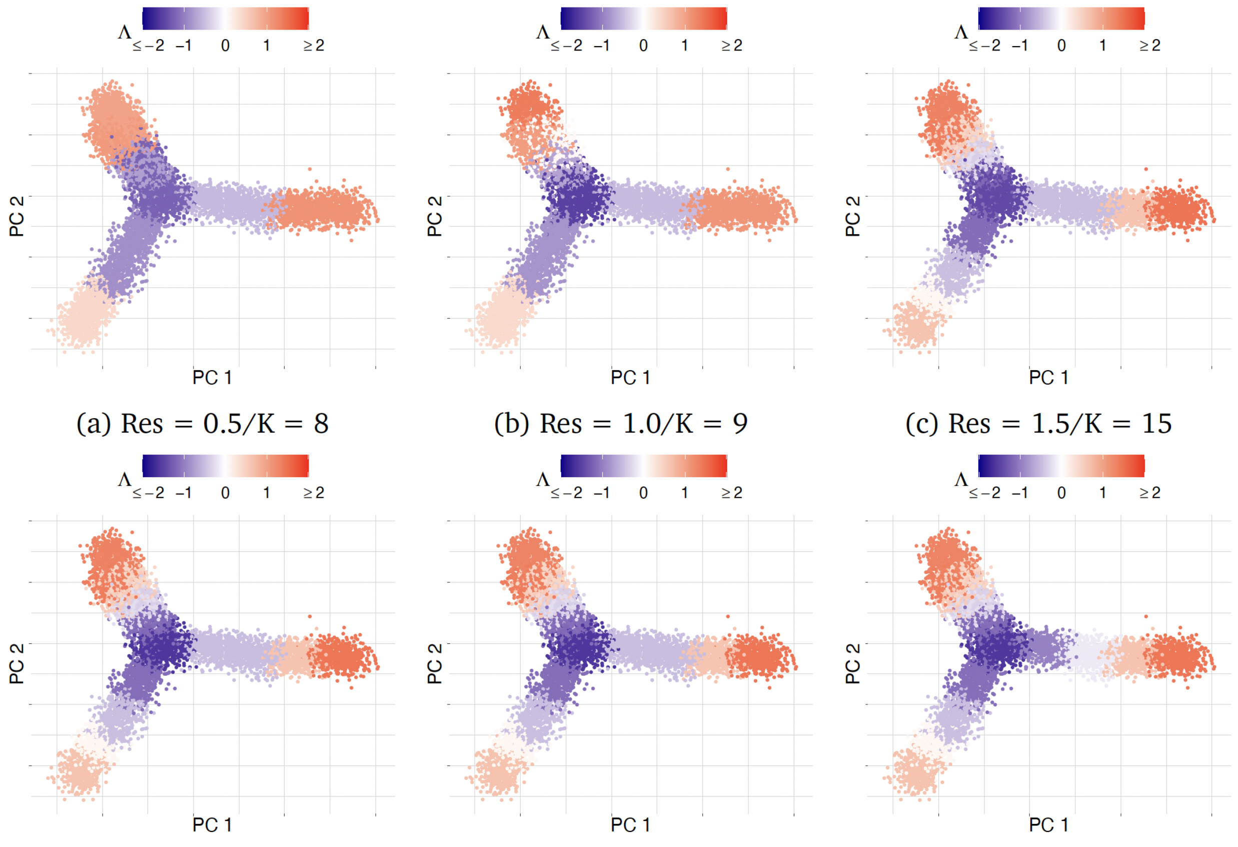

(a) Res = 0.5/K = 8 (b) Res = 1.0/K = 9 (c) Res = 1.5/K = 15

(d) Res = 2.0/K = 16 (e) Res = 2.5/K = 16 (f) Res = 3.0/K = 17

Fig S7. UMAP visualization of the estimated association strengths Λ given by SCIPAC under different resolutions on simulated data under scheme III. Res stands for resolution, and K stands for the number of cell clusters given by this resolution.

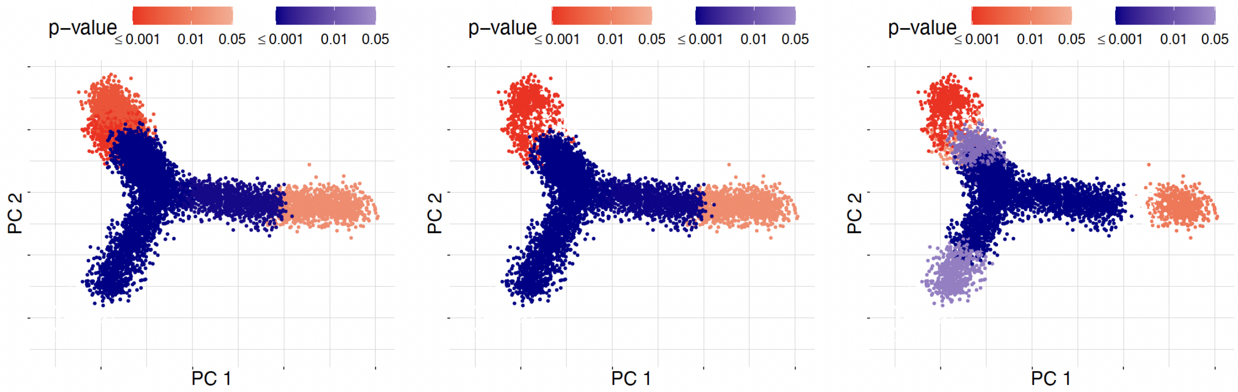

(a) Res = 0.5/K = 8 (b) Res = 1.0/K = 9 (c) Res = 1.5/K = 15

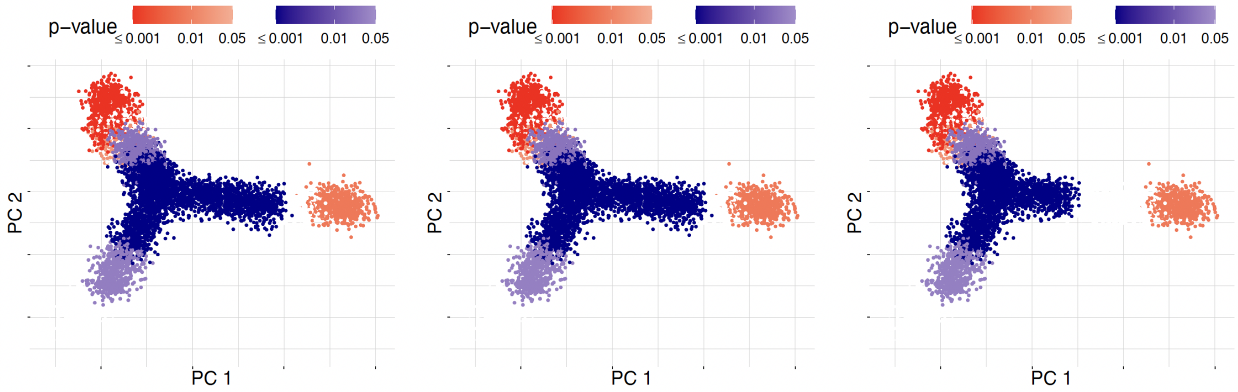

(d) Res = 2.0/K = 16 (e) Res = 2.5/K = 16 (f) Res = 3.0/K = 17

Fig S8. UMAP visualization of the p-values given by SCIPAC under different resolutions on simulated data under scheme III. Res stands for resolution, and K stands for the number of cell clusters given by this resolution.

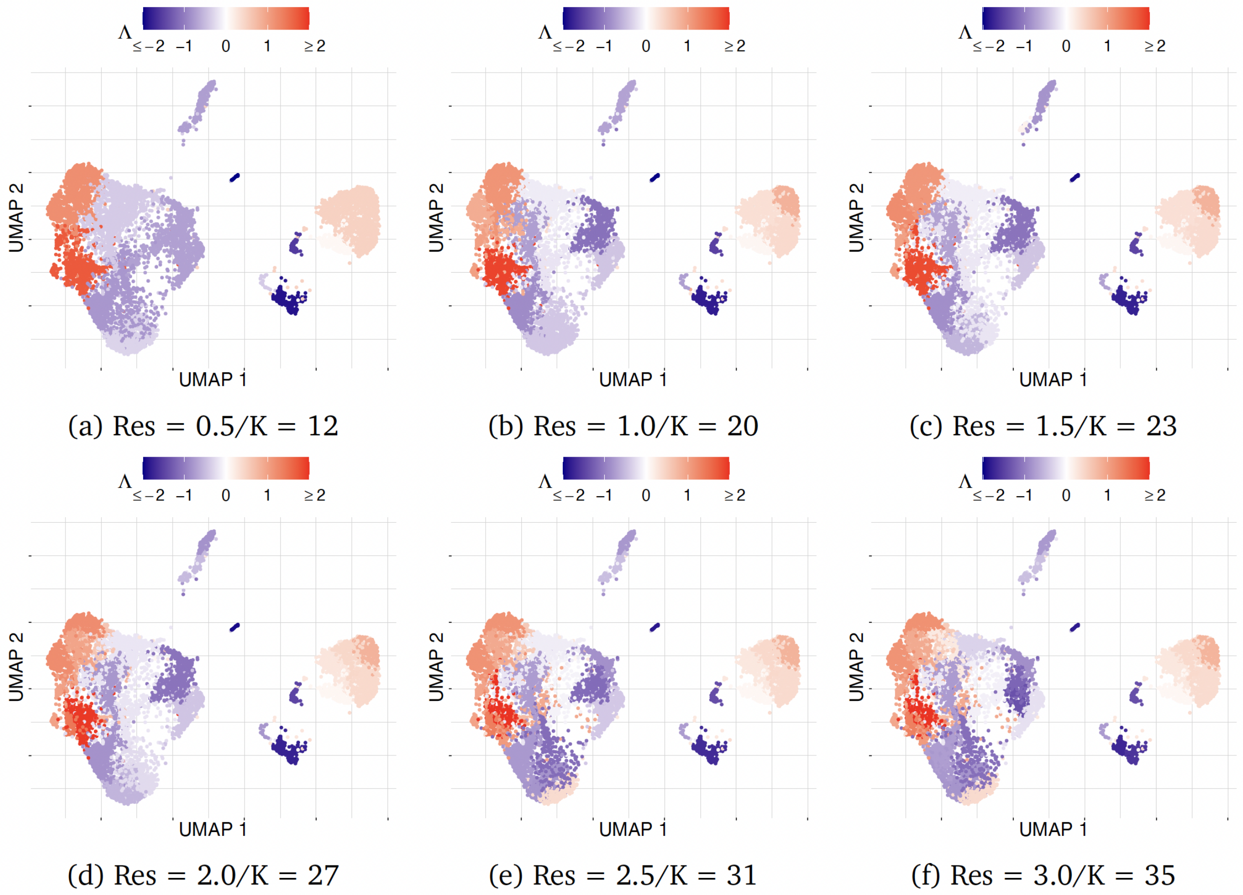

(a) Res = 0.5/K = 12

(b) Res = 1.0/K = 20

(c) Res = 1.5/K = 23

(d) Res = 2.0/K = 27

(e) Res = 2.5/K = 31

(f) Res = 3.0/K = 35

Fig S9. UMAP visualization of the estimated association strengths Λ given by SCIPAC under different resolutions on the prostate cancer data. Res stands for resolution, and K stands for the number of cell clusters given by this resolution.

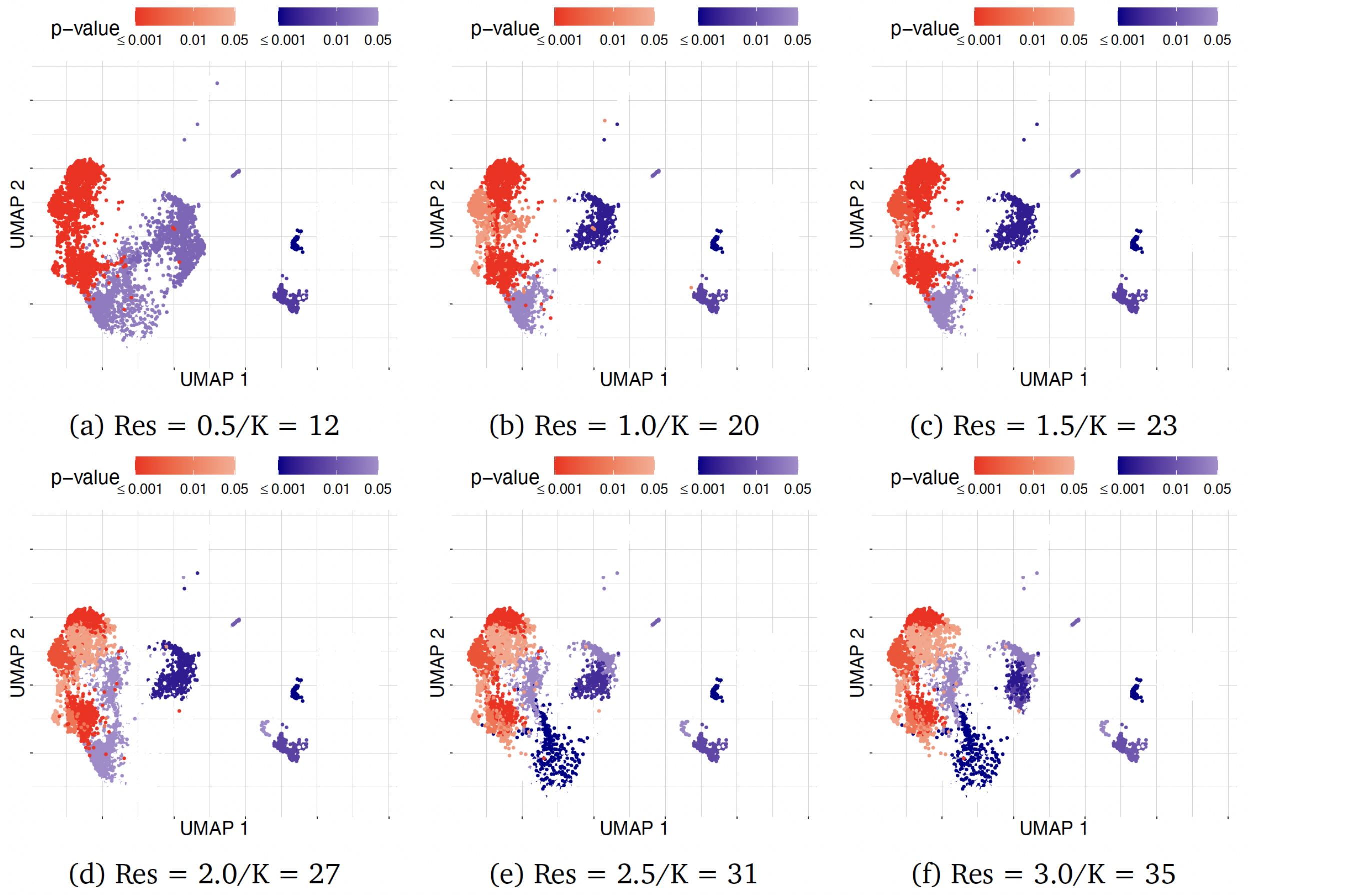

(a) Res = 0.5/K = 12 (b) Res = 1.0/K = 20 (c) Res = 1.5/K = 23

(d) Res = 2.0/K = 27 (e) Res = 2.5/K = 31 (f) Res = 3.0/K = 35

Fig S10. UMAP visualization of the p-values given by SCIPAC under different resolutions on the prostate cancer data. Res stands for resolution, and K stands for the number of cell clusters given by this resolution.

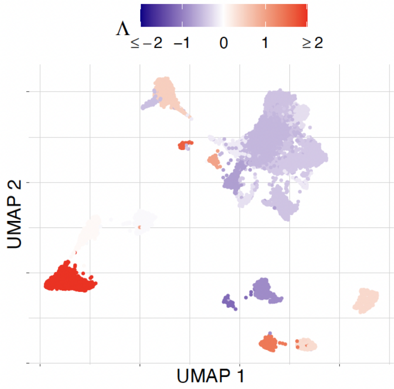

(a) Res = 0.5/K = 21

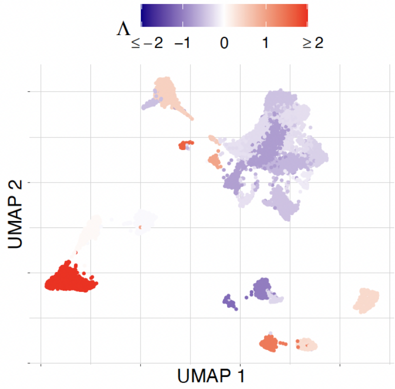

(b) Res = 1.0/K = 27

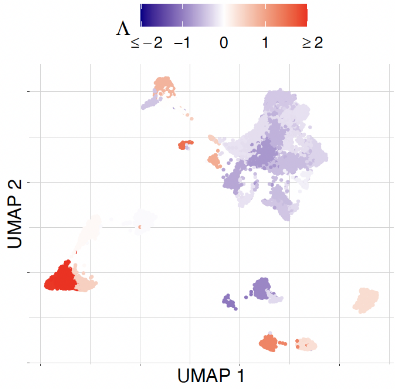

(c) Res = 1.5/K = 32

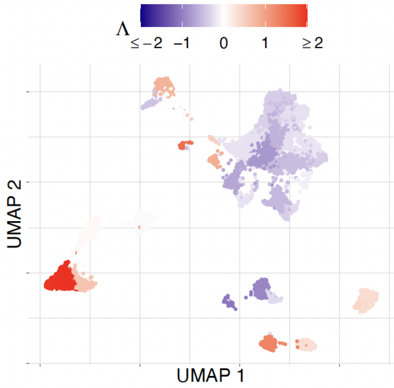

(d) Res = 2.0/K = 36

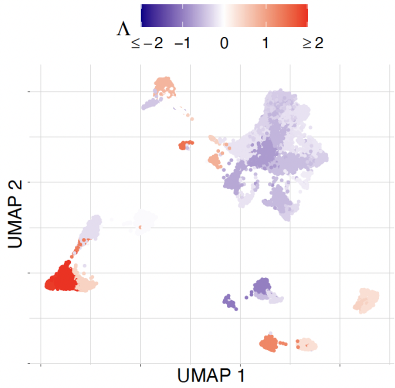

(e) Res = 2.5/K = 39

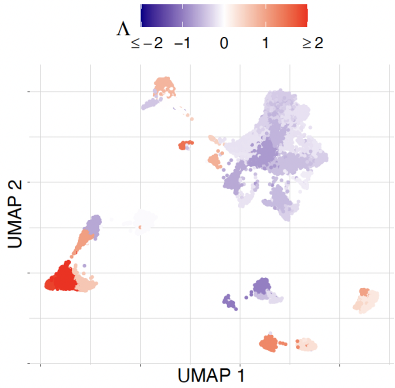

(f) Res = 3.0/K = 42

Fig S11. UMAP visualization of the estimated association strengths Λ given by SCIPAC under different resolutions on the breast cancer data. Res stands for resolution, and K stands for the number of cell clusters given by this resolution.

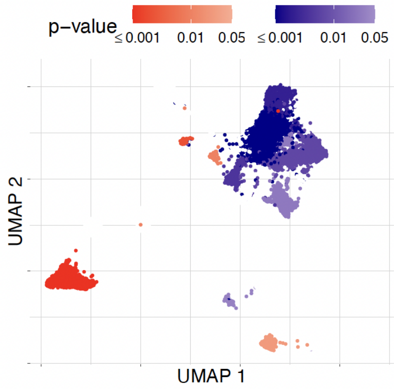

(a) Res = 0.5/K = 21

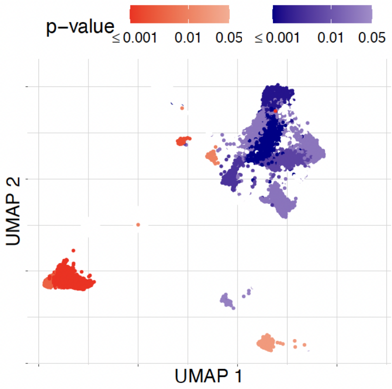

(b) Res = 1.0/K = 27

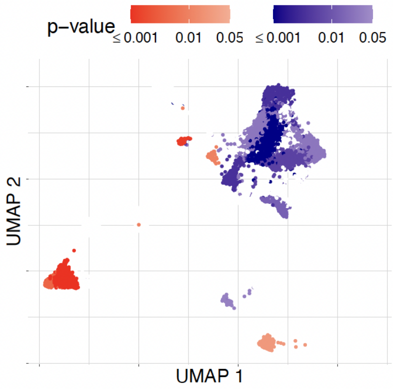

(c) Res = 1.5/K = 32

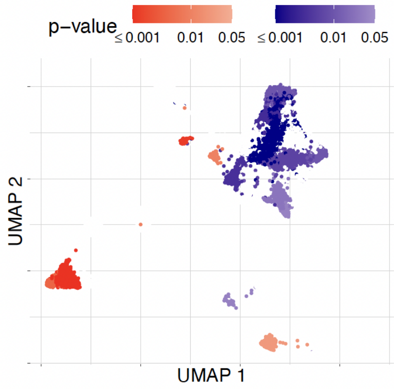

(d) Res = 2.0/K = 36

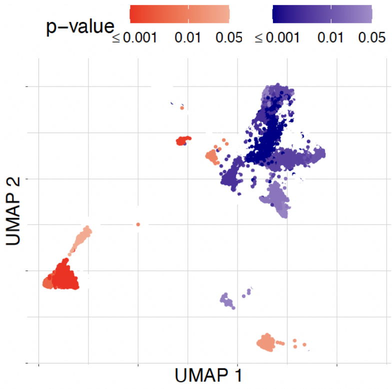

(e) Res = 2.5/K = 39

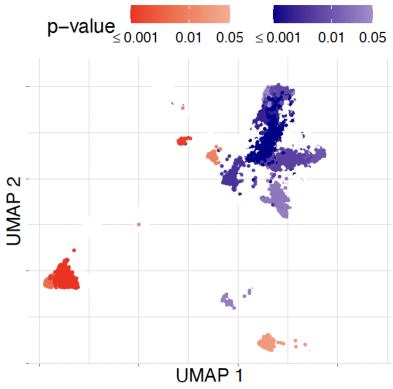

(f) Res = 3.0/K = 42

Fig S12. UMAP visualization of the p-values given by SCIPAC under different resolutions on the breast cancer data. Res stands for resolution, and K stands for the number of cell clusters given by this resolution.

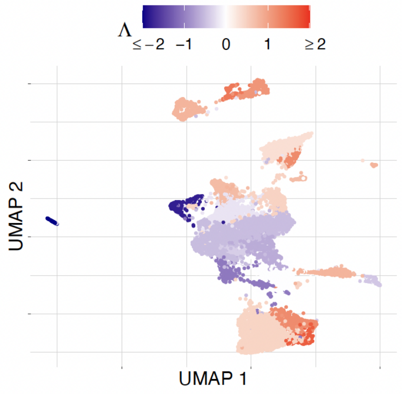

(a) Res = 0.5/K = 22

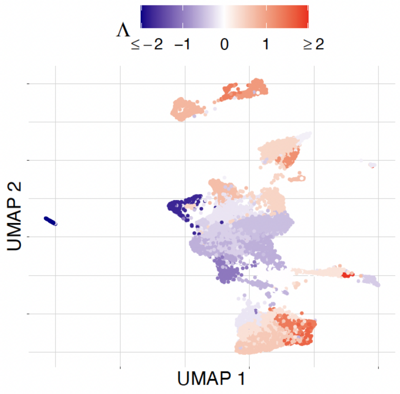

(b) Res = 1.0/K = 30

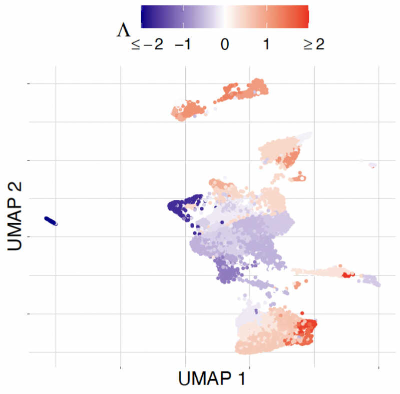

(c) Res = 1.5/K = 40

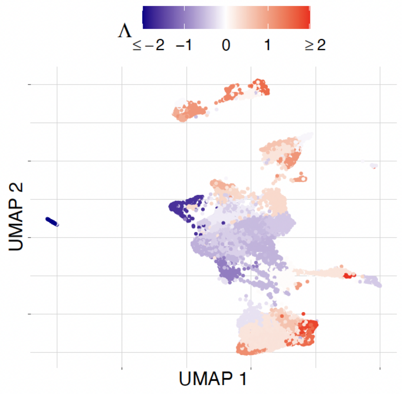

(d) Res = 2.0/K = 43

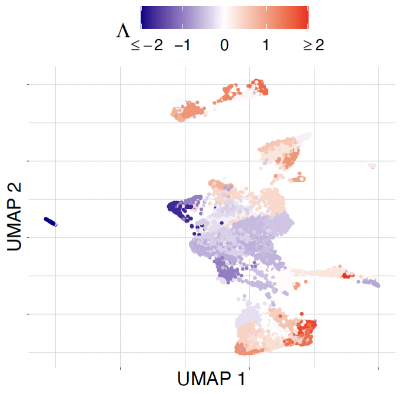

(e) Res = 2.5/K = 54

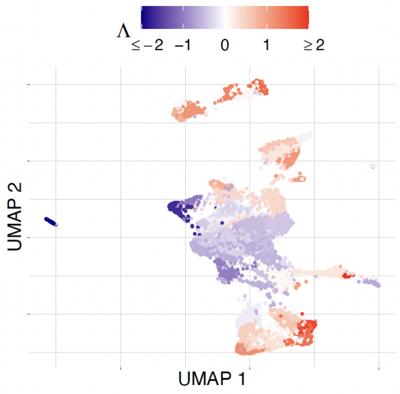

(f) Res = 3.0/K = 56

Fig S13. UMAP visualization of the estimated association strengths Λ given by SCIPAC under different resolutions on the lung cancer data. Res stands for resolution, and K stands for the number of cell clusters given by this resolution.

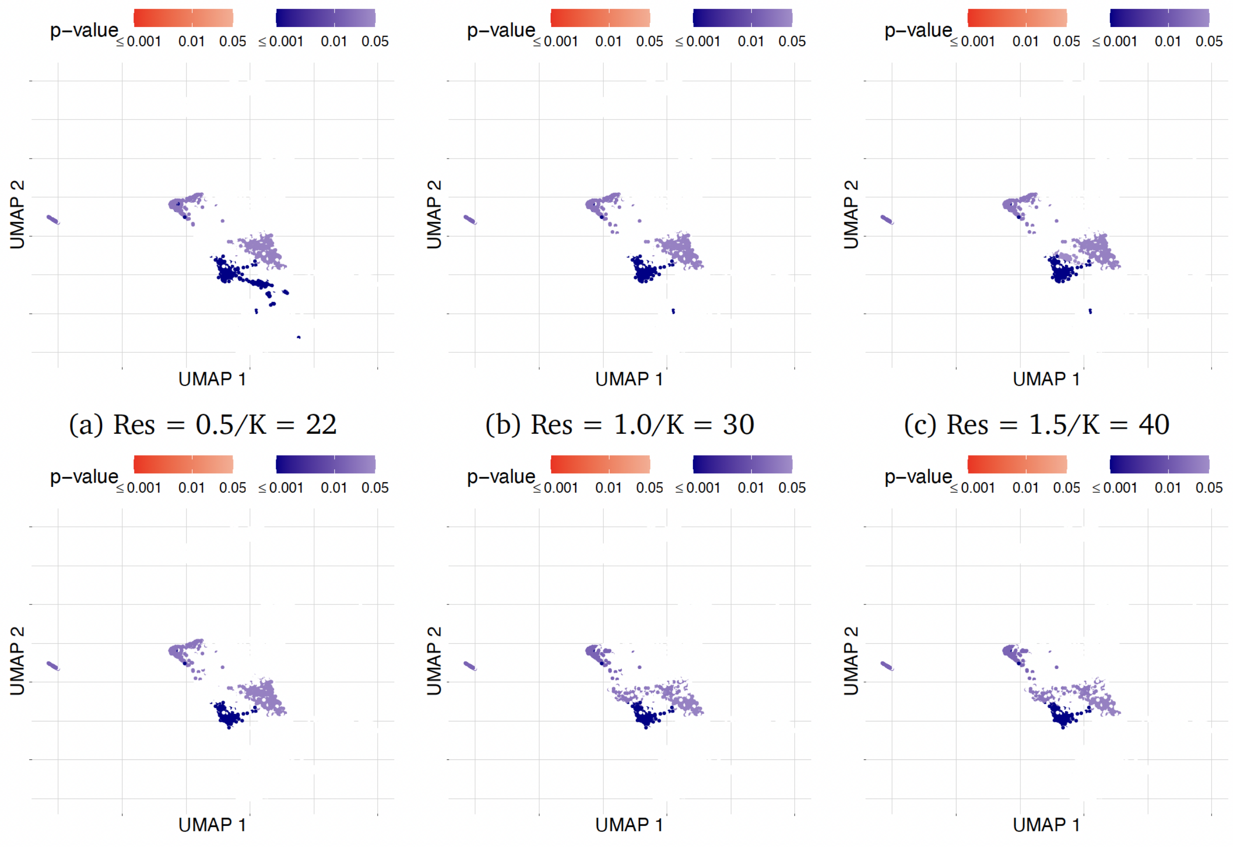

(a) Res = 0.5/K = 22

(b) Res = 1.0/K = 30

(c) Res = 1.5/K = 40

(d) Res = 2.0/K = 43

(e) Res = 2.5/K = 54

(f) Res = 3.0/K = 56

Fig S14. UMAP visualization of the p-values given by SCIPAC under different resolutions on the lung cancer data. Res stands for resolution, and K stands for the number of cell clusters given by this resolution.

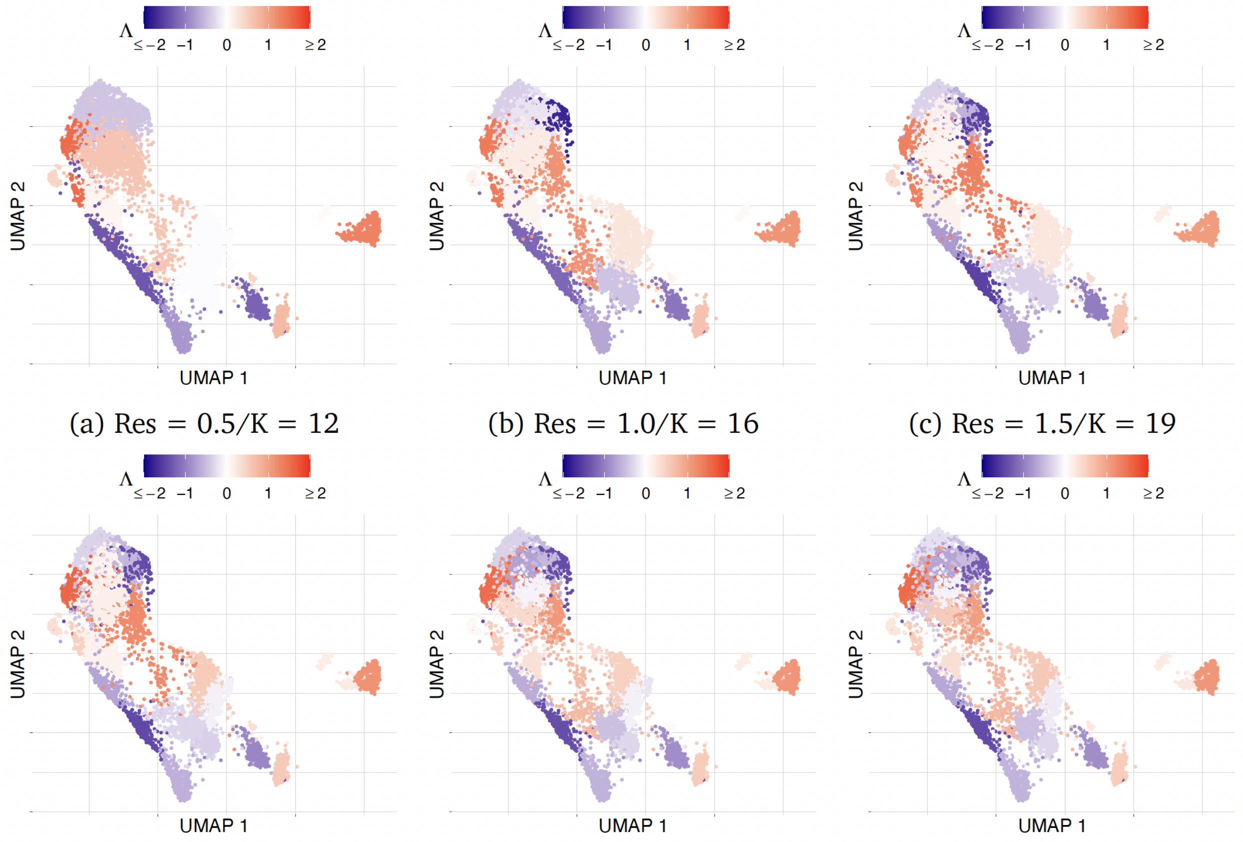

(a) Res = 0.5/K = 12

(b) Res = 1.0/K = 16

(c) Res = 1.5/K = 19

(d) Res = 2.0/K = 24

(e) Res = 2.5/K = 27

(f) Res = 3.0/K = 29

Fig S15. UMAP visualization of the estimated association strengths Λ given by SCIPAC under different resolutions on the FSHD data. Res stands for resolution, and K stands for the number of cell clusters given by this resolution.

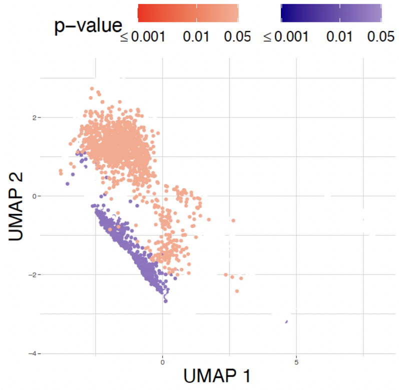

(a) Res = 0.5/K = 12

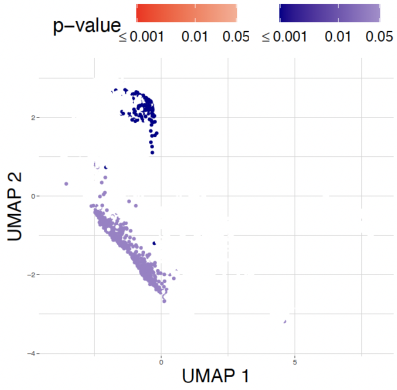

(b) Res = 1.0/K = 16

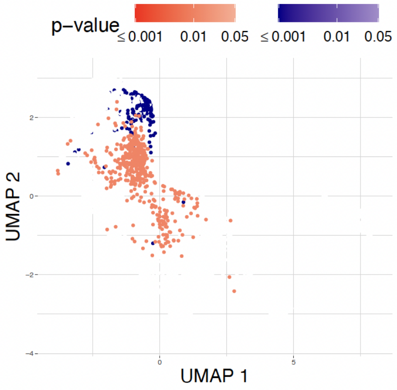

(c) Res = 1.5/K = 19

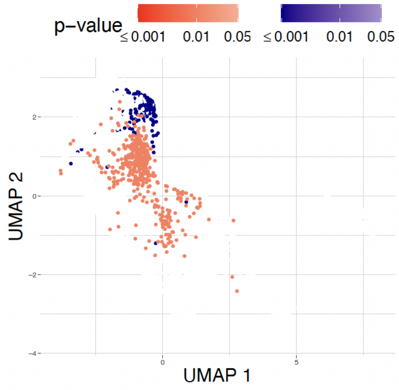

(d) Res = 2.0/K = 24

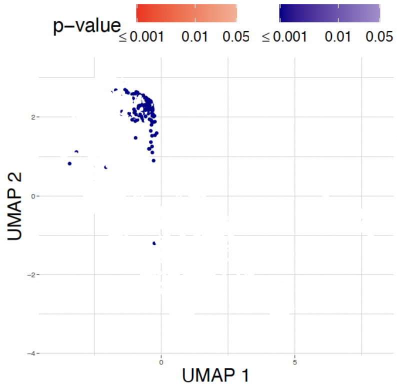

(e) Res = 2.5/K = 27

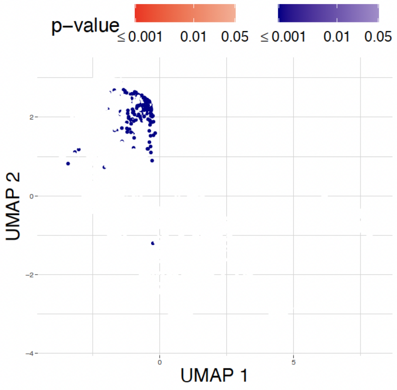

(f) Res = 3.0/K = 29

Fig S16. UMAP visualization of the p-values given by SCIPAC under different resolutions on the FSHD data. Res stands for resolution, and K stands for the number of cell clusters given by this resolution.

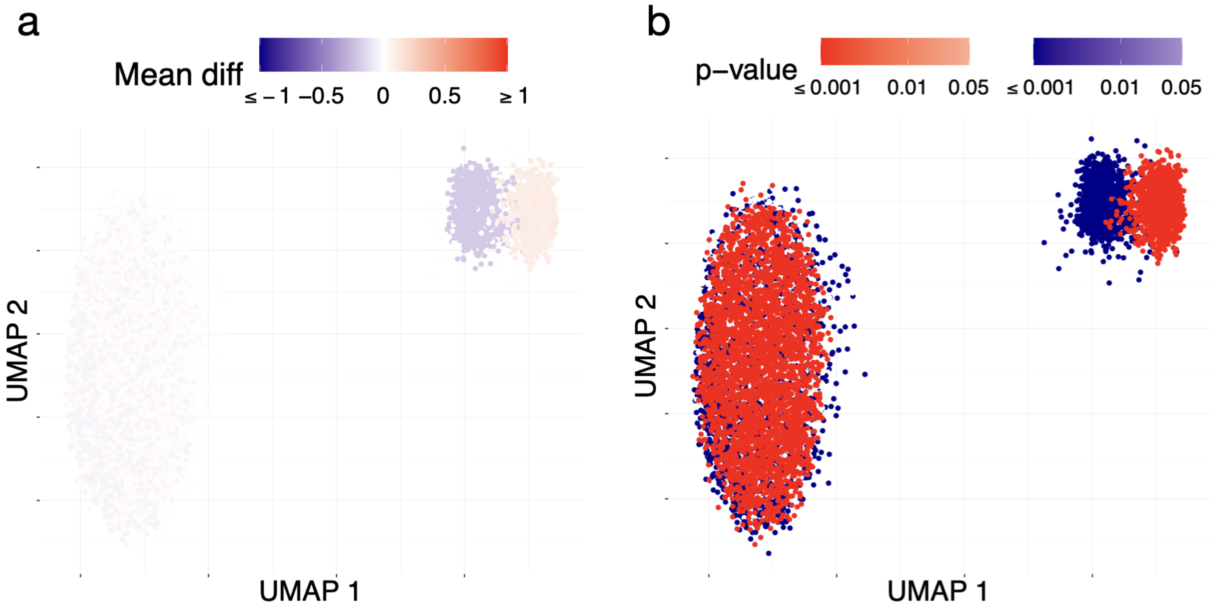

Fig S17. UMAP visualization of the simulated data under scheme I using the deconvolution-then-test strategy. (a) mean cell proportion differences between two sample groups. (b) p-values of the mean cell proportion differences using two sample t-test. It is noticeable that while this strategy can capture the truly positive/negative associated cells in the upper right corner of the plots, there is still an unacceptably large number of false positive discoveries.

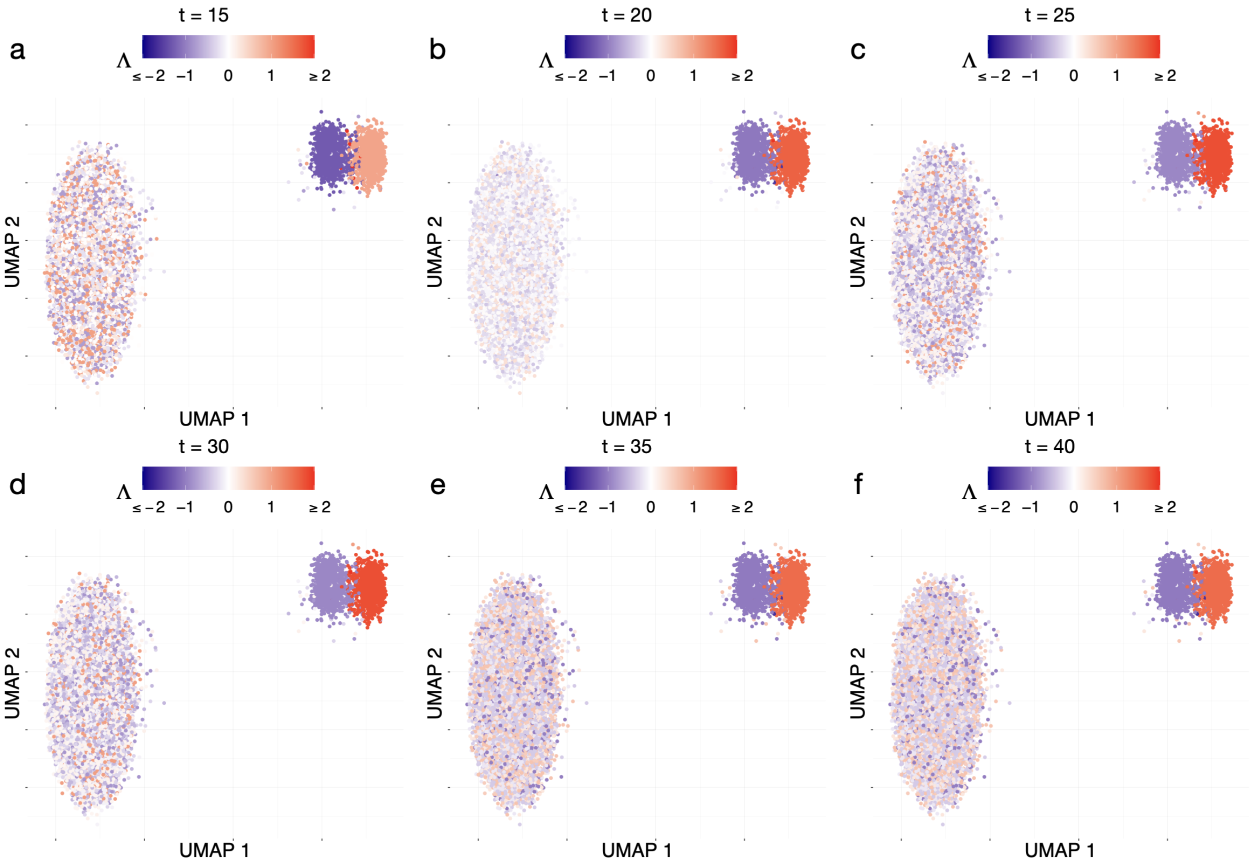

Fig S18. UMAP visualization of the simulated data under scheme I with the estimated association strength Λ, using different values of t, the proxy for the true association strength.

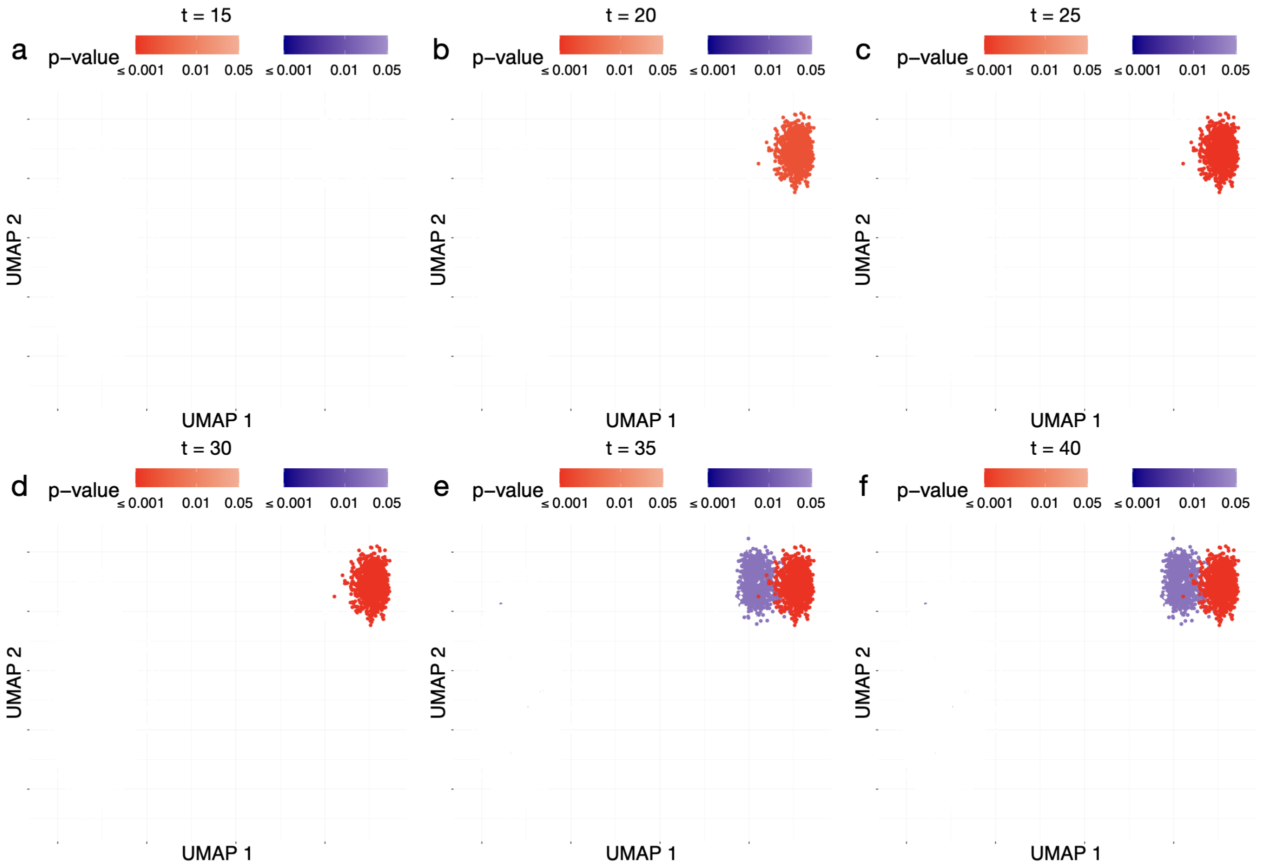

Fig S19. UMAP visualization of the simulated data under scheme I with the p values of the estimated association strength Λ, using different values of t, the proxy for the true association strength. As t increases, the estimated association strength becomes increasingly significant.

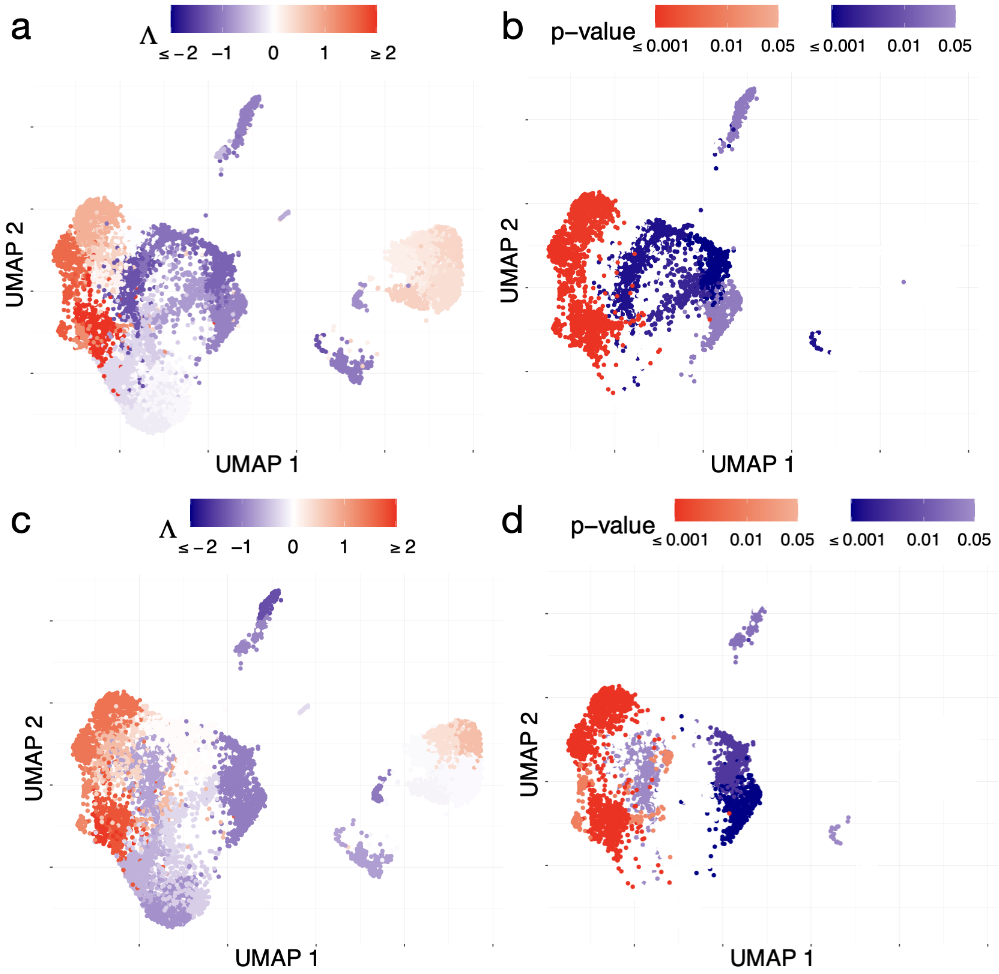

Fig S20. UMAP visualization of the scRNA-seq prostate cancer data, analyzed by SCIPAC using bulk samples collected from different years, namely parts 1 and 2. (a-b) Association strength and its corresponding p-value given by SCIPAC using bulk samples from part 1. (c-d) Association strength and its corresponding p-value given by SCIPAC using bulk samples from part 2.

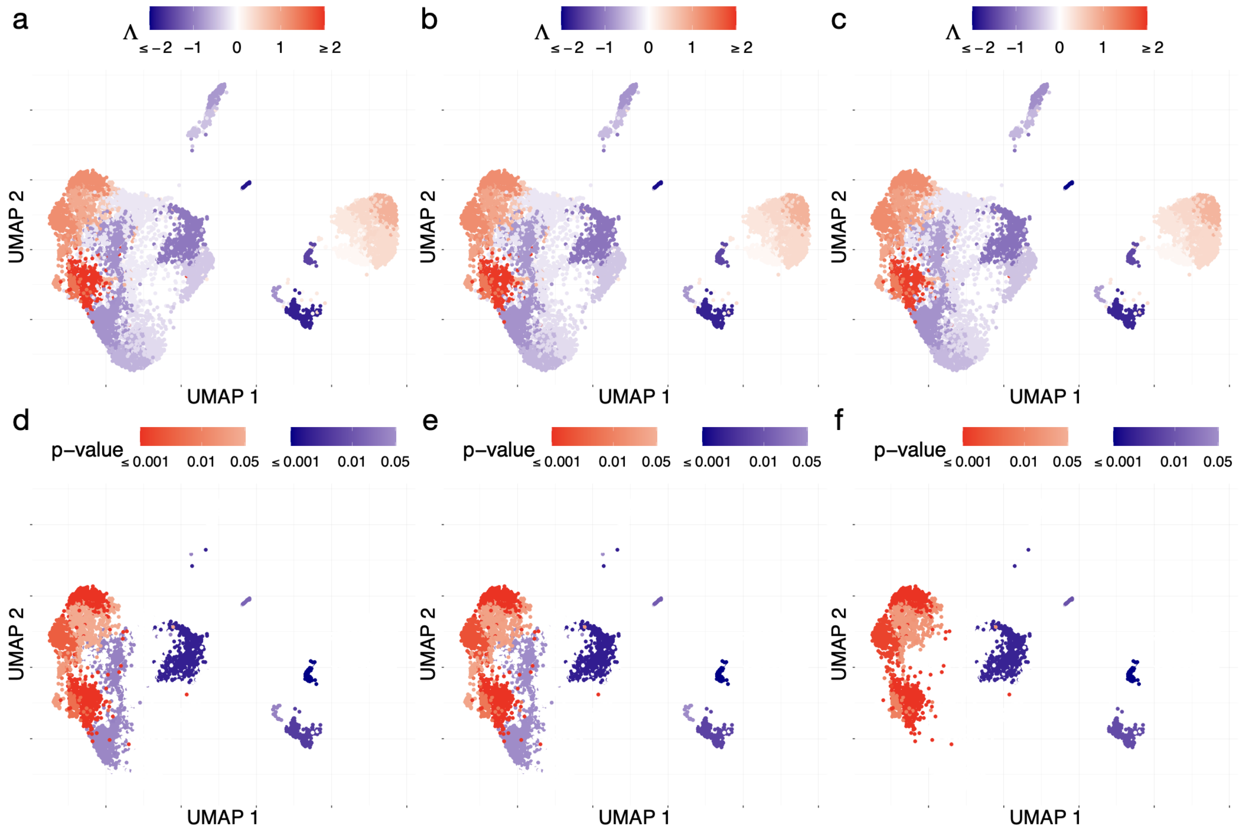

Fig S21. UMAP visualization of the prostate cancer data. (a-c) Association strength given by SCIPAC with the elastic net $\alpha = 0.3, 0.4, 0.5$, respectively. (d-f) p-values given by SCIPAC with the elastic net $\alpha = 0.3, 0.4, 0.5$, respectively. These results indicate SCIPAC is robust to the choice of α.

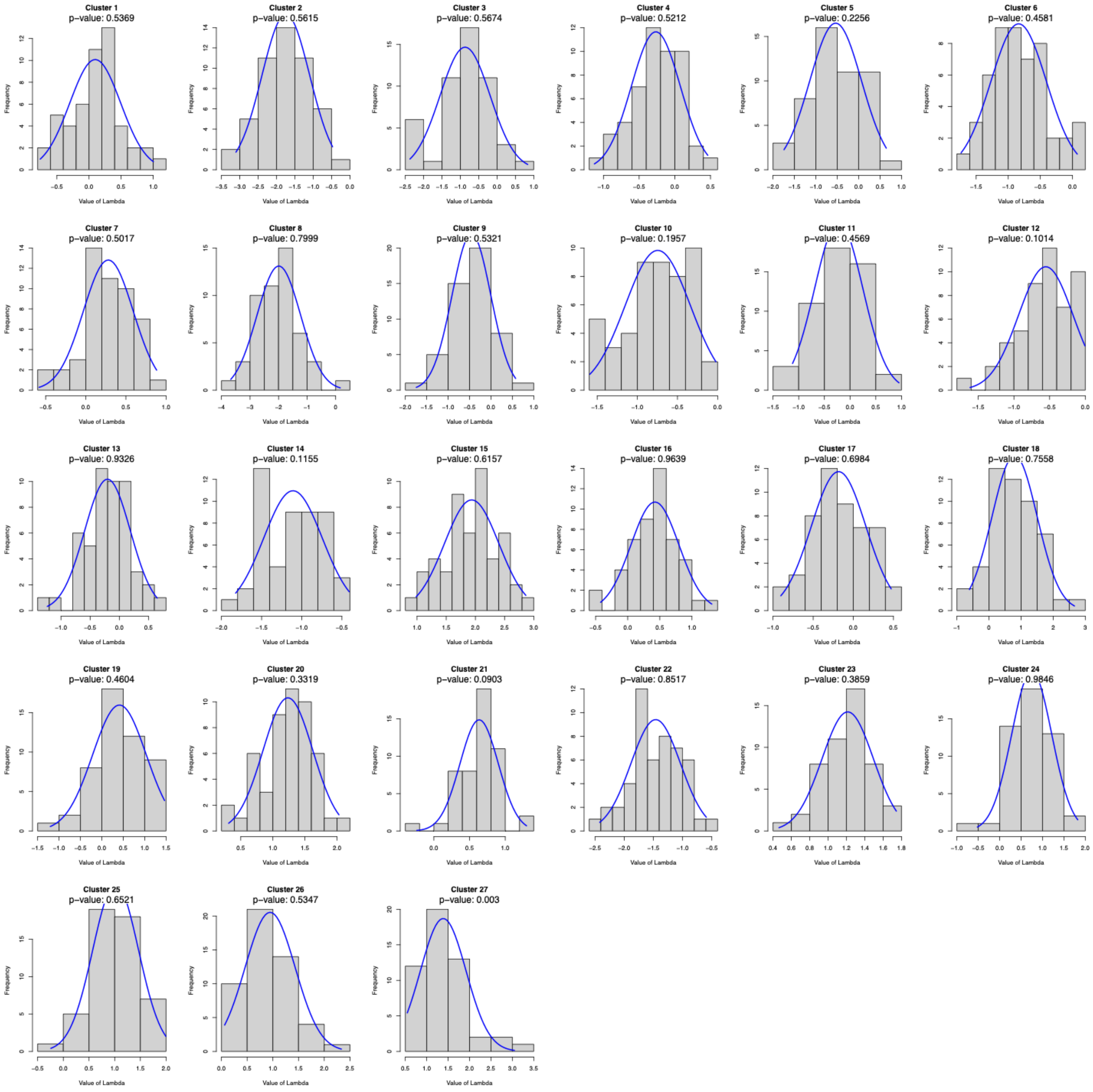

Fig S22. Histograms of the bootstrap-generated Λ_k's for each cluster in the prostate cancer data. The p-values are calculated using the Shapiro-Wilk's normality test. The blue lines are the estimated normal curves.

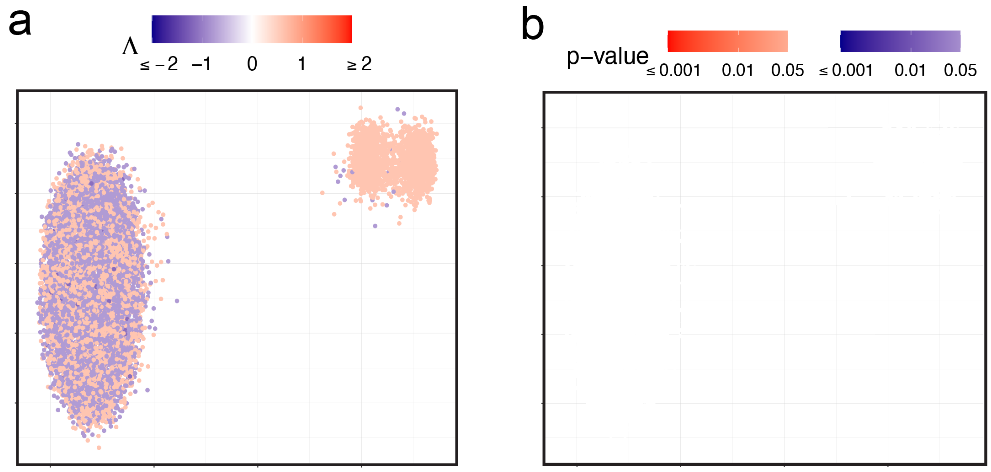

Fig S23. Results of SCIPAC on three metacells. Both plots are scatterplots of the two dimensional single-cell data given by UMAP. Points in the plots represents single cells, and they are colored differently in each subplot to reflect different information/results. **(a)** Association strengths Λ. Red/blue represents the sign of Λ, and the shade gives the absolute value of Λ. Every cell is colored red or blue since no Λ is exactly zero. **(b)** p-values. Only cells with p-value < 0.05 are colored red (positive association) or blue (negative association); others are colored white.

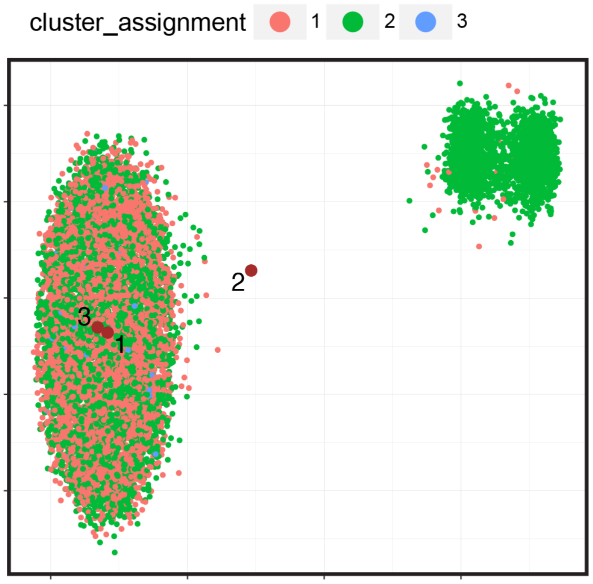

Fig S24. UMAP plot of single cells, color-coded based on their assignments into three meta-cells as determined by SEACells.

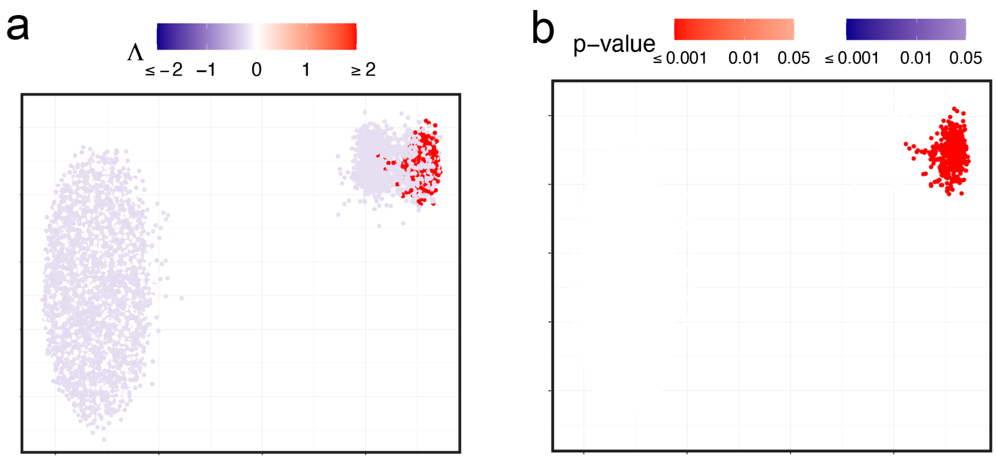

Fig S25. Results of SCIPAC on five metacells. Both plots are scatterplots of the two dimensional single-cell data given by UMAP. Points in the plots represents single cells, and they are colored differently in each subplot to reflect different information/results. **(a)** Association strengths Λ. Red/blue represents the sign of Λ, and the shade gives the absolute value of Λ. Every cell is colored red or blue since no Λ is exactly zero. **(b)** p-values. Only cells with p-value < 0.05 are colored red (positive association) or blue (negative association); others are colored white.

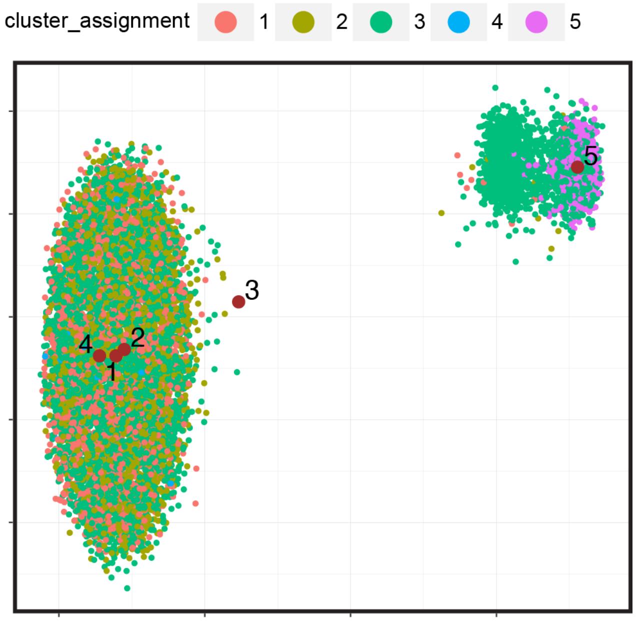

Fig S26. UMAP plot of single cells, color-coded based on their assignments into five meta-cells as determined by SEACells.

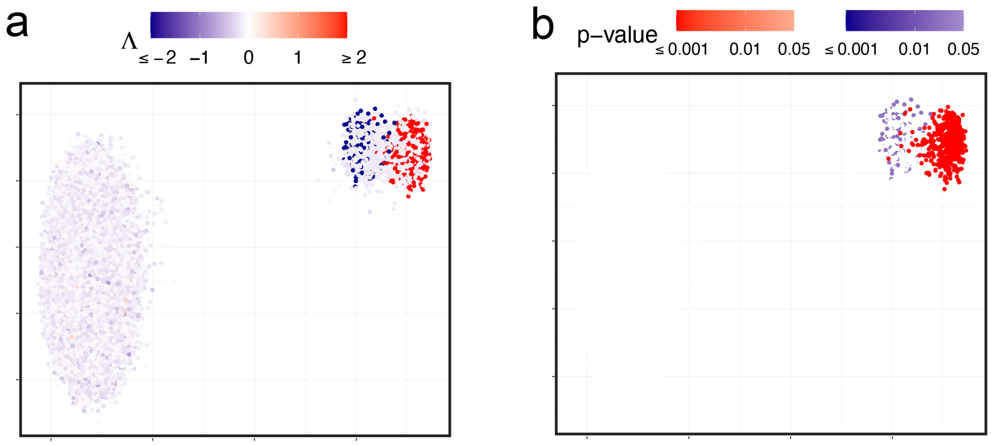

Fig S27. Results of SCIPAC on 20 metacells. Both plots are scatterplots of the two dimensional single-cell data given by UMAP. Points in the plots represents single cells, and they are colored differently in each subplot to reflect different information/results. **(a)** Association strengths Λ. Red/blue represents the sign of Λ, and the shade gives the absolute value of Λ. Every cell is colored red or blue since no Λ is exactly zero. **(b)** p-values. Only cells with p-value < 0.05 are colored red (positive association) or blue (negative association); others are colored white.

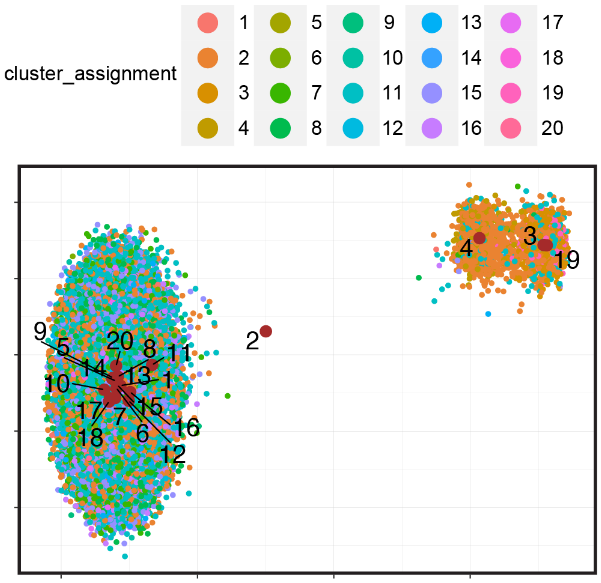

Fig S28. UMAP plot of single cells, color-coded based on their assignments into 20 metacells as determined by SEACells.

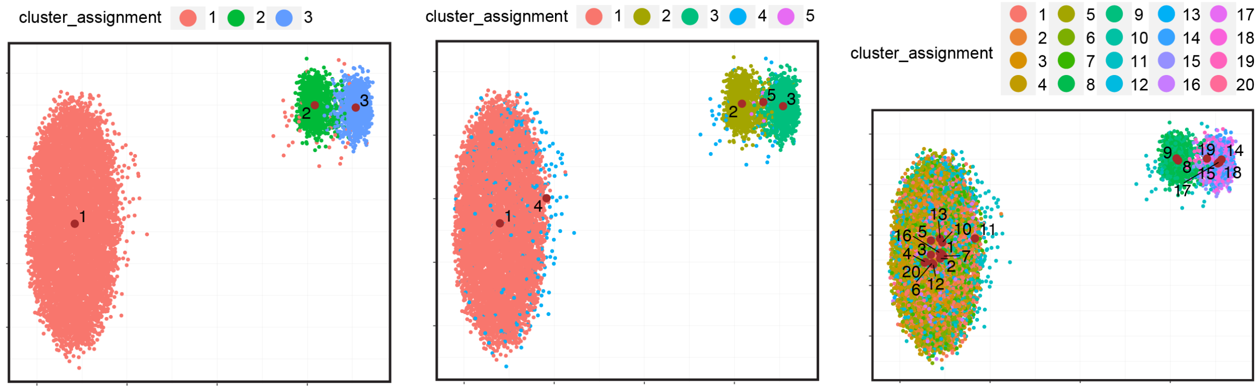

a Res=0.5/K=3 b Res=1.6/K=5 c Res=2.5/K=20

Fig S29. UMAP plot of single cells, color-coded based on their assignments into different clusters as determined by the Louvain algorithm used by SCIPAC. **(a)** 3 clusters. **(b)** 5 clusters. **(c)** 20 clusters.

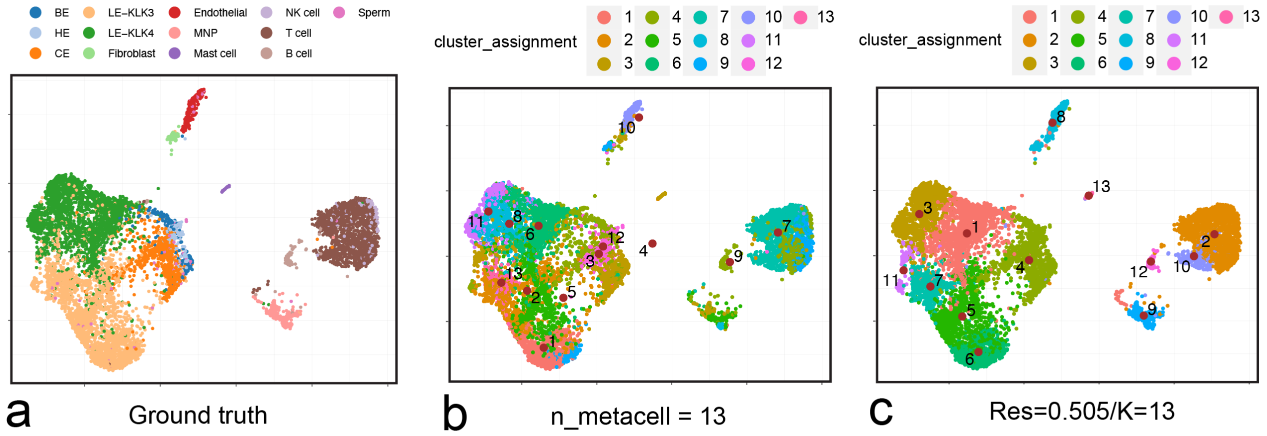

Fig S30. UMAP plot of single cells, color-coded based on their true cell types or their assignments into different clusters/metacells. **(a)** 13 true cell types. **(b)** 13 metacells given by SEACells. **(c)** 13 clusters as determined by the Louvain algorithm used by SCIPAC.

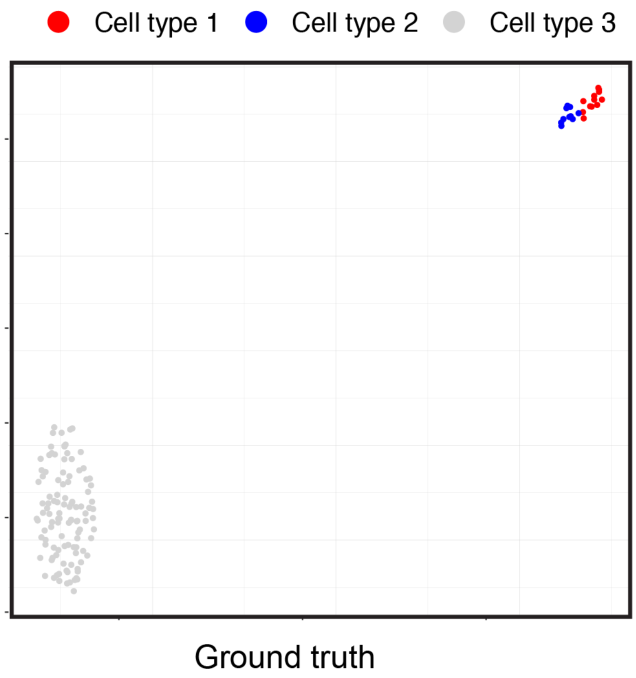

Fig S31. UMAP plot of metacells, color-coded according to the majority cell type of cells in the metacell.

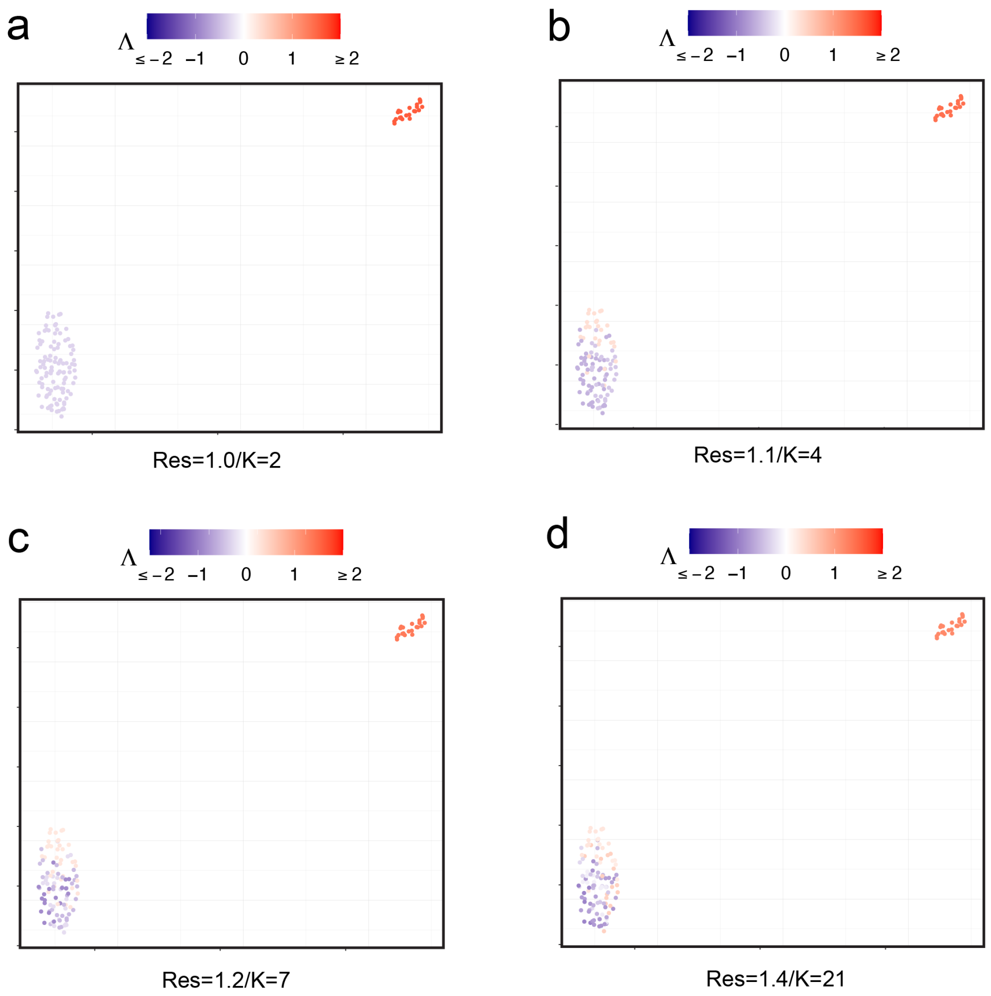

Fig S32. The estimated association strength given by SCIPAC under different resultions. Points in each plot represents metacells, and they are colored accoding to the association strengths Λ. **(a)** Resolution 1.0, which gives 2 clusters. **(b)** Resolution 1.1, which gives 4 clusters. **(c)** Resolution 1.2, which gives 7 clusters. **(d)** Resolution 1.4, which gives 21 clusters.

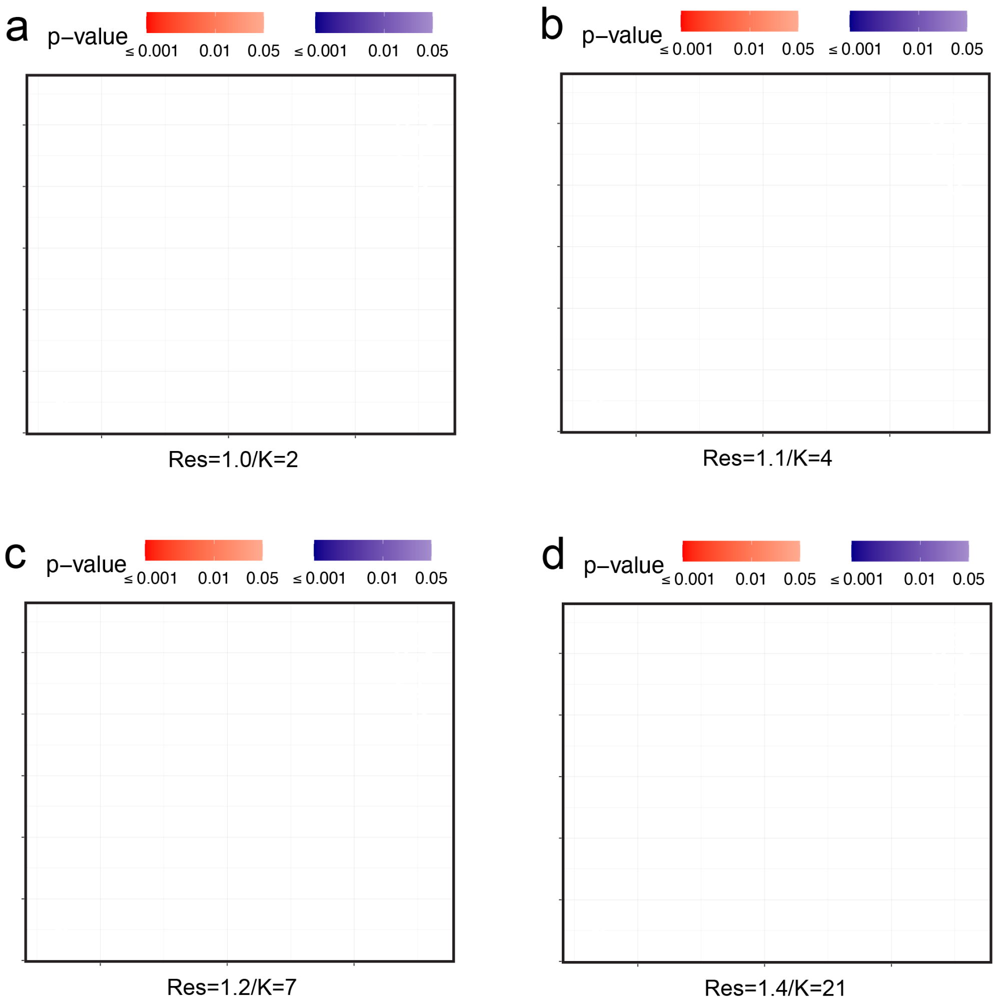

Fig S33. The p-values given by SCIPAC under different resultions. Points in each plot represents metacells, and they are colored accoding to the p-values. Only cells with p-value < 0.05 are colored red (positive association) or blue (negative association); others are colored white. **(a)** Resolution 1.0, which gives 2 clusters. **(b)** Resolution 1.1, which gives 4 clusters. **(c)** Resolution 1.2, which gives 7 clusters. **(d)** Resolution 1.4, which gives 21 clusters.

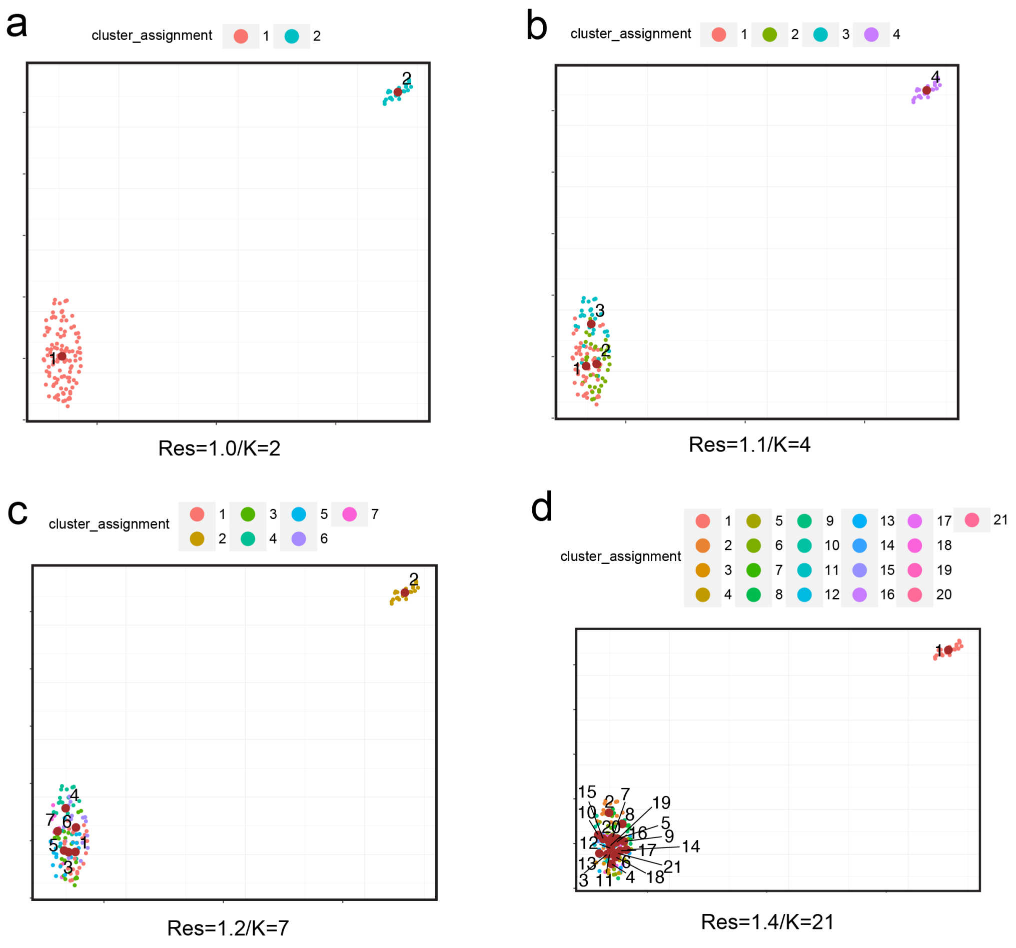

Fig S34. UMAP plot of metacells, color-coded based on their assignments into different numbers of clusters given by the Louvain algorithm. The centroids of the clusters are also highlighted. **(a)** Resolution 1.0, which gives 2 clusters. **(b)** Resolution 1.1, which gives 4 clusters. **(c)** Resolution 1.2, which gives 7 clusters. **(d)** Resolution 1.4, which gives 21 clusters.
