# Supplementary material for: SCIPAC: quantitative estimation of cell-phenotype associations
Source: Genome Biol. 2024 May 13;25:119. doi: 10.1186/s13059-024-03263-1 (PMC11089691; doi:10.1186/s13059-024-03263-1)
Supplement: Supplementary file 2 — Additional file 2. A vignette of the SCIPAC package. [file 13059_2024_3263_MOESM2_ESM.pdf]

SCIPAC Tutorial

- Introduction
- SCIPAC examples
    - Applying SCIPAC to data with a binary phenotype
        - Preparing scRNA-seq and bulk RNA-seq data
        - Clustering
        - Executing SCIPAC to identify phenotype-associated cells
        - Visualization of results
    - Applying SCIPAC to data with an ordinal phenotype
        - Clustering
        - Executing SCIPAC
        - Visualization of results
    - Applying SCIPAC to data with survival information
        - Working with TCGA database
        - Clustering
        - Executing SCIPAC
        - Visualization of results
- Session Info

Introduction

This SCIPAC R package contains the proposed SCIPAC algorithm (function `SCIPAC`), which is a computational method that identifies cells in single-cell data that are associated with a given phenotype.

In this tutorial (R version: 4.1.2), we use several examples to explain the functions in SCIPAC package. We first load the required package:

```
library(SCIPAC)
```

SCIPAC examples

SCIPAC uses input data consisting of three parts: single-cell RNA-seq data that measures the expression of p genes in m cells, bulk RNA-seq data that measures the expression of the same set of p genes in n samples/tissues, and the phenotype of each sample in the bulk RNA-seq data.

Applying SCIPAC to data with a binary phenotype

In this example, we use SCIPAC for simulated scRNA-seq and bulk RNA-seq data with a binary phenotype.

Preparing scRNA-seq and bulk RNA-seq data

We first load the scRNA-seq count data

```
sc.dat <- readRDS(system.file("extdata", "sim.raw.scRNA.rds", package = "SCIPAC"))
# Check the first few rows and columns.
print(sc.dat[1:4, 1:10])
```

```
##       Cell1 Cell2 Cell3 Cell4 Cell5 Cell6 Cell7 Cell8 Cell9 Cell10
## Gene1     0     0     0     0     0     0     0     0     0      0
## Gene2     2     0     0     0     1     4     0     3     4      1
## Gene3     0     3     0     5     6     0     1     5     0      5
## Gene4     0     0     0     0     0     0     1     1     0      2
```

In the simulated scRNA-seq data, rows stand for genes and columns stand for cells. The dimension of this data is

```
dim(sc.dat)
```

```
## [1] 10000 10000
```

The corresponding meta data for this scRNA-seq data is

```
sc.meta <- readRDS(system.file("extdata", "sim.raw.scRNA.meta.rds", package = "SCIPA
C"))
# Check the first few rows
head(sc.meta)
```

	Cell <chr>	Batch <chr>	Group <fct>	ExpLibSize <dbl>
1	Cell1	Batch1	Group1	71681.23
2	Cell2	Batch1	Group1	63091.46
3	Cell3	Batch1	Group3	74492.80
4	Cell4	Batch1	Group1	78405.93
5	Cell5	Batch1	Group1	51525.85
6	Cell6	Batch1	Group1	51776.67

6 rows

We next check the cell types

```
table(sc.meta$Group)
```

```
##
## Group1 Group2 Group3
##   7991   1013    996
```

There are three cell types, which are Group1, Group2, and Group3. The Group2 and Group3 cells are phenotype-associated cells while Group1 are not.

The bulk RNA-seq data is loaded in the following

```
bulk.dat <- readRDS(system.file("extdata", "bulk.dat.raw.rds", package = "SCIPAC"))
# Check the first few rows and columns
print(bulk.dat[1:4, 1:6])
```

```
##         pheno1.1   pheno1.2   pheno1.3   pheno1.4   pheno1.5   pheno1.6
## Gene1 0.00000000 0.00000000 0.00000000 0.00000000 0.00000000 0.00000000
## Gene2 0.37285248 0.37344484 0.37137879 0.37485306 0.37901400 0.37648699
## Gene3 0.25711978 0.26110376 0.26428917 0.26759723 0.26267130 0.26597588
## Gene4 0.06758342 0.06710196 0.06853341 0.06572419 0.06809027 0.06984578
```

The bulk RNA-seq data has been normalized. Rows stand for genes and columns stand for cells.

The corresponding meta data for this bulk data is

```
bulk.meta <- readRDS(system.file("extdata", "bulkRNA.meta.rds", package = "SCIPAC"))
# Check the first few rows
head(bulk.meta)
```

	**case.id**<br><chr>	**phenotype**<br><chr>
pheno1.1	pheno1.1	phenotype.1
pheno1.2	pheno1.2	phenotype.1
pheno1.3	pheno1.3	phenotype.1
pheno1.4	pheno1.4	phenotype.1
pheno1.5	pheno1.5	phenotype.1
pheno1.6	pheno1.6	phenotype.1

6 rows

We next check the phenotypes

```
table(bulk.meta$phenotype)
```

```
##
## phenotype.1 phenotype.2
##          50          50
```

There are 100 bulk samples with two classes, which are `phenotype.1` and `phenotype.2`. This can be simply considered as cancer vs. normal samples.

Since we have already had all the input data, let's perform the pre-processing steps.

```
sc.bulk.prec <- preprocess.sc.bulk.dat(sc.dat, bulk.dat, hvg = 1000)
```

The `preprocess.sc.bulk.dat` function is embedded in SCIPAC package, and it first finds the overlap genes of the scRNA-seq and bulk RNA-seq data and uses these genes for scRNA-seq and bulk data normalization. The normalization of the scRNA-seq data in SCIPAC uses the normalization steps in `Seurat` R package and the 1000 highly variable genes are identified. For the bulk RNA-seq data, since it has been normalized, we further take the log-transformation and use the 1000 highly variable genes identified in scRNA-seq data. Users can also use their own pre-processing steps for scRNA-seq and bulk RNA-seq data, as long as they have the identical set of features. The pre-processed scRNA-seq and bulk RNA-seq data are

```
sc.dat.prep <- sc.bulk.prec$sc.dat.preprocessed
bulk.dat.prep <- sc.bulk.prec$bulk.dat.preprocessed
```

Let's check the dimension for the pre-processed data

```
dim(sc.dat.prep)
```

```
## [1]  1000 10000
```

```
dim(bulk.dat.prep)
```

```
## [1] 1000  100
```

For both the pre-processed scRNA-seq and bulk RNA-seq data, rows stand for genes and columns stand for cells or bulk samples. The 1000 highly variable genes are used for downstream analysis.

We next perform dimension reduction on `sc.dat.prep` and `bulk.dat.prep`.

```
pca.res <- sc.bulk.pca(sc.dat.prep, bulk.dat.prep, do.pca.sc = FALSE, n.pc = 60)
```

The `sc.bulk.pca` function is embedded in SCIPAC package, and it uses PCA to perform the dimension reduction. By setting `do.pca.sc = FALSE`, `sc.bulk.pca` first performs PCA on the bulk data and then uses the rotation matrix on scRNA-seq data. If `do.pca.sc = TRUE`, the procedure is reversed. The number of chosen PCs, by default, is `n.pc = 60`. Users can also use their own dimension reduction methods. The outputs are

```
sc.dat.dim.redu <- pca.res$sc.dat.rot
bulk.dat.dim.redu <- pca.res$bulk.dat.rot
```

Let’s also check the dimension for the dimension-reduced data

```
dim(sc.dat.dim.redu)
```

```
## [1] 10000    60
```

```
dim(bulk.dat.dim.redu)
```

```
## [1] 100  60
```

For the dimension-reduced scRNA-seq and bulk RNA-seq data, rows stand for cells or bulk samples and columns stand for the first 60 PCs.

Clustering

After pre-processing, we cluster the scRNA-seq data into K groups and obtain K cluster centroids before using SCIPAC to identify the phenotype-associated cells.

```
ct.res <- seurat.ct(sc.dat.dim.redu, res = 1.5)
```

```
## Computing nearest neighbor graph
```

```
## Computing SNN
```

```
## Modularity Optimizer version 1.3.0 by Ludo Waltman and Nees Jan van Eck
##
## Number of nodes: 10000
## Number of edges: 204925
##
## Running Louvain algorithm...
## Maximum modularity in 10 random starts: 0.4821
## Number of communities: 9
## Elapsed time: 0 seconds
```

```
## 5 singletons identified. 4 final clusters.
```

The `seurat.ct` function is embedded in SCIPAC package. It uses the clustering steps in `Seurat` R package. Users can also use their own clustering methods to annotate each cell. The parameter `res` controls resolutions. A larger values of `res` leads to more clusters. We set `res = 1.5` in our example.

```
summary(ct.res)
```

```
##                Length Class      Mode   
## k                1    -none-     numeric
## ct.assignment    1    data.frame list   
## centers        240    -none-     numeric
```

There are three elements in the list `ct.res`, which are `k`, `ct.assignment`, and `centers`. Let's look at them one by one.

```
k <- ct.res$k
print(k)
```

```
## [1] 4
```

The element `k` is a numeric value, indicating the total number of clusters.

```
ct.assignment <- ct.res$ct.assignment
head(ct.assignment)
```

	cluster_assignment <dbl>
Cell1	1
Cell2	1

Cell3	3
Cell4	1
Cell5	1
Cell6	1

6 rows

The element `ct.assignment` is a data frame with one column named `cluster_assignment`. The cluster assignments should be numeric values. Row names of `ct.assignment` are cell IDs.

```
centers <- ct.res$centers
print(centers[1:6, 1:3])
```

```
##              [,1]         [,2]         [,3]
## PC1 -0.0685890640 -0.309812316  0.846524159
## PC2  0.5173826752 -1.941603773 -2.002010893
## PC3 -0.0138228728  0.024154444  0.091536070
## PC4 -0.0088310989  0.066406564  0.011236493
## PC5  0.0088485606 -0.043443053 -0.022339515
## PC6  0.0005926077  0.002267678 -0.004783318
```

The element `centers` is a data matrix, whose rows are PCs and columns are the centroids of each cluster.

For the clustering step, users do not have to use the function `seurat.ct` to obtain clusters. As long as users can generate a list with three elements named `k`, `ct.assignment`, and `centers`, as described above, it should work in the downstream analysis. Please note that the column name of `ct.assignment` should be named `cluster_assignment`.

Executing SCIPAC to identify phenotype-associated cells

We first create the binary labels for bulk samples

```
library(dplyr)
```

```
##
## Attaching package: 'dplyr'
```

```
## The following objects are masked from 'package:stats':
##
##     filter, lag
```

```
## The following objects are masked from 'package:base':
##
##     intersect, setdiff, setequal, union
```

```
y <- c(rep(0, 50), rep(1, 50)) %>% as.factor()
```

The first 50 bulk samples are treated as class 0 and the remaining 50 samples are treated as class 1. In real application, we can say the first 50 samples are normal samples and the remaining 50 samples are cancer samples.

After obtaining all the necessary input, we can finally use `SCIPAC` to identify the phenotype-associated cells.

```
SCIPAC.res <- SCIPAC(bulk.dat = bulk.dat.dim.redu, y = y,
                     family = "binomial", ct.res = ct.res,
                     ela.net.alpha = 0.4, bt.size = 50,
                     numCores = 7, CI.alpha = 0.05, nfold = 10)
```

In the `SCIPAC` function, we set `family = "binomial"` since we use binary input. For other parameters `ela.net.alpha`, `bt.size`, `numCores`, and `CI.alpha`, we use the default values in `SCIPAC`. We next have a look at the output results.

```
head(SCIPAC.res)
```

	cluster_assignment <dbl>	Lambda.est <dbl>	Lambda.upper <dbl>	Lambda.lower <dbl>	sig <fct>
Cell1	1	-0.1436426	0.1578325	-0.4451178	Not.sig
Cell2	1	-0.1436426	0.1578325	-0.4451178	Not.sig
Cell3	3	2.6111716	3.6921852	1.5301580	Sig.pos
Cell4	1	-0.1436426	0.1578325	-0.4451178	Not.sig
Cell5	1	-0.1436426	0.1578325	-0.4451178	Not.sig
Cell6	1	-0.1436426	0.1578325	-0.4451178	Not.sig

6 rows | 1-6 of 7 columns

The rownames of `SCIPAC.res` are cell IDs. There are six columns in `SCIPAC.res`. The `cluster_assignment` is the cluster indices for each cell. `Lambda.est` is the estimated strength of the association between cells and a given phenotype. `Lambda.upper` and `Lambda.lower` are the upper and lower confidence intervals for `Lambda.est`. `sig` contains values Not.sig, Sig.pos and Sig.neg. It roughly

describes whether a cell is significantly positive- or negative-associated with a phenotype. `log.pval` is the $\log_{10}$ (p-values) for `Lambda.est`. **Note that the absolute values of `log.pval` is the absolute values of** $\log_{10}$ (p-values). The signs of `log.pval` correspond to postive or negative values of `Lambda.est`.

Visualization of results

We use UMAP to visually present SCIPAC's output. We first load necessary R packages and function for creating plots and do some adjustments for our data.

```
library(uwot)
```

```
## Loading required package: Matrix
```

```
library(ggplot2)
library(ggrepel)
library(ggthemes)
library(cowplot)
```

```
##
## Attaching package: 'cowplot'
```

```
## The following object is masked from 'package:ggthemes':
##
##     theme_map
```

```
library(ggnewscale)

do_umap <- function(V) {
  umap(
    X = V,
    n_threads = 6,
    n_neighbors = 30L,
    n_components = 2L,
    metric = 'cosine',
    n_epochs = NULL,
    learning_rate = 1.0,
    min_dist = 0.3,
    spread = 1.0,
    set_op_mix_ratio = 1.0,
    local_connectivity = 1L,
    repulsion_strength = 1,
    negative_sample_rate = 1,
    a = NULL,
    b = NULL,
    fast_sgd = FALSE,
    verbose = FALSE
  )
}

rownames(sc.meta) <- sc.meta$Cell
all(rownames(sc.meta) == rownames(SCIPAC.res))
```

```
## [1] TRUE
```

```
# Use UMAP to obtain two-dimension representations.
sc.umap <- do_umap(sc.dat.dim.redu)
colnames(sc.umap) <- c("umap.X1", "umap.X2")
```

```
# Create the data frame for ggplot
plot.dat <- cbind(sc.meta, SCIPAC.res)
all(rownames(plot.dat) == rownames(sc.umap))
```

```
## [1] TRUE
```

```
plot.dat <- cbind(plot.dat, sc.umap)

# Assign 0 values of log.pval to insignificant cells
plot.dat$log.pval.adj <- ifelse((plot.dat$sig == "Not.sig"),
                                0, plot.dat$log.pval)
```

```
# Adjust the cell type lables and create the cell type plot
plot.dat$phe <- NA
plot.dat[which(plot.dat$Group == "Group1"), ]$phe <- "Cell type 3"
plot.dat[which(plot.dat$Group == "Group2"), ]$phe <- "Cell type 2"
plot.dat[which(plot.dat$Group == "Group3"), ]$phe <- "Cell type 1"

plot.dat$phe <- factor(plot.dat$phe, levels = c("Cell type 1",
                                                "Cell type 2",
                                                "Cell type 3"))
colors.cell <- c("red", "blue", "lightgrey")
p1 <- ggplot(plot.dat) +
  geom_point(aes(x=umap.X1, y=umap.X2, col = phe, fill = phe)) +
  scale_color_manual(name = "Phenotype", values = colors.cell) +
  scale_fill_manual(name = "Phenotype", values = colors.cell) +
  guides(colour = guide_legend(override.aes = list(size=4))) +
  theme(legend.title = element_blank(),
        legend.position= "top",
        axis.text.x = element_blank(),
        axis.text.y = element_blank(),
        panel.background = element_blank(),
        panel.grid = element_line(color = "lightgrey", size = 0.1),
        legend.key = element_blank(),
        legend.background = element_rect(fill = "white", size = 0.1))+
  labs(x = "UMAP 1", y = "UMAP 2")

# Control the range of the estimate of Lambda.Psi within -2 and 2
plot.dat$Lambda.adj <- plot.dat$Lambda.est
if(sum(plot.dat$Lambda.est <= -2) > 0){
  plot.dat[which(plot.dat$Lambda.est <= -2), ]$Lambda.adj <- -2
}
if(sum(plot.dat$Lambda.est >= 2) > 0){
  plot.dat[which(plot.dat$Lambda.est >= 2), ]$Lambda.adj <- 2
}
p2 <- ggplot(plot.dat) +
  geom_point(aes(x=umap.X1, y=umap.X2, col=Lambda.adj)) +
  scale_color_gradient2(expression(Lambda), low="darkblue",high='red', mid = "white",
midpoint = 0,
                        limits = c(-2, 2), breaks = c(-2, -1, 0, 1, 2),
                        labels = c(expression(phantom(x) <= -2), -1, 0, 1, expression
(phantom(x) >= 2))) +
```

```
  theme(legend.title = element_text(family = "sans", size = 15),
        legend.position= "top",
        axis.text.x = element_blank(),
        axis.text.y = element_blank(),
        panel.background = element_blank(),
        panel.grid = element_line(color = "lightgrey", size = 0.1))+
  labs(x = "UMAP 1", y = "UMAP 2")

# Control the range of log.pval.adj within -3 and 3
plot.dat$pval.adj.adj <- plot.dat$log.pval.adj
if(sum(plot.dat$log.pval.adj <= -3) > 0){
  plot.dat[which(plot.dat$log.pval.adj <= -3), ]$pval.adj.adj <- -3
}
if(sum(plot.dat$log.pval.adj >= 3) > 0){
  plot.dat[which(plot.dat$log.pval.adj >= 3), ]$pval.adj.adj <- 3
}

plot.dat.positive <- plot.dat[which(plot.dat$pval.adj.adj > 0), ]
plot.dat.positive$pval.adj.adj <- -plot.dat.positive$pval.adj.adj

plot.dat.negative <- plot.dat[which(plot.dat$log.pval.adj <= 0), ]

p3 <- ggplot() +
  geom_point(data = plot.dat.negative, aes(x=umap.X1, y=umap.X2, col=pval.adj.adj)) +
  scale_color_gradient2('    ', low="darkblue",high='white', mid = "white", midpoint = 
0,
                        limits = c(-3, -1.3), breaks = c(-3, -2, -1.3),
                        labels = c(expression(phantom(x) <= 0.001), 0.01, 0.05),
                        na.value = "white") +
  new_scale_color() +
  geom_point(data = plot.dat.positive, aes(x=umap.X1, y=umap.X2, col=pval.adj.adj)) +
  scale_color_gradient2("p-values", low="red",high='white', mid = "white", midpoint = 
0,
                        limits = c(-3, -1.3), breaks = c(-3, -2, -1.3),
                        labels = c(expression(phantom(x) <= 0.001), 0.01, 0.05),
                        na.value = "white") +
  theme(legend.title = element_text(family = "sans", size = 15),
        legend.position= "top",
        axis.text.x = element_blank(),
        axis.text.y = element_blank(),
        panel.background = element_blank(),
        panel.grid = element_line(color = "lightgrey", size = 0.1))+
  labs(x = "UMAP 1", y = "UMAP 2")
```

Let’s view the final plot results. It can be noted that SCIPAC correctly captures the two phenotype-associated cell types most of time in this simulated data with default settings.

```
cowplot::plot_grid(p1, p2, p3, nrow = 1)
```

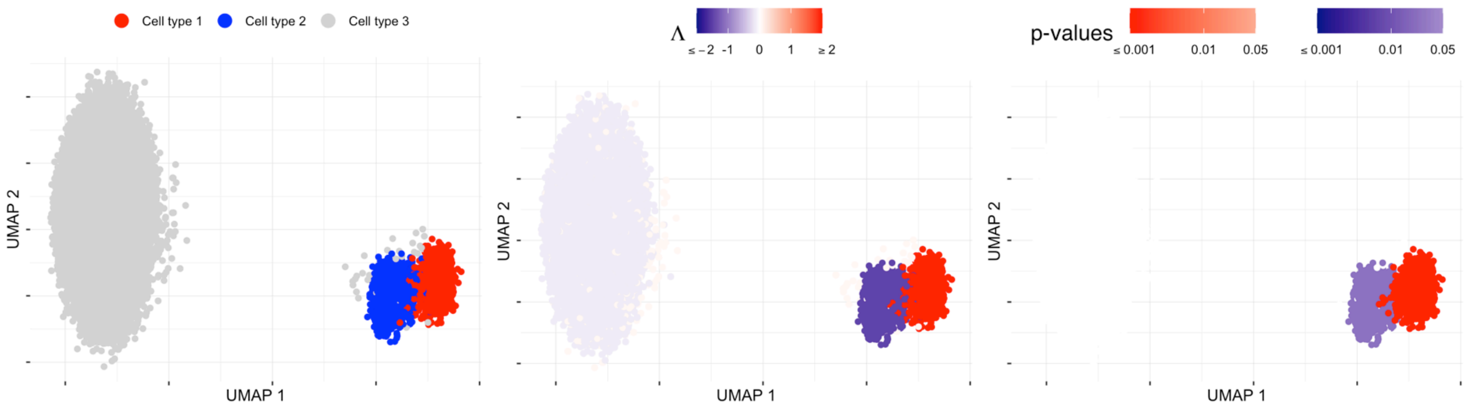

Applying SCIPAC to data with an ordinal phenotype

Next, we use another simulated scRNA-seq data and bulk RNA-seq data with ordinal phenotype information in SCIPAC.

Clustering

The pre-processing steps are the same as those mentioned in the previous section. Therefore, we omit the pre-processing steps and directly load the data.

```
# Load the pre-processed and dimension-reduced scRNA-seq data
sc.dat.ordinal <- readRDS(system.file("extdata", "sc.dat.ordinal.rds", package = "SCI
PAC"))
# Load the meta data for scRNA-seq data
sc.meta.ordinal <- readRDS(system.file("extdata", "sim.raw.scRNA.meta.ordinal.rds", p
ackage = "SCIPAC"))
# Load the pre-processed and dimension-reduced bulk RNA-seq data
bulk.dat.ordinal <- readRDS(system.file("extdata", "bulk.dat.ordinal.rds", package =
"SCIPAC"))
# Load the meta data for bulk RNA-seq data
bulk.meta.ordinal <- readRDS(system.file("extdata", "bulkRNA.meta.ordinal.rds", packa
ge = "SCIPAC"))
```

Let's check the first few lines of `sc.meta.ordinal` and `bulk.meta.ordinal`

```
head(sc.meta.ordinal)
```

Cell	Batch	Group	ExpLibSize	Step

	<chr>	<chr>	<fct>	<dbl>	<dbl>
1	Cell1	Batch1	Path2	71681.23	1544
2	Cell2	Batch1	Path2	63091.46	326
3	Cell3	Batch1	Path1	74492.80	7
4	Cell4	Batch1	Path3	78405.93	627
5	Cell5	Batch1	Path2	51525.85	63
6	Cell6	Batch1	Path4	51776.67	174

6 rows

```
table(sc.meta.ordinal$Group)
```

```
##
## Path1 Path2 Path3 Path4
##  2491  2516  2479  2514
```

There are four paths in this generated scRNA-seq data and each path has more or less the same number of cells.

```
head(bulk.meta.ordinal)
```

	**case.id**<br><chr>	**Stages**<br><chr>
stage1.1	stage1.1	Stage I
stage1.2	stage1.2	Stage I
stage1.3	stage1.3	Stage I
stage1.4	stage1.4	Stage I
stage1.5	stage1.5	Stage I
stage1.6	stage1.6	Stage I

6 rows

```
table(bulk.meta.ordinal$Stages)
```

```
##
##   Stage I  Stage II Stage III  Stage IV
##        50        50        50        50
```

The bulk samples are divided into four stages, representing different developmental stages.

Similar to the steps for the binary information, we first perform the clustering.

```
ct.res.ordinal <- seurat.ct(sc.dat.ordinal, res = 2.0)
```

```
## Computing nearest neighbor graph
```

```
## Computing SNN
```

```
## Modularity Optimizer version 1.3.0 by Ludo Waltman and Nees Jan van Eck
##
## Number of nodes: 10000
## Number of edges: 608841
##
## Running Louvain algorithm...
## Maximum modularity in 10 random starts: 0.7489
## Number of communities: 16
## Elapsed time: 0 seconds
```

Executing SCIPAC

Before applying SCIPAC, we first obtain the input phenotype labels `y`.

```
all(rownames(bulk.dat.ordinal) == rownames(bulk.meta.ordinal)) # TRUE
```

```
## [1] TRUE
```

```
y <- factor(bulk.meta.ordinal$Stages, levels = c("Stage I", "Stage II", "Stage III",
"Stage IV"))
```

Let's run SCIPAC for ordinal data.

```
SCIPAC.res.ordinal <- SCIPAC(bulk.dat = bulk.dat.ordinal, y = y,
                             family = "cumulative", ct.res = ct.res.ordinal,
                             ela.net.alpha = 0.4, bt.size = 50,
                             numCores = 7, CI.alpha = 0.05, nfold = 10)
```

We set `family = "cumulative"` for ordinal input `y` and use the default values for other parameters.

Visualization of results

Let's view the results generated by SCIPAC. For the cell paths, we use two-dimensional PCA to visualize the results.

```
# Load the two-dimensional PCA to scRNA-seq data
sc.pca.ordinal <- readRDS(system.file("extdata", "sc.pca.ordinal.rds", package = "SCI
PAC"))
```

We first generate the input for `ggplot`.

```
rownames(sc.meta.ordinal) <- sc.meta.ordinal$Cell
sc.meta.ordinal <- sc.meta.ordinal[rownames(SCIPAC.res.ordinal), ]
plot.dat <- cbind(sc.meta.ordinal, SCIPAC.res.ordinal)
plot.dat <- cbind(plot.dat, sc.pca.ordinal)

# Assign 0 values of log.pval to those insignificant cells
plot.dat$log.pval.adj <- ifelse((plot.dat$sig == "Not.sig"),
                                0, plot.dat$log.pval)
```

```
# Plot the paths
colors.cell <- tableau_color_pal("Classic 20",
                                 direction = 1)(length(unique(plot.dat$Group)))
p1 <- ggplot(plot.dat) +
  geom_point(aes(x=PC1, y=PC2, col = Group, fill = Group)) +
  scale_color_manual(name = "Differentiation path", values = colors.cell) +
  scale_fill_manual(name = "Differentiation path", values = colors.cell) +
  guides(colour = guide_legend(override.aes = list(size=4))) +
  theme(legend.position= "top",
        axis.text.x = element_blank(),
        axis.text.y = element_blank(),
        panel.background = element_blank(),
        panel.grid = element_line(color = "lightgrey", size = 0.1),
        legend.key = element_blank(),
        legend.background = element_rect(fill = "white", size = 0.1))+
  labs(x = "PC 1", y = "PC 2")

# Plot the steps
plt <- plot.dat %>%
  ggplot(aes(x = PC1, y = PC2, col = Step)) +
  theme_tufte(base_size = 12) +
  theme(panel.background = element_rect(fill = NA, color = "black")) +
  scale_color_gradient2(name = "Differentiation step", low = "blue", high = "red", mi
```

```
d = "white", midpoint = 1000) +
  theme(plot.title = element_text(hjust = 0.5, family = "sans"),
        legend.key.size = unit(1, "cm"),
        legend.position= "top",
        panel.background = element_blank(),
        panel.grid = element_line(color = "lightgrey", size = 0.1),
        axis.text.x = element_blank(),
        axis.text.y = element_blank()) +
  labs(x = "PC 1", y = "PC 2")
p2 <- plt + geom_point()

# Control the range of the estimate of Lambda.Psi within -2 and 2
plot.dat$Lambda.adj <- plot.dat$Lambda.est
if(sum(plot.dat$Lambda.est <= -2) > 0){
  plot.dat[which(plot.dat$Lambda.est <= -2), ]$Lambda.adj <- -2
}
if(sum(plot.dat$Lambda.est >= 2) > 0){
  plot.dat[which(plot.dat$Lambda.est >= 2), ]$Lambda.adj <- 2
}

p3 <- ggplot(plot.dat) +
  geom_point(aes(x=PC1, y=PC2, col=Lambda.adj)) +
  scale_color_gradient2(expression(Lambda), low="darkblue",high='red', mid = "white",
midpoint = 0,
                        limits = c(-2, 2), breaks = c(-2, -1, 0, 1, 2),
                        labels = c(expression(phantom(x) <= -2), -1, 0, 1, expression
(phantom(x) >= 2))) +
  theme(legend.title = element_text(family = "sans", size = 27),
        legend.position= "top",
        axis.text.x = element_blank(),
        axis.text.y = element_blank(),
        panel.background = element_blank(),
        panel.grid = element_line(color = "lightgrey", size = 0.1))+
  labs(x = "PC 1", y = "PC 2")

# Control the range of log.pval.adj within -3 and 3
plot.dat$pval.adj.adj <- plot.dat$log.pval.adj
if(sum(plot.dat$log.pval.adj <= -3) > 0){
  plot.dat[which(plot.dat$log.pval.adj <= -3), ]$pval.adj.adj <- -3
}
if(sum(plot.dat$log.pval.adj >= 3) > 0){
  plot.dat[which(plot.dat$log.pval.adj >= 3), ]$pval.adj.adj <- 3
}

plot.dat.positive <- plot.dat[which(plot.dat$pval.adj.adj > 0), ]
plot.dat.positive$pval.adj.adj <- -plot.dat.positive$pval.adj.adj
```

```
plot.dat.negative <- plot.dat[which(plot.dat$log.pval.adj <= 0), ]

p4 <- ggplot() +
  geom_point(data = plot.dat.positive, aes(x=PC1, y=PC2, col=pval.adj.adj)) +
  scale_color_gradient2("p-values", low="red",high='white', mid = "white", midpoint = 
0,
                        limits = c(-3, -1.3), breaks = c(-3, -2, -1.3),
                        labels = c(expression(phantom(x) <= 0.001), 0.01, 0.05),
                        na.value = "white") +
  new_scale_color() +
  geom_point(data = plot.dat.negative, aes(x=PC1, y=PC2, col=pval.adj.adj)) +
  scale_color_gradient2('    ', low="darkblue",high='white', mid = "white", midpoint = 
0,
                        limits = c(-3, -1.3), breaks = c(-3, -2, -1.3),
                        labels = c(expression(phantom(x) <= 0.001), 0.01, 0.05),
                        na.value = "white") +
  theme(legend.position= "top",
        axis.text.x = element_blank(),
        axis.text.y = element_blank(),
        panel.background = element_blank(),
        panel.grid = element_line(color = "lightgrey", size = 0.1))+
  labs(x = "PC 1", y = "PC 2")
```

```
cowplot::plot_grid(p1, p2, p3, p4, nrow = 2)
```

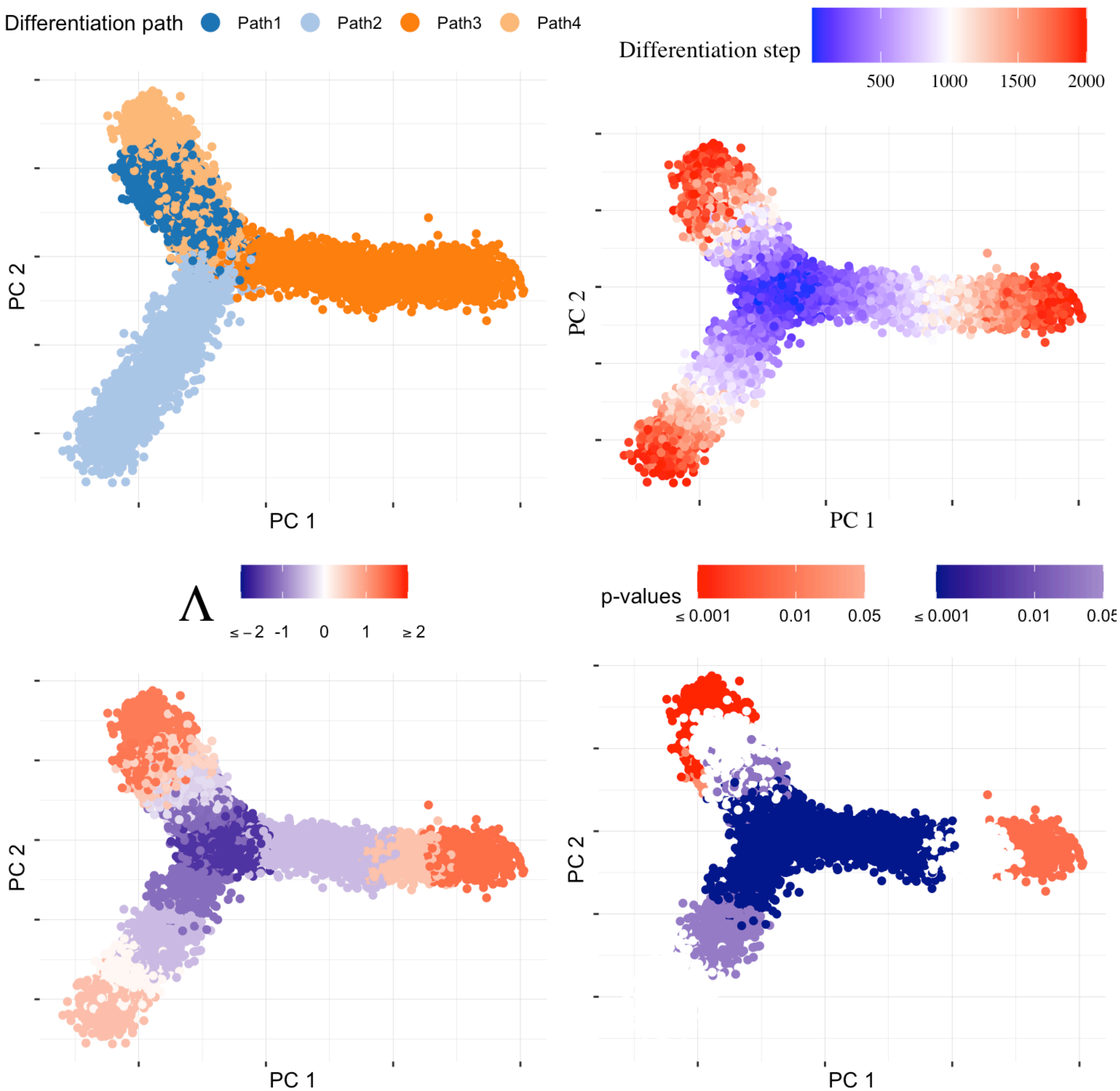

Applying SCIPAC to data with survival information

In this section, we use the Lung Adenocarcinoma (LUAD) scRNA-seq data to show how to use SCIPAC in real applications with survival information. This LUAD scRNA-seq data is obtained from the following paper (https://www.nature.com/articles/s41591-018-0096-5). For convenience, we put the pre-processed and

dimension-reduced scRNA-seq data in the R package with its corresponding meta data, as loaded below.

```
# Load the pre-processed and dimension-reduced LUAD scRNA-seq data
LUAD.sc.dat <- readRDS(system.file("extdata", "LUAD.sc.dat.rds", package = "SCIPAC"))
# Load the meta data for LUAD scRNA-seq data
LUAD.sc.meta <- readRDS(system.file("extdata", "LUAD.meta.rds", package = "SCIPAC"))
```

Let's check the first few rows of the meta data.

```
head(LUAD.sc.meta)
```

	cellID <chr>	celltype <chr>	patient_num <chr>
AAACCTGAGTAACCCT_13	AAACCTGAGTAACCCT_13	B cells	3
AAACCTGCAACGATGG_13	AAACCTGCAACGATGG_13	Macrophages	3
AAACCTGCATTAGCCA_13	AAACCTGCATTAGCCA_13	Macrophages	3
AAACCTGGTCCATGAT_13	AAACCTGGTCCATGAT_13	CD4+ T	3
AAACCTGTCCTGTACC_13	AAACCTGTCCTGTACC_13	Macrophages	3
AAACGGGCAAGAAAGG_13	AAACGGGCAAGAAAGG_13	Macrophages	3

6 rows

Working with TCGA database

To obtain the bulk RNA-seq data and the corresponding clinical information, we use *TCGAbiolinks* R package. For more information about this package, users can refer to the TCGAbiolinks tutorial (https://bioconductor.org/packages/release/bioc/html/TCGAbiolinks.html). Apart from using TCGA database, users can also use cBioPortal (https://www.cbioportal.org) to download bulk RNA-seq data of interests. In the following steps, we provide the steps of obtaining the bulk RNA-seq data and its corresponding clinical information for LUAD patients.

Considering that it may take a while to download the bulk RNA-seq data and the clinical information, we only provide the code without running it.

```
# library(TCGAbiolinks)
# library(SummarizedExperiment)
### Obtain patient and count data information
# query <- GDCquery(
#   project = "TCGA-LUAD", # The project name used. We choose the TCGA-LUAD data
#   data.category = "Gene expression",
#   data.type = "Gene expression quantification",
#   platform = "Illumina HiSeq",
#   file.type  = "normalized_results", # We use the normalized bulk RNA-seq data
#   experimental.strategy = "RNA-Seq",
#   legacy = TRUE
# )
#
### Upload data from GDC and save to "GDCdata_LUAD"
# GDCdownload(
#   query = query,
#   method = "api",
#   files.per.chunk = 10,
#   directory = "GDCdata_LUAD" # Saved path
# )
#
#
# bulk.dat <- GDCprepare(query = query, directory = "GDCdata_LUAD")
#
# clinical.info <- as.data.frame(colData(bulk.dat))
#
# exprs.dat <- assay(bulk.dat)
```

The downloaded bulk RNA-seq expression data is in `exprs.dat` and the clinical information is in `clinical.info`. For convenience, we provide the pre-processed and dimension-reduced bulk RNA-seq data and its meta data, as loaded below.

```
library(dplyr)
# Load the pre-processed and dimension-reduced LUAD bulk RNA-seq data
LUAD.bulk.dat <- readRDS(system.file("extdata", "LUAD.bulk.dat.rds", package = "SCIPA
C"))
# Load the meta data for LUAD bulk RNA-seq data
LUAD.bulk.meta <- readRDS(system.file("extdata", "LUAD.bulk.meta.rds", package = "SCI
PAC")) %>% as.data.frame()
```

Let's check the first few rows of the meta data.

```
head(LUAD.bulk.meta)
```

	patient	definition

	<chr>	<chr>
TCGA-64-5781-01A-01R-1628-07	TCGA-64-5781	Primary solid Tumor
TCGA-49-4490-01A-21R-1858-07	TCGA-49-4490	Primary solid Tumor
TCGA-78-8660-01A-11R-2403-07	TCGA-78-8660	Primary solid Tumor
TCGA-78-7143-01A-11R-2039-07	TCGA-78-7143	Primary solid Tumor
TCGA-44-3398-01A-01R-1107-07	TCGA-44-3398	Primary solid Tumor
TCGA-91-6828-01A-11R-1858-07	TCGA-91-6828	Primary solid Tumor

6 rows | 1-3 of 9 columns

The survival information is contained in the three columns `LUAD.bulk.meta$vital_status` , `LUAD.bulk.meta$days_to_last_follow_up` , and `LUAD.bulk.meta$days_to_death` . We further make some adjustment to obtain the input `y` .

```
LUAD.bulk.meta$S_time <- ifelse(is.na(LUAD.bulk.meta$days_to_death),
                               LUAD.bulk.meta$days_to_last_follow_up,
                               LUAD.bulk.meta$days_to_death)
LUAD.bulk.meta$S_status <- ifelse(LUAD.bulk.meta$vital_status == "Dead", 1, 0)

all(rownames(LUAD.bulk.meta) == rownames(LUAD.bulk.dat))
```

```
## [1] TRUE
```

```
# Delete normal cases
# Delete NAs in survival time
# Delete S_time <= 0
normal.idx <- which(LUAD.bulk.meta$sample_type == "Solid Tissue Normal")
na.idx <- which(is.na(LUAD.bulk.meta$S_time))
leq.zero.idx <- which(LUAD.bulk.meta$S_time <= 0)

LUAD.bulk.meta.sub <- LUAD.bulk.meta[-c(normal.idx, na.idx, leq.zero.idx), ]


LUAD.bulk.dat.sub <- LUAD.bulk.dat[rownames(LUAD.bulk.meta.sub), ]
y <- LUAD.bulk.meta.sub[, c("S_time", "S_status")] %>% as.matrix()
colnames(y) <- c("time", "status")
all(rownames(LUAD.bulk.dat.sub) == rownames(y))
```

```
## [1] TRUE
```

Clustering

As before, we first perform clustering on the scRNA-seq data

```
LUAD.ct.res <- seurat.ct(LUAD.sc.dat, res = 2.0)
```

```
## Computing nearest neighbor graph
```

```
## Computing SNN
```

```
## Modularity Optimizer version 1.3.0 by Ludo Waltman and Nees Jan van Eck
##
## Number of nodes: 29477
## Number of edges: 1332182
##
## Running Louvain algorithm...
## Maximum modularity in 10 random starts: 0.8547
## Number of communities: 43
## Elapsed time: 5 seconds
```

Executing SCIPAC

After all the preparation, we can finally run SCIPAC.

```
SCIPAC.res.LUAD <- SCIPAC(bulk.dat = LUAD.bulk.dat.sub, y = y,
                             family = "cox", ct.res = LUAD.ct.res,
                             ela.net.alpha = 0.4, bt.size = 50,
                             numCores = 7, CI.alpha = 0.05, nfold = 10)
```

We set `family = "cox"` for survival input `y` and use the default values for other parameters.

Visualization of results

Let's view the results generated by SCIPAC. For convenience, we load a two-dimensional UMAP data to visualize the results.

```
LUAD.sc.umap <- readRDS(system.file("extdata", "LUAD.sc.umap.rds", package = "SCIPA
C"))
```

We first generate the input for `ggplot`.

```
LUAD.sc.meta <- LUAD.sc.meta[rownames(SCIPAC.res.LUAD), ]
plot.dat <- cbind(LUAD.sc.meta, SCIPAC.res.LUAD)
all(rownames(plot.dat) == rownames(LUAD.sc.umap))
```

```
## [1] TRUE
```

```
plot.dat <- cbind(plot.dat, LUAD.sc.umap)
plot.dat$log.pval.adj <- ifelse((plot.dat$sig == "Not.sig"),
                                0, plot.dat$log.pval)
```

```
# Plot the cell types
colors.cell <- tableau_color_pal("Classic 20",
                                 direction = 1)(length(unique(plot.dat$celltype)))
plt <- plot.dat %>%
  ggplot(aes(x = umap.X1, y = umap.X2, col = celltype, fill = celltype)) +
  theme_tufte(base_size = 12) +
  theme(panel.background = element_rect(fill = NA, color = "black")) +
  guides(color = guide_legend(override.aes = list(stroke = 1,
                                                  alpha = 1, shape = 16, size = 4)),
         alpha = FALSE) +
  scale_color_manual(" ", values = colors.cell, guide = "none") +
  scale_fill_manual(" ", values = colors.cell, guide = "none") +
  theme(legend.position= "top") +
  labs(x = "UMAP 1", y = "UMAP 2")
```

```
## Warning: `guides(<scale> = FALSE)` is deprecated. Please use `guides(<scale> =
## "none")` instead.
```

```
p1 <- plt + geom_point()

# Control the range of the estimate of Lambda.Psi within -2 and 2
plot.dat$Lambda.adj <- plot.dat$Lambda.est
if(sum(plot.dat$Lambda.est <= -2) > 0){
  plot.dat[which(plot.dat$Lambda.est <= -2), ]$Lambda.adj <- -2
}
if(sum(plot.dat$Lambda.est >= 2) > 0){
  plot.dat[which(plot.dat$Lambda.est >= 2), ]$Lambda.adj <- 2
}

p2 <- ggplot(plot.dat) +
  geom_point(aes(x=umap.X1, y=umap.X2, col=Lambda.adj)) +
  scale_color_gradient2(expression(Lambda), low="darkblue",high='red', mid = "white",
midpoint = 0,
```

```
                        limits = c(-2, 2), breaks = c(-2, -1, 0, 1, 2),
                        labels = c(expression(phantom(x) <= -2), -1, 0, 1, expression
(phantom(x) >= 2))) +
  theme(legend.title = element_text(family = "sans", size = 27),
        legend.position= "top",
        axis.text.x = element_blank(),
        axis.text.y = element_blank(),
        panel.background = element_blank(),
        panel.grid = element_line(color = "lightgrey", size = 0.1))+
  labs(x = "UMAP 1", y = "UMAP 2")

# Control the range of log.pval.adj within -3 and 3
plot.dat$pval.adj.adj <- plot.dat$log.pval.adj
if(sum(plot.dat$log.pval.adj <= -3) > 0){
  plot.dat[which(plot.dat$log.pval.adj <= -3), ]$pval.adj.adj <- -3
}
if(sum(plot.dat$log.pval.adj >= 3) > 0){
  plot.dat[which(plot.dat$log.pval.adj >= 3), ]$pval.adj.adj <- 3
}

plot.dat.positive <- plot.dat[which(plot.dat$pval.adj.adj > 0), ]
plot.dat.positive$pval.adj.adj <- -plot.dat.positive$pval.adj.adj

plot.dat.negative <- plot.dat[which(plot.dat$log.pval.adj <= 0), ]

p3 <- ggplot() +
  geom_point(data = plot.dat.negative, aes(x=umap.X1, y=umap.X2, col=pval.adj.adj)) +
  scale_color_gradient2('   ', low="darkblue",high='white', mid = "white", midpoint =
0,
                        limits = c(-3, -1.3), breaks = c(-3, -2, -1.3),
                        labels = c(expression(phantom(x) <= 0.001), 0.01, 0.05),
                        na.value = "white") +
  new_scale_color() +
  geom_point(data = plot.dat.positive, aes(x=umap.X1, y=umap.X2, col=pval.adj.adj)) +
  scale_color_gradient2("p-values", low="red",high='white', mid = "white", midpoint =
0,
                        limits = c(-3, -1.3), breaks = c(-3, -2, -1.3),
                        labels = c(expression(phantom(x) <= 0.001), 0.01, 0.05),
                        na.value = "white") +
  theme(legend.position= "top",
        axis.text.x = element_blank(),
        axis.text.y = element_blank(),
        panel.background = element_blank(),
        panel.grid = element_line(color = "lightgrey", size = 0.1))+
  labs(x = "UMAP 1", y = "UMAP 2")
```
