# Supplementary material for: SCIPAC: quantitative estimation of cell-phenotype associations
Source: Genome Biol. 2024 May 13;25:119. doi: 10.1186/s13059-024-03263-1 (PMC11089691; doi:10.1186/s13059-024-03263-1)
Supplement: Supplementary file 3 — Additional file 3. Review history. [file 13059_2024_3263_MOESM3_ESM.docx]

Review History

**First round of review**

**Reviewer 1**

**Were you able to assess all statistics in the manuscript, including the appropriateness of statistical tests used?**

**Yes:** The statistical tests are appropriate.

**Were you able to directly test the methods?**

Yes

**Comments to author:**

Gan et al. develops a method named SCIPAC to quantitatively estimate the association between each cell in single-cell data and a phenotype. Quantifying cell type and phenotype associations is an important topic and the majority contents of the manuscript are clearly written. However, it's not quite clear how people are expected to interpret the so-called association between a single cell and a phenotype. In addition, the current results are not sufficiently convincing to show that SCIPAC is better than naïve approaches which directly calculate the associations between cell type/cluster proportions and phenotypes.

1. The definition of association used by the proposed method is confusing. According to the new definition, a cell is positively/negatively associated with a phenotype if increasing the proportion of cells that have similar expression profiles as this cell in the tissue will likely indicate an increased probability for the phenotype to be present. It is counterintuitive that the association between a cell and a phenotype would depend on other cells. Would it be more straightforward to directly study the association between a cell type and a phenotype? The advantage of focusing on individual cells in not clear in this association study. In addition, according to the Methods section, the association seems to be calculated for a cell type instead of a single cell, so I don't understand how the scores were obtained for individual cells.

2. The method is derived assuming that the proportion of a single cell type is changing. What if the proportions of multiple cell types are simultaneously changing? Does the derived result still hold?

3. A more straightforward solution to this problem, as mentioned in the manuscript, is to perform deconvolution on the bulk samples, and then directly study the associations between estimated cell type proportions and phenotypes. However, the proposed SCIPAC method was not compared with this method in the manuscript.

4. In the simulation study, the true association strength is not provided. How does this strength affect the performance of SCIPAC?

5. How robust is the proposed method to the selection of bulk samples. For example, if a different bulk dataset is used in the prostate cancer application, would SCIPAC obtain similar association strengths for the single cells?

Other comments:

Are all genes used in the regression analysis? Is any filtering or feature selection performed?

Why alpha is set to 0.4 in the elastic net model?

Why the resolution parameter in Seurat is set to 2.0, which is much larger than the default value?

Figure 1b-d are confusing. How is the association illustrated? What are the x and y axes?

**Reviewer 2**

**Were you able to assess all statistics in the manuscript, including the appropriateness of statistical tests used?**

**Yes:** I am not an expert for simulated datasets and would suggest that another reviewer comments more on this part.

**Were you able to directly test the methods?**

No

**Comments to author:**

The paper presented by Gan and colleagues describes SCIPAC, a novel solution for the clinical interpretation of scRNA-seq datasets for cancer and beyond. At its core, SCIPAC combines scRNA-seq with bulk RNA-seq data to associate cells and cell clusters to phenotypes, such as cancer, cancer stage and clinical outcome. Interpreting scRNA-seq data in the clinical context is a challenging task and SCIPAC provides a framework for the straightforward association of cell types and states with clinical features. However, while the authors provide both simulated and experimental application examples, the validation of the pipeline lacks depth and the integration of disease expertise to support the conclusion. In its current form, the manuscript is too superficial in its effort the evaluate the performance of the SCIPAC.

Major comments:
Estimating the value of the prediction is difficult due to the lack of a ground truth. The authors argue that the algorithm correctly identifies myeloid cell types in prostate cancer samples that positively or negatively associate with cancer using general assumptions about DC and macrophage cell types and states. It would be more convincing if the authors would choose a single-cell datasets for which both normal and cancer tissue is available, with the normal samples serving as ground truth for negatively associated cell types. A similar scenario for B cells. If B cells infiltration and the formation of TLS is cancer-specific, shouldn't B cells be positively associated with cancer as predicted by Scissors? To my understanding a positive association signals cancer-specificity.

For predicting the association of cancer stages in breast cancer cases, it would be most helpful to include cancer samples with annotated stages so that one can validate the predicted stage of the cancer cell cluster. In its current form, the cancer cells are associated with progression, which should only be true for last stage cancer samples, but not early stage cancers.

The interpretation of cell states associated with cancer progression would greatly benefit from the initial comprehensive annotation of clusters. It is not clear why the authors work on not annotated datasets and rely on DE analysis of clusters for the interpretation and validation of the findings. This would be most important for the MKI67+ cluster, which clearly harbors cycling T cells. Here, the cycling signature will obscure cell type gene expression signatures and mix Treg, but also cytotoxic and exhausted T cell into a single cluster. Again, an appropriate fine-grained annotation based on a strong immunological foundation would be most helpful to better evaluate the performance of the algorithm. The same holds true for the lung cancer samples. CD4+ cells are often Treg cells, which should be associated with poorer patient outcome as they relate to a more immunosuppressive tumor microenvironments. Again, a better annotation would be key to interpret the results.

Minor comments:
Much of the main text can be moved to figure legends. Also, the text can be shortened by moving parts to the Methods, specifically for the simulation section and benchmarking against Scissors.

**Reviewer 3**

**Were you able to assess all statistics in the manuscript, including the appropriateness of statistical tests used?**

No

**Were you able to directly test the methods?**

No

**Comments to author:**

In this manuscript, Gan and colleagues present a computational method called SCIPAC, which associates single-cell transcriptomes with specific phenotypes by integrating bulk and single-cell RNAseq data. The authors demonstrate the method using various types of phenotypes, including binary, ordinal, and continuous. Although SCIPAC shares similarities with a recently developed tool called Scissor, the authors argue that SCIPAC offers two important advantages: estimating the strength and statistical significance of associations for each cell. While the method appears interesting, I have several major concerns.

1, The comparisons between SCIPAC and Scissor are predominantly qualitative, and in most instances, the agreement appears quite limited. This makes it challenging for readers to assess which method provides more meaningful associations. Have the authors tested SCIPAC on the datasets studied in the Scissor paper? Are there any quantitative metrics that can be used for comparison? Currently, the paper relies heavily on UMAPs to compare the methods and demonstrate the consistency of SCIPAC output under various resolution parameters (e.g., the breast cancer dataset). Quantitative measurements are crucial for making the arguments more convincing.

2, The association analysis is performed on pseudobulked expression profiles based on either pre-annotated cell type identities or Louvain clusters, rather than directly on single cells. This should be accurately stated in the text. Relatedly, would using metacells (e.g., from SEACells) improve the analysis?

3, The calculation of p-values assumes normal distributions of association scores. Is this assumption supported by experimental data? Additionally, the p-value calculation lacks size correction, so running different numbers of tests at various clustering levels could significantly affect the results.

4, The biological meaning of association strength remains ambiguous. How should we interpret the association strength inferred by SCIPAC for binary phenotypes? For ordinal phenotypes, is there a correlation between differentiation stages and the inferred associations (taking figure 2 as an example)?

5, In the breast cancer dataset, why are cancer-associated fibroblasts assigned a stronger association value than the actual malignant cancer epithelial cells? Is this surprising, and does it raise questions about the value or accuracy of the quantitative association strengths?

**Authors Response**

**Point-by-point responses to the reviewers’ comments:**

We thank the reviewers for their very insightful comments and the editor for handling our submission. In response, we have done additional analysis, edited both the manuscript and Supplementary Material, and highlighted changes accordingly. Our detailed responses are as follows.

**Response to Reviewer 1**

Gan et al. develops a method named SCIPAC to quantitatively estimate the association between each cell in single-cell data and a phenotype. Quantifying cell type and phenotype associations is an important topic and the majority contents of the manuscript are clearly written. However, it’s not quite clear how people are expected to interpret the so-called association between a single cell and a phenotype. In addition, the current results are not sufficiently convincing to show that SCIPAC is better than naïve approaches which directly calculate the associations between cell type/cluster proportions and phenotypes.

**Q1** The definition of association used by the proposed method is confusing. According to the new definition, a cell is positively/negatively associated with a phenotype if increasing the proportion of cells that have similar expression profiles as this cell in the tissue will likely indicate an increased probability for the phenotype to be present. It is counterintuitive that the association between a cell and a phenotype would depend on other cells. Would it be more straightforward to directly study the association between a cell type and a phenotype? The advantage of focusing on individual cells in not clear in this association study. In addition, according to the Methods section, the association seems to be calculated for a cell type instead of a single cell, so I don’t understand how the scores were obtained for individual cells.

**A1** We appreciate the reviewer for posing an insightful question, and we apologize for any confusion. The reviewer is correct in pointing out that the association is calculated for a

group of cells rather than for individual cells. Once this calculation is performed, the same association value is assigned to every cell within the group.

In our original definition, we adopted the phrase “cells that have similar expression profiles as this cell” as a proxy for “cells of the same cell type.” This choice of avoiding using “cell type” was intentional and served two purposes. Firstly, our program SCIPAC does not presuppose the knowledge of cell types, but rather clusters the cells in an unsupervised manner by default. Secondly, cells presumed to belong to the same cell type may display varied associations with the phenotype—this variation could be in the direction or strength of association, potentially due to these cells belonging to different, unrecognized sub-types. With these factors in mind, we opted for a more comprehensive definition.

However, we concur with the reviewer’s observation that our initial definition may have been somewhat intricate and could lead to confusion. Therefore, we have updated it as fol­lows: “A group of cells that are likely to play a similar role in the phenotype (such as cells of a specific cell type or sub-type, cells in a particular state, cells in a cluster, cells with similar expression profiles, or cells with similar functions) is considered to be positively/negatively associated with a phenotype if an increase in their proportion within the tissue likely indi­cates an increased/decreased probability of the phenotype’s presence. SCIPAC assigns the same association to all cells within such a group.” We have also updated a few other places for consistency.

Q2 The method is derived assuming that the proportion of a single cell type is changing. What if the proportions of multiple cell types are simultaneously changing? Does the derived result still hold?

A2 We extend our gratitude to the reviewer for raising this insightful question. When in­terpreting statistical models, researchers typically focus on changes in individual influenc­ing variables separately. For instance, in multiple linear regression models, the influence of each income variable is usually discussed by examining the sign and magnitude of its coefficient. Similarly, in more complex machine learning models like gradient boosting ma­chines (GBM, <https://en.wikipedia.org/wiki/Gradient_boosting>), researchers also in­vestigate the nonlinear effect of each income variable.

There are at least two primary reasons why simultaneous changes of multiple variables are rarely considered. Firstly, the derivations can become exceedingly complex or even unattainable when dealing with simultaneous changes in multiple quantities. Secondly, and perhaps more importantly, it can be challenging to summarize or articulate the results. The impact on the outcome often depends on the specific manner in which these multiple

variables change. To illustrate, let’s consider the change in proportions of two cell types. An increase in cell type 1 by three times, relative to an increase in cell type 2, usually yields different effects than an equal increase in both types.

In the case of SCIPAC, deriving the “association strength” under alterations of multiple cell types, given the relative amount of changes in the proportion of each of these cell types, is not overly complicated. However, interpreting this association strength becomes signifi­cantly challenging. It becomes difficult to ascertain how two cell types together associate with the phenotype, as the association now depends on the extent to which each cell type increases. Furthermore, visualizing the association also becomes nearly impossible. In the current version of SCIPAC, which considers single-cell-type changes, we can easily display the association strength as the shade of the red/blue color in a scatterplot of all cells, be­cause each cell/cell type is assigned a single value of the association strength. However, this approach does not apply to the display of association strength of multiple cell types, as each cell type is assigned to (infinitely) many association strengths, depending on which other cell type is considered and how both cell types change.

Due to these reasons, we have decided to abstain from extending the association strength to simultaneous changes of multiple cell types, as this concept could be difficult to use in real-world applications.

Q3 A more straightforward solution to this problem, as mentioned in the manuscript, is to perform deconvolution on the bulk samples, and then directly study the associations between estimated cell type proportions and phenotypes. However, the proposed SCIPAC method was not compared with this method in the manuscript.

A3 We appreciate the reviewer’s insights. However, we believe there may be some misun­derstanding here. We mentioned deconvolution when discussing Equations (1) and (2) in the main text. Our intention was to highlight that while we assume the bulk data can be deconvoluted, executing the deconvolution process is not necessary to obtain Equation (2). We would like to clarify further that even if deconvolution is performed and the compo­sition coefficients (*γk* for *k = 1, . . . , K*) are obtained, these coefficients do not aid in the computation of G^*^. The G^*^ computed using these coefficients will still be identical to that computed using Equation (2), which does not rely on the coefficients. Additionally, Equa­tions (1) and (2) constitute only a small part of the SCIPAC algorithm. When developing SCIPAC, we did not anticipate or realize the existence of a “more straightforward” solution based on deconvolution.

Nevertheless, the reviewer has raised a valid point by suggesting a two-step deconvolution-

based approach that involves deconvoluting each bulk sample and comparing the differences in proportions of each cell type between samples with different phenotypes (e.g., cancer vs. normal). However, we would like to argue that this approach does not address the same problem as SCIPAC. The deconvolution-based approach tests each cell type separately and independently, whereas SCIPAC (implicitly) utilizes the compositions of all cell types si­multaneously to construct a classifier, regression model, or survival model. The difference between them is somewhat similar to the distinction between many single-variable linear regression models, each considering the relationship between one input variable and the outcome, versus a multi-variable linear regression model that considers the relationship between all input variables and the outcome. Thus, we believe SCIPAC provides a more comprehensive solution.

Despite the conceptual advantages of SCIPAC, we have decided to follow the reviewer’s suggestion and do the comparison. We implemented the deconvolution-based approach and applied it to our simulation data 1. We deconvoluted the bulk samples using MuSiC[1], which is one of the most popular reference-based deconvolution methods [2], to obtain the cell type proportions for each bulk sample. Subsequently, we performed a two-sample t-test on each cell type to determine whether the proportions differed between the two sample groups. The results are presented in Figure RL1. It is worth noting that although this deconvolution-based strategy correctly captures the truly positive/negative-associated cells in the upper right corner of the plots, it yields an unacceptably large number of false positive discoveries. Notably, a significant proportion of cells from cell type 3 (the largest cluster of cells, which should be null) are falsely identified as significant.

This outcome is not entirely unexpected. Firstly, deconvolution methods typically tackle a constrained optimization problem, where one constraint requires the proportions of all cell types to be non-negative, and another constraint requires that the proportions sum up to 1. These constraints are likely to introduce biases in the solutions and truncate the distribu­tions of each proportion. Consequently, the subsequent two-sample tests are compromised. Secondly, in the deconvolution-then-test strategy, the proportions of cell types estimated in the deconvolution step have variances. However, these estimation variances are disregarded in the second step, where the proportions are treated as actual/observed values to compute the t-statistic. As a result, these t-statistics become inflated, leading to a significant number of false positive discoveries.

We have added this discussion about deconvolution to the Supplementary Material.

Q4 In the simulation study, the true association strength is not provided. How does this

Figure RL1: UMAP visualization of the simulated data under scheme I using the deconvolution-then-test strategy. (a) mean cell proportion differences between two sam­ple groups. (b) p-values of the mean cell proportion differences using two sample t-test. It is noticeable that while this strategy can capture the truly positive/negative associated cells in the upper right corner of the plots, there is still an unacceptably large number of false positive discoveries.

strength affect the performance of SCIPAC?

A4 We appreciate the reviewer for posing this insightful question. Given that the data is sim­ulated using a single-cell expression simulator, it is not possible to provide a quantitative value for the “true” association strength that directly corresponds to the association strength defined by SCIPAC. For instance, in our simulation scheme III, the true association strength arises from the differentiation step. In this case, the association strength has a more relative interpretation. It can be compared to zero, indicating a positive/negative association. It can also be compared to the association strengths of other cell types, where a larger associa­tion strength for a cell type compared to another suggests a stronger association with the phenotype.

Our simulation scheme III in the main text demonstrates that SCIPAC can accurately estimate the (relative) association strength. Figure 2i illustrates the true (relative) associa­tion strength based on how we generated the data, while Figure 2j presents the association strength estimated by SCIPAC. Although the absolute values of association strengths in Fig­ure 2i and Figure 2j are not directly comparable, the relative values, indicating which cells have larger or smaller values, exhibit good agreement.

Moreover, we extended our analysis to modify simulation scheme I in order to examine how the strength of association impacts the performance of SCIPAC. Our modified simulation follows these steps:

1. Generate single-cell expression using the same approach as described in the manuscript.
2. Generate 100 random numbers a, ∼ Uniform(−0.5, 0.5), for i = 1, 2, . .. , 100.
3. Generate pseudo-bulk samples by mixing 1 − a, proportion of cells from cell type 3 with a, proportion of cells from cell type 1 (when a, > 0) or cell type 2 (when a, < 0), where • represents the absolute value.
4. Generate the phenotype label y, using a Bernoulli(exp^ait^ /(1 + exp^ait^)) distribution.

Within this simulation scheme, the parameter t serves as a proxy for the true association strength between the phenotype and cell types 1 & 2: for a sample with a fixed positive/neg-ative a, value, a larger value of t signifies a more definitive categorization as cancer/normal. We provide the estimated association strengths and p-values for different t values in Figures RL2 and RL3, respectively. As observed, as t increases, the estimated association becomes increasingly significant.

We have included this new experiment in Supplementary Material.

Q5 How robust is the proposed method to the selection of bulk samples. For example, if a different bulk dataset is used in the prostate cancer application, would SCIPAC obtain similar association strengths for the single cells?

A5 Thanks to the reviewer for posing this valuable question. In response to the reviewer’s guidance, we diligently searched for alternative bulk prostate cancer datasets, apart from the TCGA data that we initially used. We explored various databases such as cBioportal, EMBL-EBI data sources, and the GEO data repository. Unfortunately, we encountered difficulties in finding suitable prostate cancer bulk data due to the following reasons.

Firstly, we required bulk data that examined prostate cancer in a general context. How­ever, many studies have specific designs and objectives, such as gene knockout experiments, different treatment approaches, or investigations into specific gene mutations. Using bulk data from studies that focus only on a particular aspect of prostate cancer would give biased results.

Secondly, certain studies did not include normal control samples, as they solely focused on cancer tissues. On such data, SCIPAC cannot be executed.

Figure RL2: UMAP visualization of the simulated data under scheme I with the estimated association strength Λ, using different values oft, the proxy for the true association strength.

To overcome this challenge while still examining the robustness of SCIPAC, we divided the TCGA-PRAD bulk data used in our paper into two separate parts based on their collection years. Each part was treated as an independent (bulk) RNA-seq dataset. Specifically, the bulk samples were collected between the years 2000 and 2013. We allocated the samples collected in the last three years to part 1, and samples collected in other years to part 2. By doing so, we ensured that both parts contained a similar number of samples.

The association strengths estimated by SCIPAC on these two divided parts are presented in Figure RL4 a and c, respectively, while the corresponding p-values are shown in Figure RL4 b and d. Despite some minor discrepancies, we observed that both the estimated associ­ation strengths and p-values were largely similar between the two parts. This indicates that SCIPAC demonstrates reasonable robustness when it comes to the selection of bulk samples.

We have included this additional analysis in Supplementary Material and discussed it in the Discussion section of the main text.

Figure RL3: UMAP visualization of the simulated data under scheme I with the p values of the estimated association strength Λ, using different values oft, the proxy for the true association strength. As t increases, the estimated association strength becomes increasingly significant.

Q6 Are all genes used in the regression analysis? Is any filtering or feature selection per­formed?

A6 Not all genes are used in the analysis. We follow the standard protocol for single-cell RNA-seq data pre-processing and only keep the 1,000 high variable genes for further analy­sis. For details about the pre-processing of the data, please refer to section 2 of Supplemen­tary Material.

Q7 Why alpha is set to 0.4 in the elastic net model?

A7 The elastic net model has two tuning parameters, λ and α. λ controls the overall penalty strength, while α determines the mixing proportions of the £1 and £2 penalties. Tuning these two parameters can be time-consuming, and people have found that the choice of λ is typically more critical than that of α. Consequently, we opted to fix the α value (although

the SCIPAC R package allows users to use non-default α values) and only tune λ. Through our experiments, we found that α = 0.4 performed well across all scenarios. We have also found that SCIPAC demonstrated limited sensitivity to the choice of α. Figure RL5 illustrates the results of SCIPAC under three different α values: 0.3, 0.4, and 0.5, which agree well with each other.

We appreciate the reviewer’s question regarding the choice of α, and we have incorpo­rated the above discussions into the Discussion section of our main text.

**Q8** Why the resolution parameter in Seurat is set to 2.0, which is much larger than the default value?

**A8** Thanks to the reviewer for raising this insightful question. The reason SCIPAC sets a larger-than-default resolution parameter compared to Seurat is due to the different aims of the two algorithms. Seurat’s default setting is primarily designed for cell type annota­tion. By setting the resolution parameter to 0.5, Seurat can effectively identify major cell populations such as immune cells, myeloid cells, and cancer cells. This default resolution parameter enables researchers to easily distinguish these major cell populations. However, it is important to note that a major cell type can actually consist of multiple subtypes, each with its own distinct biological function and association strength to the phenotype. In such cases, using a larger-than-default resolution parameter becomes advantageous. This allows for the identification of potential subtypes within the major cell type and enables the estima­tion of individual association strengths. Consequently, a more detailed and comprehensive description of the association between single cells and the phenotype can be obtained by SCIPAC.

We have added this discussion to the Discussion section of the main text.

**Q9** Figure 1b-d are confusing. How is the association illustrated? What are the x and y axes?

**A9** Sorry for the confusion. The scatterplots in Figure 1b-d depict the dimension-reduced data given by UMAP, where the x and y axes represent the two dimensions of the dimension-reduced data. We intentionally removed the scales for the x- and y-axes since the low-dimensional space is primarily used for visualization purposes, and the specific values on these axes may not be directly relevant. The meaning of the colors in the scatterplots is provided in the legend of Figure 1, and detailed instructions on how to interpret the figure can be found in subsection “Simulation scheme I” of the section “Performance in simulated data.” We have added more instructions on how to read the figure to the figure legend.

**Response to Reviewer 2**

The paper presented by Gan and colleagues describes SCIPAC, a novel solution for the clini­cal interpretation of scRNA-seq datasets for cancer and beyond. At its core, SCIPAC combines scRNA-seq with bulk RNA-seq data to associate cells and cell clusters to phenotypes, such as cancer, cancer stage and clinical outcome. Interpreting scRNA-seq data in the clinical context is a challenging task and SCIPAC provides a framework for the straightforward as­sociation of cell types and states with clinical features. However, while the authors provide both simulated and experimental application examples, the validation of the pipeline lacks depth and the integration of disease expertise to support the conclusion. In its current form, the manuscript is too superficial in its effort the evaluate the performance of the SCIPAC.

**Response to the overall impression** We greatly appreciate the expertise of the reviewer in the field of biology and disease, and we find the comments and suggestions to be highly con­structive. It is important to note that this project was primarily designed as a methodological study, focusing on the novelty and efficiency of computational methods, rather than on pre­senting new biological or medical findings. As such, we have submitted our manuscript to Genome Biology as a “Method” paper, in accordance with the journal’s guidelines ([https:// genomebiology.biomedcentral.com/submission-guidelines/preparing-your-manuscr](https://genomebiology.biomedcentral.com/submission-guidelines/preparing-your-manuscript/method)ipt/ [method](https://genomebiology.biomedcentral.com/submission-guidelines/preparing-your-manuscript/method)), which states that “For computational methods, demonstration of superiority should be carried out using the same dataset. Where possible, the method should be benchmarked using a synthetic dataset (or other dataset where the ground truth is known), and its utility on real data demonstrated. A Method article need not necessarily provide novel biological insights, but these can help to demonstrate the method’s utility.”

Nevertheless, we fully acknowledge the viewpoint that incorporating in-depth biologi­cal interpretation into the results is crucial and can significantly enhance the value of our methodological work. To address this, we have collaborated closely with two experts in the field of cancer, specifically an associate professor and a PhD specializing in molecular biology and immunotherapy of metastatic cancer. These experts are co-authors of our paper and have actively contributed to the interpretation and evaluation of the SCIPAC results. De­spite these efforts, we concur with the reviewer that the current evaluation on real data may still be perceived as superficial by many experts in the field, particularly when compared to publications focused on biology and discovery in specialized journals.

**Q1** Estimating the value of the prediction is difficult due to the lack of a ground truth. The authors argue that the algorithm correctly identifies myeloid cell types in prostate cancer samples that positively or negatively associate with cancer using general assumptions about

DC and macrophage cell types and states. It would be more convincing if the authors would choose a single-cell datasets for which both normal and cancer tissue is available, with the normal samples serving as ground truth for negatively associated cell types. A similar sce­nario for B cells. If B cells infiltration and the formation of TLS is cancer-specific, shouldn’t B cells be positively associated with cancer as predicted by Scissors? To my understanding a positive association signals cancer-specificity.

A1 We appreciate the reviewer’s valuable insights and constructive suggestions. The single-cell prostate cancer data we used in our analysis has both normal and cancer tissues. Follow­ing the reviewer’s suggestion, we add an analysis using the single-cell data from the normal prostate tissue and the same bulk data. In Figure RL6, the first row shows the results for the cancer tissue, and the second row shows the results for the normal tissue. If we treat normal samples as the ground truth, it becomes apparent that the association strength and significance are both enhanced in the cancer tissue. For instance, in the normal tissue, the MNP and B cell populations exhibit a negative association with cancer; however, these as­sociations are not statistically significant due to their relatively weak strengths. Conversely, in the cancer tissue, both cell populations continue to display a negative association, but with significantly stronger association strengths and statistical significance. This suggests that in the cancer tissue, the tumor-inhibiting functions of MNP and B cells become more pronounced and noteworthy.

We agree that the B cell infiltration and the formation of TLS could be cancer-specific, as many studies have suggested [3, 4]. However, being cancer-specific does not necessarily imply a cell type is cancer-associated. In the context of SCIPAC, a positive association of a cell type means that increasing this cell type will lead to increasing the probability of the presence of a phenotype. Mathematically, increasing a cancer-specific cell type may dilute the concentrations of cell types that have even stronger associations with the phenotype, and hence decrease the probability of the presence of cancer. Biologically, cell types found exclusively in cancer tissues encompass both cells that initiate or promote cancer develop­ment and cells employed by the immune system to combat cancer. Thus, cancer-specific cell types can exhibit either positive or negative associations with cancer, according to SCIPAC’s definition of phenotype association. In fact, accumulating research has indicated that there is a close association between tumor-associated TLSs and better prognosis, as well as favor­able clinical outcomes following immunotherapy [3, 4, 5], which align with our analysis.

We have included our discussion on the relationship between being cancer-specific and cancer-associated in Supplementary Material.

Q2 For predicting the association of cancer stages in breast cancer cases, it would be most helpful to include cancer samples with annotated stages so that one can validate the pre­dicted stage of the cancer cell cluster. In its current form, the cancer cells are associated with progression, which should only be true for last stage cancer samples, but not early stage cancers.

A2 We greatly appreciate the reviewer’s advice. We fully concur that including single-cell cancer samples with annotated stages can help validate the predicted stage of cancer cell clusters. It would be interesting to see whether the stages predicted by SCIPAC align with the ground truth. Despite our attempts to find breast cancer single-cell data with clearly annotated cancer stages, we were unable to find any. This scarcity may be attributed to the high cost associated with conducting single-cell sequencing on multiple patient tissues.

Nevertheless, our simulated data under scheme III may serve as an validation of the predicted results of SCIPAC. In Figure 2i of the main text, the differentiation steps mimic the different stages of single-cell data. The predicted results of SCIPAC, as depicted in Figure 2j of the main text, indicate that the association strength successfully reflects the stages, capturing both the early and late-stage cells. This result suggests that SCIPAC is likely capable of accurately predicting the stages of cancer cells.

Q3 The interpretation of cell states associated with cancer progression would greatly benefit from the initial comprehensive annotation of clusters. It is not clear why the authors work on not annotated datasets and rely on DE analysis of clusters for the interpretation and vali­dation of the findings. This would be most important for the MKI67+ cluster, which clearly harbors cycling T cells. Here, the cycling signature will obscure cell type gene expression signatures and mix Treg, but also cytotoxic and exhausted T cell into a single cluster. Again, an appropriate fine-grained annotation based on a strong immunological foundation would be most helpful to better evaluate the performance of the algorithm. The same holds true for the lung cancer samples. CD4+ cells are often Treg cells, which should be associated with poorer patient outcome as they relate to a more immunosuppressive tumor microenvi-ronments. Again, a better annotation would be key to interpret the results.

A3 We appreciate the insightful comments provided by the reviewer. We acknowledge the importance of improved annotation for enhancing the biological interpretation of our re­sults. However, it is essential to note that our paper primarily focuses on presenting a methodological study. Consequently, our ability to delve into in-depth biological inter­pretation is limited due to various factors, including time constraints, our expertise and knowledge in different diseases, and the financial resources required for potential wet-lab

experiments. As a result, we opted to adhere to the original cell annotation provided by the publications that generated the data.

When applying SCIPAC to real datasets, we made a deliberate choice to disregard the cell annotation provided by the original publications and instead relied on the inferred cell clusters produced by the Louvain algorithm. We made this decision for several reasons. Firstly, we aimed to ensure a fair comparison with Scissor, as it does not utilize cell-type annotations. Secondly, as the reviewer pointed out, the original annotation might not be sufficiently comprehensive or detailed. Presumed cell types could potentially encompass multiple subtypes, each of which may exhibit distinct associations with the phenotype un­der investigation. In such cases, employing the Louvain algorithm with a relatively high resolution, which is the default setting in SCIPAC, enables us to differentiate between these subtypes and allows SCIPAC to assign varying association strengths to each subtype.

We have incorporated the above discussion concerning the utilization or dismissal of known cell annotations into the Discussion section of the main text. We are grateful to the reviewer for bringing up this important issue.

Q4 Much of the main text can be moved to figure legends. Also, the text can be shortened by moving parts to the Methods, specifically for the simulation section and benchmarking against Scissors.

A4 We appreciate the reviewer’s suggestion. Indeed, implementing these changes would effectively reduce the length of the main text, which is crucial considering that many journals impose strict page limits. It is worth noting, however, that “Method” articles in Genome Biology are published exclusively online and are not subject to page limits. The current style employed in our manuscript aligns with the prevalent format used in such articles, likely due to its enhanced clarity and readability.

**Response to Reviewer 3**

In this manuscript, Gan and colleagues present a computational method called SCIPAC, which associates single-cell transcriptomes with specific phenotypes by integrating bulk and single-cell RNAseq data. The authors demonstrate the method using various types of pheno­types, including binary, ordinal, and continuous. Although SCIPAC shares similarities with a recently developed tool called Scissor, the authors argue that SCIPAC offers two important advantages: estimating the strength and statistical significance of associations for each cell. While the method appears interesting, I have several major concerns.

**Q1** The comparisons between SCIPAC and Scissor are predominantly qualitative, and in most instances, the agreement appears quite limited. This makes it challenging for readers to assess which method provides more meaningful associations. Have the authors tested SCIPAC on the datasets studied in the Scissor paper? Are there any quantitative metrics that can be used for comparison? Currently, the paper relies heavily on UMAPs to compare the methods and demonstrate the consistency of SCIPAC output under various resolution parameters (e.g., the breast cancer dataset). Quantitative measurements are crucial for making the arguments more convincing.

**A1** We appreciate these insightful questions.

The limited agreement between the results from SCIPAC and Scissor can be attributed to two main factors. Firstly, SCIPAC provides quantitative estimations of association strength, whereas Scissor classifies cells into three discrete categories. As a result, the plots of results display different shades of colors in SCIPAC but not in Scissor. Secondly, SCIPAC assigns sim­ilar association strengths to cells with similar expression profiles, resulting in a smoother plot of association strengths. On the other hand, the results of Scissor are composed of scattered red or blue dots. These two reasons contribute to the visual distinction between the results of the two methods. A detailed discussion on this matter can be found in sub­section “Simulation scheme I” of section “Performance in simulated data” and subsection “Prostate cancer data with a binary phenotype” of section “Performance in real data” in our manuscript.

To address the interpretability challenges posed by Scissor’s results, we have suggested considering the “overall impression” of Scissor’s results for each known cell type, specifically by assessing whether red or blue dominates a given region. This approach helps highlight the agreement between the two methods. Further details regarding this recommendation are provided in section “Performance in real data” of our manuscript.

Regarding the real datasets, two out of the four datasets used in our study, namely the

lung cancer data and the muscular dystrophy data, were also utilized in the Scissor paper. The Scissor paper used three real datasets in total; we were unable to access one of these three datasets and included all of the remaining two datasets in our analysis.

For the evaluation of our simulated data, we utilized quantitative metrics, specifically the F1 score and the fraction of sign correctness (FSC). We believe that these measures effectively and efficiently evaluate the performance of the methods. The results, discussed in detail in section “Performance in simulated data” and illustrated in Figures 1f, g and 2f, g, clearly demonstrate the significant and noteworthy outperformance of SCIPAC over Scissor.

In real datasets, comparing methods using quantitative metrics is not feasible since the ground truth is unknown.

**Q2** The association analysis is performed on pseudobulked expression profiles based on either pre-annotated cell type identities or Louvain clusters, rather than directly on single cells. This should be accurately stated in the text. Relatedly, would using metacells (e.g., from SEACells) improve the analysis?

**A2** We appreciate the reviewer for their insightful comments. SCIPAC employs a clustering approach to group cells and utilizes the centroid of each cluster as the representative to compute the association strength using Equation (7) in the main text. The centroid of a cluster is calculated as the mean of all cells within the cluster, resembling a “pseudobulked expression” as suggested by the reviewer. However, the term “pseudobulk” often refers to the mean expression of all cells in a single-cell experiment, which typically consist of multiple cell types. Hence, we do not label SCIPAC as a method based on pseudobulked expression profiles. Furthermore, SCIPAC allows the utilization of other expression profiles, such as the medoid (<https://en.wikipedia.org/wiki/Medoid>), as the representative of a cluster. In such cases, the concept of pseudobulked expression is not applicable.

We would like to thank the reviewer for suggesting the use of metacells [6, 7]. According to our understanding, a metacell shares similarities with a cell cluster, and its representative is analogous to the centroid of a cluster. Therefore, SCIPAC can incorporate metacells and their representatives as an alternative to its default setting of using cell clusters/types and their centroids. As metacells possess advantageous properties, utilizing metacells has the potential to yield better results compared to using SCIPAC with the default setting. We have included a discussion on metacells in the Discussion section, as well as added it to the tutorial of the SCIPAC R package.

**Q3** The calculation of p-values assumes normal distributions of association scores. Is this

assumption supported by experimental data? Additionally, the p-value calculation lacks size correction, so running different numbers of tests at various clustering levels could significantly affect the results.

A3 We appreciate the reviewer’s insightful questions. In order to examine whether the nor­mal assumption holds in real data, we plot histograms of the bootstrapped *Λk*’s for each cluster in our first real dataset, the prostate cancer dataset. These histograms are presented in Fig. RL7. Further, we test whether the bootstrapped *Λk*’s for each cluster are normally distributed using the Shapiro-Wilk’s (S-W) test, and we provide the corresponding p-values in the subplot titles. After multiple-test correction using Benjamini & Hochberg’s false dis­covery rate (FDR) [8], none of the 27 clusters have FDR < 0.05. This suggests that the normal assumption is not significantly violated in this real dataset.

We agree that the p-value calculation lacks size correction—thanks for bringing this up. Applying size correction in our case is challenging due to the strong correlation among the p-values from different clusters. Standard techniques for p-value correction, such as Benjamini & Hochberg’s FDR, do not yield accurate results in this scenario and tend to be overly conservative. Consequently, we made the decision not to apply size correction and instead report the uncorrected p-values. In practice, this approach has not yielded excessively liberal results. For example, in our simulation data 1, even with a high-resolution 3.5 that generates 47 clusters, which corresponds to 47 tests, the uncorrected p-values do not result in a significant number of false positives, as demonstrated in Figure 1d and fin the main text. In summary, we acknowledge that the uncorrected p-value is theoretically imperfect, but based on empirical evidence, it performs adequately and is a preferable choice compared to improperly corrected p-values using methods such as Benjamini & Hochberg’s FDR.

We have included the above discussions on p-values in Supplementary Material. We are grateful to the reviewer for raising this important issue.

Q4 The biological meaning of association strength remains ambiguous. How should we interpret the association strength inferred by SCIPAC for binary phenotypes? For ordinal phenotypes, is there a correlation between differentiation stages and the inferred associations (taking figure 2 as an example)?

A4 We appreciate the reviewer for raising this point, and we apologize for any confusion. Our definition of association is as follows: “a cell is positively/negatively associated with a phenotype if increasing the proportion/fraction of cells that have similar expression profiles as this cell in the tissue will likely indicate an increased/decreased probability for the phe-

notype to be present.” Roughly speaking, by “cells that have similar expression profiles as this cell,” we mean cells of the same cell type. Therefore, in the case of a binary phenotype, a cell type is considered positively/negatively associated with the phenotype if increasing the proportion of this cell type leads to a higher/lower probability of the phenotype occur­ring. The strength of association represents the extent of the increase or decrease in this probability.

Under our definition, if the association strength of cell type A is twice that of cell type B, increasing cell type A by a certain proportion leads to twice the amount of change in the log odds ratio of having the phenotype compared to increasing cell type B by the same proportion.

The association strength under other types of phenotypes can be interpreted similarly, with the major difference lying in the measure of change in probability. For quantitative, ordinal, and survival outcomes, the difference in the quantitative outcome, log odds ratio of the right-tail probability, and log hazard ratio respectively, are used.

Despite the differences in the exact form of the association strength under different types of phenotypes, the underlying concept remains the same: a larger (absolute value of) as­sociation strength indicates that the same increase/decrease in a cell type leads to a larger change in the occurrence of the phenotype.

To clarify this point further, we have added a paragraph in the Discussion section of the main text. We are grateful to the reviewer for pointing out the confusion in the previous submission, which allowed us to provide a clearer explanation in this revised submission.

Regarding the ordinal phenotype shown in Figure 2, we believe there is a correlation between differential stages and the inferred associations. A large absolute value of Λ*k* when the association strength is positive indicates that the cell type *k* is highly associated with later stages. Conversely, a large absolute value of Λ*k* when the association strength is negative indicates that the cell type *k* is highly associated with early stages.

Q5 In the breast cancer dataset, why are cancer-associated fibroblasts assigned a stronger association value than the actual malignant cancer epithelial cells? Is this surprising, and does it raise questions about the value or accuracy of the quantitative association strengths? A5 Thanks to the reviewer for this thoughtful question. We acknowledge that intuitively, malignant cancer epithelial cells (hereafter referred to as “cancer cells”) should be more strongly associated with cancer compared to cancer-associated fibroblasts (CAFs) due to their defining role in cancer progression. However, it is important to note that SCIPAC, like other computation-based methods, is not flawless. In certain datasets, SCIPAC may provide inaccurate imputations of association strength for certain cell types, and there may even be errors in the imputed direction of association.

The observed stronger association between CAFs and the cancer phenotype, compared to cancer cells, could be an instance where SCIPAC inaccurately estimates the strength of the association. Alternatively, this discrepancy could be attributed to the significantly higher in-tertumoral heterogeneity exhibited by cancer cells. It is widely recognized that cancer cells demonstrate substantial intertumoral heterogeneity. In contrast, previous reports indicate that stromal cells such as CAFs predominantly exhibit intratumoral heterogeneity [9]. That is, different prostate cancer patients often exhibit distinct populations of cancer cells, while their CAF populations remain relatively similar. This substantial patient-to-patient variation in cancer cell populations poses challenges in accurately estimating their association with the phenotype, likely leading to an underestimation of this association.

**References**

[1] Xuran Wang, Jihwan Park, Katalin Susztak, Nancy R Zhang, and Mingyao Li. Bulk tissue cell type deconvolution with multi-subject single-cell expression reference. *Nature communications*, 10(1):380, 2019.
[2] Haijing Jin and Zhandong Liu. A benchmark for rna-seq deconvolution analysis under dynamic testing environments. *Genome biology*, 22:1–23, 2021.
[3] Ton N Schumacher and Daniela S Thommen. Tertiary lymphoid structures in cancer. *Science*, 375(6576):eabf9419, 2022.
[4] Wendi Kang, Zhichao Feng, Jianwei Luo, Zhenhu He, Jun Liu, Jianzhen Wu, and Pengfei Rong. Tertiary lymphoid structures in cancer: The double-edged sword role in antitu­mor immunity and potential therapeutic induction strategies. *Frontiers in immunology*, 12:689270, 2021.
[5] Zhan Zhao, Hui Ding, Zheng-bin Lin, Sheng-hui Qiu, Yi-ran Zhang, Yan-guan Guo, Xiao-dong Chu, Loi I Sam, Jing-hua Pan, and Yun-long Pan. Relationship between

tertiary lymphoid structure and the prognosis and clinicopathologic characteristics in solid tumors. *International Journal of Medical Sciences*, 18(11):2327, 2021.

[0] Yael Baran, Akhiad Bercovich, Arnau Sebe-Pedros, Yaniv Lubling, Amir Giladi, Elad Chomsky, Zohar Meir, Michael Hoichman, Aviezer Lifshitz, and Amos Tanay. Meta-cell: analysis of single-cell rna-seq data using k-nn graph partitions. *Genome biology*, 20(1):1–19, 2019.
[1] Sitara Persad, Zi-Ning Choo, Christine Dien, Noor Sohail, Ignas Masilionis, Ronan Chaligné, Tal Nawy, Chrysothemis C Brown, Roshan Sharma, Itsik Pe’er, et al. Seacells infers transcriptional and epigenomic cellular states from single-cell genomics data. *Nature Biotechnology*, pages 1–12, 2023.
[2] Yoav Benjamini and Yosef Hochberg. Controlling the false discovery rate: a practical and powerful approach to multiple testing. *Journal of the Royal statistical society: series B (Methodological)*, 57(1):289–300, 1995.
[3] Lichun Ma, Maria O Hernandez, Yongmei Zhao, Monika Mehta, Bao Tran, Michael Kelly, Zachary Rae, Jonathan M Hernandez, Jeremy L Davis, Sean P Martin, et al. Tumor cell biodiversity drives microenvironmental reprogramming in liver cancer. *Cancer cell*, 36(4):418–430, 2019.

Figure RL4: UMAP visualization of the scRNA-seq prostate cancer data, analyzed by SCIPAC using bulk samples collected from different years, namely parts 1 and 2. (a-b) Association strength and its corresponding p-value given by SCIPAC using bulk samples from part 1. (c-d) Association strength and its corresponding p-value given by SCIPAC using bulk samples from part 2.

Figure RL5: UMAP visualization of the prostate cancer data. (a-c) Association strength given by SCIPAC with the elastic net α = 0.3, 0.4, 0.5, respectively. (d-f) p-values given by SCIPAC with the elastic net α = 0.3, 0.4, 0.5, respectively. These results indicate SCIPAC is robust to the choice of α.

Figure RL6: UMAP visualization of the normal (the first row) and cancer (the second row) prostate data, analyzed by SCIPAC with default settings. By treating the normal samples as the ground truth, it is evident that the association strength and significance are both enchanced.

Figure RL7: Histograms of the bootstrap-generated Λk’s for each cluster in the prostate cancer data. The p-values are calculated using the Shapiro-Wilk’s normality test. The blue lines are the estimated normal curves.

**Second round of review**

**Reviewer 1**

The revised manuscript has addressed most of my questions. One remaining comment is that it would be useful to derive the full likelihood function (page 13), at least for the logistic regression scenario. It’s not clear to me why the computation only depends on \bar{G}, but not individual G and G* vectors.

**Reviewer 2**

I have no more comments.

**Reviewer 3**

In this revision, the authors have made adjustments to the discussion, while leaving the results unchanged. Unfornately, I found the response to be unconvincing for the following reasons.

1, all three reviewers raised concerns regarding the lack of validation and biological meaning in the method, but the revised manuscript offers no additional analysis to address these concerns. The authors stated in their reply: "To address the interpretability challenges posed by Scissor’s results, we have suggested considering the “overall impression” of Scissor’s results for each known cell type, specifically by assessing whether red or blue dominates a given region.". This statement highlights the deficiency in the quantitative analysis of the paper, which appears to be a critical shortcoming.

2, The authors acknowledged in both their reply and the main text that incorporating metacells may enhance the analysis. However, they do not provide any justification for not implementing the analysis, especially considering this improvement would not require significant effort.

3, they conceded that the strong association of fibroblast with cancer is likely a result of "inaccurate imputations of associations strength". If this is indeed an "inaccurate imputation", it raises the questions about the metrics the users can use to evaluate the accuracy of the method. This amplifies the need for rigorous validation of the results, making it a matter of urgent priority.

**Authors Response**

**Point-by-point responses to the reviewers’ comments:**

We extend our gratitude to the reviewers for their valuable comments and to the editor for managing our submission. However, we were profoundly shocked and deeply disap­pointed by the editor’s decision to reject our paper, especially given the positive feedback and acceptance recommendations from Reviewers 1 and 2. We recognize and appreciate the thoroughness of Reviewer 3’s critiques and are committed to addressing these points, hoping for their consideration of our differing viewpoints. In response, we have conducted further analyses and revised our manuscript and Supplementary Material accordingly. These ef­forts are a testament to our commitment to the scientific rigor of our work, representing the limits of our current capabilities. We are hopeful that these comprehensive efforts will address any remaining concerns regarding our paper.

In the text below, the reviewers’ comments are highlighted in blue, and our responses are in standard text.

**Reviewer’s Responses to Questions**

Please provide your comments for the author(s) on the revised manuscript.

Reviewer #1: The revised manuscript has addressed most of my questions. One re­maining comment is that it would be useful to derive the full likelihood function (page 13), at least for the logistic regression scenario. It’s not clear to me why the computation only depends on G^¯^, but not individual G and G^∗^ vectors.

Reviewer #2: I have no more comments.

Reviewer #3: In this revision, the authors have made adjustments to the discussion, while leaving the results unchanged. Unfornately, I found the response to be unconvincing for the following reasons.

1, all three reviewers raised concerns regarding the lack of validation and biological meaning in the method, but the revised manuscript offers no additional analysis to address these concerns. The authors stated in their reply: “To address the interpretability chal­lenges posed by Scissor’s results, we have suggested considering the “overall impression” of Scissor’s results for each known cell type, specifically by assessing whether red or blue dominates a given region.”. This statement highlights the deficiency in the quantitative analysis of the paper, which appears to be a critical shortcoming.

2, The authors acknowledged in both their reply and the main text that incorporating metacells may enhance the analysis. However, they do not provide any justification for not implementing the analysis, especially considering this improvement would not require significant effort.
3, they conceded that the strong association of fibroblast with cancer is likely a result of “inaccurate imputations of associations strength”. If this is indeed an “inaccurate impu­tation”, it raises the questions about the metrics the users can use to evaluate the accuracy of the method. This amplifies the need for rigorous validation of the results, making it a matter of urgent priority.

Evaluation. Has the author satisfactorily responded to your previous review?

Reviewer #1:	Yes

Reviewer #2:	Yes
Reviewer #3: No

**Response to Reviewer 1’s comment**

**Response to Reviewer 3’s Critique 1**

Reviewer 3’s comment: all three reviewers raised concerns regarding the lack of valida­tion and biological meaning in the method, but the revised manuscript offers no additional analysis to address these concerns.

While it is true that “all three reviewers raised concerns regarding the lack of validation and biological meaning in the method,” we cannot agree that “the revised manuscript offers no additional analysis to address these concerns.”

Reviewer 1 posed five major questions (Q1 to Q5). For Q1, which involved confusion about our definition of association, we clarified by explaining our deliberate avoidance of the term “cell type” and revised a relevant sentence in the main text. In response to Q2, which suggested extending our method to consider changes in multiple cell types simul­taneously, we acknowledged the feasibility but pointed out that such an extension might have limited practical utility in real-world applications. Q3 requested a comparison of our approach with a more direct method. We responded by adding theoretical and simulation analyses demonstrating our method’s superiority. Q4 inquired about the impact of associa­tion strength in simulated studies on our method’s performance. We addressed this through additional computational analysis of simulated data. Finally, Q5 questioned the robustness of our method with different bulk datasets, which we answered with further computational analysis using real data.

In summary, we have thoroughly addressed all five questions raised by Reviewer 1. Three of these questions were resolved using supplementary computational analyses, while the remaining two were clarified through detailed theoretical explanations. This comprehensive approach resulted in Reviewer 1 affirmatively responding “Yes” to the evaluation query, indicating their satisfaction with our responses and confirming that there are no longer concerns regarding the validation or biological significance of our method.

Reviewer 2 posed three major questions (Q1 to Q3), all focusing on the biological in­terpretations of our method. The reviewer expressed dissatisfaction with the biological ex­planations in our original manuscript, describing it as “too superficial” in evaluating the SCIPAC’s performance. It was evident from their deep biological insights that this reviewer is highly knowledgeable in biology.

We primarily addressed these concerns through detailed explanations, supplemented by minimal additional computational analysis (as shown in Figure RL6 of our previous re­sponse letter). This approach was chosen because further analysis was either impractical (As we stated in our previous response to Reviewer 2, “We acknowledge the importance of improved annotation for enhancing the biological interpretation of our results. However, it is essential to note that our paper primarily focuses on presenting a methodological study. Consequently, our ability to delve into in-depth biological interpretation is limited due to various factors, including time constraints, our expertise and knowledge in different dis­eases, and the financial resources required for potential wet-lab experiments.” We have also mentioned, “we have collaborated closely with two experts inthe field of cancer, specifically an associate professor and a PhD specializing in molecularbiology and immunotherapy of metastatic cancer. These experts are co-authors of our paperand have actively contributed to the interpretation and evaluation of the SCIPAC results.”) or wouldn’t effectively address the reviewer’s concerns. Our revision turned out to be successful; Reviewer 2 also answered “Yes” to the question, “Has the author satisfactorily responded to your previous review?”

Reviewer 3 posed five questions regarding our original submission. In addressing Ques­tion 3, which queried the normality of the bootstrapped association strength, we supple­mented our response with additional computational analysis. For the remaining questions, we provided detailed and comprehensive explanations. For instance, in response to the critique that our paper “relies heavily on UMAPs to compare methods” and lacks “quantita­tive measurements,” we highlighted our use of multiple quantitative metrics, such as the F1 score and the FSC, in simulated data. We also explained that applying quantitative metrics to real datasets is impractical due to the absence of known ground truth. Additionally, in addressing Question 4’s concern about the “ambiguous biological meaning of association strength,” we provided a detailed and clarifying response.

We regret that Reviewer 3 remains unsatisfied with our revisions, in contrast to the other reviewers who expressed satisfaction. This is particularly notable given that Reviewer 2, who initially had significant concerns about the biological depth of our paper, was content with our response.

Reviewer 3’s comment: The authors stated in their reply: “To address the interpretabil­ity challenges posed by Scissor’s results, we have suggested considering the “overall impres­sion” of Scissor’s results for each known cell type, specifically by assessing whether red or blue dominates a given region.”. This statement highlights the deficiency in the quantitative analysis of the paper, which appears to be a critical shortcoming.

This critique addresses our response to Reviewer 3’s first question about our initial sub­mission. We recognize that describing Scissor’s results based on an “overall impression” may appear vague and less “quantitative.” However, our paper’s quantitative rigor remains robust for several reasons. Firstly, our choice of using “overall impression” is a consequence of Scissor’s limitations, not ours. (We suspect there might be some confusion between the names of our method, SCIPAC, and our competitor’s, Scissor, as both begin with “SCI.” If this is the case, we apologize for any misunderstanding.) The limitations of Scissor complicate a more detailed quantitative comparison. Secondly, despite using an “overall impression” based approach, the conclusions from the comparison remain clear and understandable. Thirdly, in other sections of the paper, particularly in simulated data scenarios where accu­rate answers are known, we have employed multiple quantitative measures for comparison. These comparisons clearly demonstrate the significant superiority of our method. We elab­orate on these three points in the following sections.

Firstly, quantitatively evaluating results from Scissor (our competitor) is challenging. As explained in the “Simulation scheme I” subsection of the “Results” section, this difficulty arises from two main issues: (1) Scissor’s outcomes significantly depend on the selected α value, a parameter that cannot be automatically determined. (2) In some cases, Scissor+ and Scissor- cells appear close to each other in graphical representations, indicating that cells with nearly identical expression profiles are identified as being associated with the phenotype in contrasting directions. These shortcomings in Scissor’s approach make a direct quantitative comparison with our method challenging, especially in the context of real data analysis.

Secondly, even using an “overall impression” based comparison, the conclusion of the comparison between our method and Scissor is sufficiently clear. We focused on the most striking differences between the two methods, those that are visually prominent and consistent across various α values for Scissor. These notable differences are likely the most indisputable and reflective of the distinctions between the methods. Other aspects of the results are less conclusive, as they may show smaller or inconsistent differences depending on Scissor’s α value (a limitation of Scissor, not our method). Including these less definitive aspects in our discussion would unlikely change or strengthen our conclusion. Additionally, some key advantages of our method are apparent even without real-data analysis. These include the ability to provide p-values for associations, easier biological interpretation (as cells with similar expressions typically show similar associations), and simpler tuning (with only one parameter, inherited from Seurat).

Thirdly, quantitative metrics were proposed and used in our analysis of simulated data. As highlighted in our previous response to Reviewer 3’s Question 1, “For the evaluation of our simulated data, we utilized quantitative metrics, specifically the F1 score and the fraction of sign correctness (FSC). We believe that these measures effectively and efficiently evaluate the performance of the methods. The results, discussed in detail in section ‘Performance in simulated data’ and illustrated in Figures 1f, g and 2f, g, clearly demonstrate the signif­icant and noteworthy outperformance of SCIPAC over Scissor. In real datasets, comparing methods using quantitative metrics is not feasible since the ground truth is unknown.”

We hope that our detailed explanation sufficiently persuades Reviewer 3 that our use of “overall impression” in our comparison is an appropriate choice and that our paper main­tains a robust level of quantitativeness. Meanwhile, we understand the reviewer’s con­cern that the term “overall impression” might imply a qualitative approach. To completely eliminate the possibility of this misunderstanding, we will establish a precise mathematical definition of “overall impression” to underscore its quantitative basis, and we will also re­place the phrase “overall impression” to more directly reflect the quantitative nature of our analysis. These efforts demonstrate our commitment to clarity and precision, especially in summarizing Scissor’s results, a task that is inherently complex.

To summarize the inferred association of a known cell type with the phenotype using a specific method (Scissor under a specific α value, or our method SCIPAC with the default setting), we first calculate the proportion of cells in this cell type identified as Scissor+ (by Scissor at a specific α value) or as significantly positively associated (by SCIPAC), denoted by p+. We also calculate the proportion of all cells, encompassing any cell type, which are identified as Scissor+ or significantly positively associated, serving as the average back­ground strength, denoted by p*_a_*. Then, we compute the log odds ratio for this cell type to be positively associated with the phenotype compared to the background, represented as:

Similarly, the log odds ratio for the cell type to be negatively associated with the phenotype, ρ_−_ , is computed in a parallel manner.

For our SCIPAC method, a cell type is summarized as positively associated with the phenotype if ρ+ ≥ 1 and ρ_−_ < 1, and negatively associated if ρ_−_ ≥ 1 and ρ+ < 1. If neither condition is met, the association is inconclusive.

For the competitor method Scissor, we apply it under six different α values: 0.01, 0.05, 0.10, 0.15, 0.20, and 0.25. A cell type is summarized as positively associated with the phenotype if ρ+ ≥ 1 and ρ_−_ < 1 in at least four of these α values, and negatively associated if ρ_−_ ≥ 1 andρ+ < 1 in at least four α values. If these criteria are not met, the association is deemed inconclusive.

The above computation of log odds ratios and the determination of associations are performed only on cell types that each compose at least 1% of the cell population, to ensure adequate power.

For the prostate cancer data, the log odds ratios for each cell type using each method are presented in Tables RL1 and RL2. (Although it is possible to merge these into a single table, such a table would have an excessive number of columns, making it difficult to display. Therefore, we have chosen to present the data in two separate tables.) The final associations determined for each cell type are summarized in Table RL3. In the last column of this table, we also indicate whether the conclusions drawn from SCIPAC and Scissor are consistent or not.

Table RL3 indicates that SCIPAC and Scissor results diverge for three cell types: LE-KLK4, MNP, and B cells. In our previous manuscript, we discussed MNP and B cells based on “overall impression.” This was a justified choice, as these two cell types not only repre­sent the disagreement between the two methods, as indicated in Table RL3, but also are the ones where SCIPAC shows the largest ρ_−_ or ρ+ values (4.2997 and 3.0356, respectively). LE-KLK4, the third cell type, exhibits a smaller ρ_−_ or ρ+ value of 1.349. This quantitative analysis thus corroborates our original “overall impression” conclusion, providing a more mathematically solid basis. Our manuscript has been updated to reflect this quantitative analysis. Additionally, we have incorporated a discussion on LE-KLK4, following this quan­titative analysis. The observed discordance for LE-KLK4 further highlights the effectiveness of our method. LE-KLK4, a subtype of cancer cells, is thought to be positively associated

Table RL1: Log odds ratio for each cell type on the prostate cancer data

SCIPAC	Scissor α = 0.01	Scissor α = 0.05	Scissor α = 0.10

| ρ+ ρ− | ρ+ ρ− | ρ+ ρ− | ρ+ ρ− |
| --- | --- | --- | --- |

| BE | -4.0564 | 1.1675 | -2.6568 | 2.5287 | -2.2179 | 1.9976 | -2.533 | 1.8683 |
| --- | --- | --- | --- | --- | --- | --- | --- | --- |
| HE | -Inf | 2.06 | -4.6335 | 3.4902 | -3.7736 | 2.7619 | -3.3803 | 2.6185 |
| CE | -3.5231 | 0.9539 | -0.5748 | 0.3551 | -0.5334 | 0.2348 | -0.6382 | 0.2162 |
| LE-KLK3 | 0.0479 | 0.1019 | -0.2112 | 0.9292 | 0.0849 | 0.8482 | 0.1039 | 0.7851 |
| LE-KLK4 | 1.349 | -0.3655 | 0.559 | -0.6871 | 0.2528 | -1.0613 | 0.2293 | -1.0984 |
| Endothelial | -Inf | -Inf | -0.4108 | -1.9342 | -0.743 | -1.4059 | -0.6799 | -1.3037 |
| MNP | -Inf | 4.2997 | 3.5965 | -Inf | 2.1398 | -Inf | 1.8219 | -Inf |
| NK cell | -Inf | -Inf | 0.432 | -Inf | 0.2395 | -Inf | 0.2251 | -Inf |
| T cell | -Inf | -4.3215 | -0.4809 | -3.2746 | -0.7756 | -3.2432 | -0.8428 | -3.205 |
| B cell | -Inf | 3.0356 | -0.6692 | -3.3585 | -0.8496 | -Inf | -0.6788 | -Inf |

Table RL2: Log odds ratio for each cell type on the prostate cancer data (Cont’d).

SCIPAC	Scissor α = 0.15	Scissor α = 0.20	Scissor α = 0.25

| ρ+ ρ− | ρ+ ρ− | ρ+ ρ− | ρ+ ρ− |
| --- | --- | --- | --- |

| BE | -4.0564 | 1.1675 | -2.2687 | 1.7723 | -2.0712 | 1.7124 | -1.9079 | 1.6852 |
| --- | --- | --- | --- | --- | --- | --- | --- | --- |
| HE | -Inf | 2.06 | -3.116 | 2.5236 | -2.9185 | 2.5302 | -Inf | 2.544 |
| CE | -3.5231 | 0.9539 | -0.5965 | 0.2776 | -0.5986 | 0.2139 | -0.5366 | 0.0881 |
| LE-KLK3 | 0.0479 | 0.1019 | 0.1432 | 0.7402 | 0.1344 | 0.7258 | 0.1624 | 0.7128 |
| LE-KLK4 | 1.349 | -0.3655 | 0.2123 | -1.0927 | 0.1641 | -1.1057 | 0.0833 | -1.0894 |
| Endothelial | -Inf | -Inf | -0.8167 | -1.1072 | -0.7309 | -1.1489 | -0.6918 | -1.4592 |
| MNP | -Inf | 4.2997 | 1.7557 | -Inf | 1.8078 | -Inf | 1.8524 | -Inf |
| NK cell | -Inf | -Inf | 0.1863 | -Inf | 0.0708 | -Inf | 0.2341 | -Inf |
| T cell | -Inf | -4.3215 | -0.9836 | -3.3463 | -0.9457 | -4.7708 | -0.9881 | -4.6018 |
| B cell | -Inf | 3.0356 | -1.2777 | -Inf | -1.378 | -Inf | -1.6302 | -Inf |

Table RL3: Agreement of the results by SCIPAC and Scissor on the prostate cancer data

|  | SCIPAC | Scissor | Agreement |
| --- | --- | --- | --- |
| BE | Negative | Negative | Agree |
| HE | Negative | Negative | Agree |
| CE | Inconclusive | Inconclusive | Agree |
| LE-KLK3 | Inconclusive | Inconclusive | Agree |
| LE-KLK4 | Positive | Inconclusive | Disagree |
| Endothelial | Inconclusive | Inconclusive | Agree |
| MNP | Negative | Positive | Disagree |
| NK cell | Inconclusive | Inconclusive | Agree |
| T cell | Inconclusive | Inconclusive | Agree |
| B cell | Negative | Inconclusive | Disagree |

with the tumor phenotype [1]. SCIPAC successfully identified this positive association, in contrast to Scissor, which failed to do so.

These newly proposed quantitative measures effectively encapsulate the “overall impres­sion,” particularly for the Scissor method, in the prostate cancer dataset. However, these measures are not necessary in other datasets, since our comparsion with Scissor does not de­pend on the “overall impression” on these datasets: Scissor’s results are not included in the cancer dataset as it does not work on ordinal phenotypes; the muscular dystrophy dataset lacks cell type labels, and our current manuscript’s comparison does not involve “overall impression”; and the lung cancer dataset does not feature a direct comparison with Scissor, as Scissor’s results are too sparse and scattered.

In conclusion, while we still maintain that our initial submission was not lacking in quantitativeness, our new quantitative findings have solidified our “overall impression” and enhanced our analysis, effectively precluding any potential misinterpretation of this term. We are grateful to the reviewer for highlighting this aspect.

Response to Reviewer 3’s Critique 2

Reviewer 3’s comment: The authors acknowledged in both their reply and the main text that incorporating metacells may enhance the analysis. However, they do not provide any justification for not implementing the analysis, especially considering this improvement would not require significant effort.

The reviewer is right in noting that, although we recognized their suggestion to use metacells, we did not implement it. Instead, we indicated that exploring this would be part of our future work. This decision was based on two primary reasons.

First, we view the use of metacells as an enhancement to our method, not a fundamen­tal requirement. Successfully applying our method to metacells would broaden its appli­cability, but we maintain that the method is already highly valuable in its current form. Metacells, while having the potential to be widely adopted, have not yet become the main­stream method for storing and presenting single-cell expression data. Most single-cell data are stored and utilized at the individual cell level. Therefore, we believe it is unreasonable to reject our paper for not including what we consider an extension, rather than a crucial component of the original design.

Second, contrary to the assumption that integrating the analysis “would not require sig­nificant effort,” implementing the SEACells Python package, as cited in [2] and available on its GitHub repository (<https://github.com/dpeerlab/SEACells>), proved challenging. Despite following all recommended installation procedures, we encountered persistent errors. Further investigation revealed conflicts among the required package versions, making a suc­cessful installation of SEACells unfeasible. This issue is not unique to us; similar installation difficulties have been reported by others on the package’s GitHub issues page.

For these two reasons, we initially did not implement SEACells. We maintain that even with a smooth installation process, integrating SEACells with our method represents an extension beyond its original scope. We believe deferring this integration to future work is a reasonable decision.

However, in light of the reviewer’s insistence, we understand that we may need to pro­ceed with the implementation despite our reservations. Consequently, we downloaded SEA-Cells’ source code and utilized only its core function, *SEACells*, from the file [*code.py*](http://code.py). After navigating and resolving package conflicts by identifying compatible package versions, we were ultimately able to conduct our analysis using SEACells.

Metacells are distinct and homogeneous groups of cells [2, 3, 4, 5]. They serve to sim­plify large single-cell datasets by representing the original data with a reduced number of metacells. We detail below how we integrate metacells into our SCIPAC algorithm.

As outlined in the “Results” section of our main text, SCIPAC involves four steps. The initial step uses the Louvain algorithm from the Seurat package to cluster cells and employs cluster centroids as their representatives. These centroids are then inputted into the subse­quent steps (2 to 4) of SCIPAC for computing association strength and assessing statistical significance.

For incorporating metacells into SCIPAC, we propose two approaches. The first approach treats each metacell as a representative of a cell cluster, essentially replacing SCIPAC’s first step with SEACells. Hence, metacells become the input for SCIPAC’s steps 2 to 4. The second approach considers metacells as a condensed single-cell dataset, which is then clustered using SCIPAC’s Louvain algorithm. Here, metacells are inputted into the entire SCIPAC process, from steps 1 to 4.

**Results from the first strategy**

In our study, we consider Simulation dataset 1, which comprises three cell types: one pos­itively associated, one negatively associated, and one not associated. We used SEACells on this dataset to create three metacells. These metacells are treated as representatives of their corresponding cell groups and are integrated into steps 2 to 4 of the SCIPAC process. The results are shown in Figure RL1.

Figure RL1: Results of SCIPAC on three metacells. Both plots are scatterplots of the two dimensional single-cell data given by UMAP. Points in the plots represents single cells, and they are colored differently in each subplot to reflect different information/results. (a) As­sociation strengths A. Red/blue represents the sign of A, and the shade gives the absolute value of A. Every cell is colored red or blue since no A is exactly zero. (b) p-values. Only cells with p-value < 0.05 are colored red (positive association) or blue (negative associa­tion); others are colored white.

Surprisingly, no cells were identified as significantly associated with the phenotype. This raises the question: Why did the SEACells+SCIPAC combination fail? To investigate, we examined how the metacells grouped cells.

Figure RL2: UMAP plot of single cells, color-coded based on their assignments into three metacells as determined by SEACells.

Figure RL2 displays cells categorized into distinct metacells, each represented by a unique color. The centroids of these metacells are labeled as 1, 2, and 3. Metacells 1 and 3 predominantly consist of the null cell type, whereas metacell 2 is a composite of the posi­tively associated, negatively associated, and a fraction of the null cell types. Consequently, none of these metacells should be linked to the phenotype, corroborating the accurate in­ference by our SCIPAC algorithm. The ineffectiveness of the SEACells+SCIPAC combination is attributed to SEACells’ erroneous cell grouping.

We can quantitatively measure the agreement of cell assignments (using either Louvain clustering or metacells) to the true cell labels using the Adjusted Rand Index (ARI), which is a commonly used measure of similarity between two data clusterings. ARI ranges from -1 to 1, where 1 indicates perfect agreement, 0 no agreement, and -1 complete disparity. The ARI for Louvain clustering compared to the true clustering is 0.9951, while for metacells it is only 0.0320.

Could increasing the number of metacells enhance the SEACells+SCIPAC combination’s effectiveness? Possibly, as a greater number of metacells increases the chances of positively and negatively associated cells being segregated into different metacells, decreasing their mixture with other cell types.

Initially, we raised the number of metacells to five. The outcomes are depicted in Figure RL3, and the cell distribution by metacells is illustrated in Figure RL4. This led to successful

identification of many positively associated cells, primarily because metacell 5, to which they are assigned, contains few cells of other types. Conversely, negatively associated cells remain unidentified. They are mainly grouped into metacell 3, which has a significant number of null cells and a substantial fraction of positively associated cells. The centroid of metacell 3 aligns more closely with the null cell type than the negatively associated cell type, suggesting its overall association is nearly null. Thus, the subpar performance of the SEACells+SCIPAC combination can still be ascribed to the inefficiencies of SEACells.

Figure RL3: Results of SCIPAC on five metacells. Both plots are scatterplots of the two di­mensional single-cell data given by UMAP. Points in the plots represents single cells, and they are colored differently in each subplot to reflect different information/results. (a) As­sociation strengths A. Red/blue represents the sign of A, and the shade gives the absolute value of A. Every cell is colored red or blue since no A is exactly zero. (b) p-values. Only cells with p-value < 0.05 are colored red (positive association) or blue (negative associa­tion); others are colored white.

Figure RL4: UMAP plot of single cells, color-coded based on their assignments into five metacells as determined by SEACells.

Subsequently, we expanded the number of metacells to 20. The results of this increase are displayed in Figure RL5, with the cell grouping within these metacells detailed in Figure RL6. Following this adjustment, a small fraction of negatively associated cells is now accu­rately identified. This improvement is attributed to their assignment to metacell 4, which contains few cells from other cell types. However, the majority of negatively associated cells continue to be unrecognized. This is because they are predominantly grouped into metacells that include a high proportion of cells from other types.

Figure RL5: Results of SCIPAC on 20 metacells. Both plots are scatterplots of the two dimen­sional single-cell data given by UMAP. Points in the plots represents single cells, and they are colored differently in each subplot to reflect different information/results. (a) Association strengths A. Red/blue represents the sign of A, and the shade gives the absolute value of A. Every cell is colored red or blue since no A is exactly zero. (b) p-values. Only cells with p-value < 0.05 are colored red (positive association) or blue (negative association); others are colored white.

Figure RL6: UMAP plot of single cells, color-coded based on their assignments into 20 meta-cells as determined by SEACells.

Unlike SEACells/metacells, which struggles with effectively grouping cells, the Louvain algorithm employed by SCIPAC demonstrates superior performance in this task. Figure RL7

shows how the Louvain algorithm clustered the cells into 3, 5, and 20 clusters. The corre­sponding centroids are also shown. In the 3-cluster result, each cluster closely matched a cell type. In the 5-cluster result, three larger clusters (clusters 1, 2, and 3) closely aligned with the original three cell types, while the remaining two clusters contained a small num­ber of cells. In the 20-cluster results, we found that the cell clusters corresponding to the negatively/positively associated cells typically did not contain cells from the other two types.

Figure RL7: UMAP plot of single cells, color-coded based on their assignments into different clusters as determined by the Louvain algorithm used by SCIPAC. (a) 3 clusters. (b) 5 clusters. (c) 20 clusters.

Finally, we checked whether the reason for the failure of the SEACells+SCIPAC combination— the metacells being inferior clusters of cells—applies to real data. Figure RL8 shows cells colored according to true cell types (subfigure a, 13 cell types), cells colored according to metacells given by SEACells (subfigure b, 13 metacells), and cells colored according to clusters given by the Louvain algorithm (subfigure, 13 clusters). The centroids are also illustrated.

Figure RL8: UMAP plot of single cells, color-coded based on their true cell types or their assignments into different clusters/metacells. **(a)** 13 true cell types. **(b)** 13 metacells given by SEACells. **(c)** 13 clusters as determined by the Louvain algorithm used by SCIPAC.

It is clear that the clusters given by the Louvain algorithm are closer to the true cell types than those given by SEACells, which show much more mixing. Again, we used ARI to quantitatively measure the agreements. The ARI between the cell clusters given by the Louvain algorithm is 0.4914, while the ARI for the metacell-based clusters is only 0.2883.

**Results from the second strategy**

In this strategy, we use metacells to reduce the size of the original single-cell data, and then use these metacells as input for the SCIPAC algorithm. The default parameter in SEACells generates 130 metacells for our simulation dataset 1, which consists of 10,000 cells. These metacells are further clustered by the Louvain algorithm in the first step of SCIPAC, and the number of metacell clusters depends on the resolution (“Res”) parameter in the Louvain algorithm.

Figure RL9 presents the ground truth of how these metacells are associated with the phenotype. In the plot, each metacell is colored according to the majority cell type of the cells it contains. This majority cell type also reflects how each metacell should be associated with the phenotype, as the three cell types have different associations with the phenotype: one positive, one negative, and one neutral. Out of the 130 metacells, 22 predominantly contain cells that are either positively or negatively associated with the phenotype. All of these metacells are located in the top-right corner of the plot.

Figure RL9: UMAP plot of metacells, color-coded according to the majority cell type of cells in the metacell.

In Figure RL10a and Figure RL11a, the results for estimated association strengths and p-values are shown, respectively, at a resolution of 1.0, which groups the 130 metacells into two clusters. At this resolution, no cells are identified as significantly associated.

Figures RL10b & c and RL11b & c show the outcomes at Res = 1.1 and 1.2, yielding four and seven clusters, respectively. However, these settings also fail to yield any significant findings.

Increasing the resolution further to 1.4, we observe a substantial increase in clusters, totaling 21, as shown in Figure RL10d and Figure RL11d. Despite this increase, the results still do not reveal any significant associations.

Figure RL10: The estimated association strength given by SCIPAC under different resultions. Points in each plot represents metacells, and they are colored accoding to the association strengths Λ. (a) Resolution 1.0, which gives 2 clusters. (b) Resolution 1.1, which gives 4 clusters. (c) Resolution 1.2, which gives 7 clusters. (d) Resolution 1.4, which gives 21 clusters.

Figure RL11: The p-values given by SCIPAC under different resultions. Points in each plot represents metacells, and they are colored accoding to the p-values. Only cells with p-value < 0.05 are colored red (positive association) or blue (negative association); others are colored white. (a) Resolution 1.0, which gives 2 clusters. (b) Resolution 1.1, which gives 4 clusters. (c) Resolution 1.2, which gives 7 clusters. (d) Resolution 1.4, which gives 21 clusters.

Do these poor results still stem from the inadequate performance of metacells in group­ing cells? To explore this, we examine how the Louvain algorithm groups these 130 meta-cells into clusters at different resolutions, as shown in Figure RL12.

Figure RL12: UMAP plot of metacells, color-coded based on their assignments into different numbers of clusters given by the Louvain algorithm. The centroids of the clusters are also highlighted. (a) Resolution 1.0, which gives 2 clusters. (b) Resolution 1.1, which gives 4 clusters. (c) Resolution 1.2, which gives 7 clusters. (d) Resolution 1.4, which gives 21 clusters.

Remarkably, in all scenarios, including at a resolution of 1.4 with 21 clusters, all of the 22 metacells that mainly contain cells (either positively or negatively) associated with the phenotype, which are located in the top-right corner of each subplot, consistently fall into a single cluster. This cluster, because it encompasses all of the 22 metacells, contains a nearly equal mix of positively and negatively associated cells. Therefore, this cluster should not be linked to the phenotype. SCIPAC’s analysis supports this conclusion, correctly inferring the non-association of this cluster with the phenotype.

We may delve further and question why the Louvain algorithm consistently groups all these 22 metacells into a single cluster. This is particularly puzzling given that the Louvain algorithm effectively clustered the original single cells, separating positively and negatively

associated cells into different clusters even when *K* = 3, as detailed in our manuscript. Is this due to those metacells each containing both positively and negatively associated cells, making these metacells not very distinct from each other? Upon examining the cell type labels within each metacell, we confirm this to be the case. Among the 22 metacells where the majority of cells are associated with the phenotype, only three (13.6%) are not a mix of both positively and negatively associated cells. In 11 of these 22 metacells (50.0%), the mix ratio is at least 10:90. In 4 of these 22 metacells (18.2%), the mix ratio is at least 40:60. Clearly, metacells performed poorly in grouping cells according to their cell type, and thus, by extension, their function.

**Quick summary**

Our comparative analysis reveals that combining SCIPAC with SEACells results in significantly reduced performance compared to using SCIPAC directly on original single-cell data. The primary reason for this appears to be the subpar performance of SEACells/metacells in cell grouping, especially when contrasted with the Louvain algorithm. This issue is attributed to SEACells/metacells, not to any inherent problem with our SCIPAC algorithm. Given these findings, we currently do not suggest using SEACells/metacells for SCIPAC applications. We have revised our manuscript to reflect these insights, and have added our comprehensive analysis, as discussed above, to the Supplementary Material.

On a positive note, SCIPAC, along with the embedded Louvain algorithm, demonstrates sufficient computational efficiency and manageable memory usage even with large single-cell datasets. This eliminates the need for metacells to reduce data size. To demonstrate this, we conducted an experiment using the prostate cancer dataset, artificially expanding the sample size from 8,657 to 200,000 cells. This was achieved by oversampling pre-processed data with replacement and adding random Gaussian noise. SCIPAC was then run on this data using a MacBook Pro with an Apple M1 chip, 8 cores, and 16GB RAM. The initial clustering step using the Louvain algorithm took approximately 46 minutes, while the sub­sequent SCIPAC processes were completed in under two minutes. The peak memory usage was only 2.5 GB.

**Response to Reviewer 3’s Critique 3**

Reviewer 3’s comment: they conceded that the strong association of fibroblast with cancer is likely a result of “inaccurate imputations of associations strength”.

In our response letter, we proposed two potential explanations for the strong association of fibroblasts with cancer. First, it “could be an instance where SCIPAC inaccurately esti­mates the strength of the association.” Alternatively, “this discrepancy could be attributed to the significantly higher intertumoral heterogeneity exhibited by cancer cells.” We provided reasons and supporting literature for the latter possibility. Our stance was not a “conces­sion”; rather, we acknowledged the former possibility as part of a broader context, as we noted, “it is important to note that SCIPAC, like other computation-based methods, is not flawless.” From our perspective, if we cannot be certain that this is not an error (although we did provide literature supporting the possibility that it might not be an error, as cited in the latter explanation), we should not dismiss the possibility of an error. However, it appears that our scientifically rigorous approach and stance have been misinterpreted as a concession.

Reviewer 3’s comment: If this is indeed an “inaccurate imputation”, it raises the ques­tions about the metrics the users can use to evaluate the accuracy of the method. This amplifies the need for rigorous validation of the results, making it a matter of urgent prior­ity.

We cannot agree with the rationale provided by the reviewer for this justification. In our view, even if our method does result in an “inaccurate imputation” (which, we emphasize, is not an admission of error), our paper should not be rejected on that basis alone.

No method is perfect. It is unrealistic to expect any computational method to align 100% with experimental data or established biological knowledge. It is similarly unreasonable to demand complete accuracy from experimental methods; even the gold standard of RNA abundance measurement, qRT-PCR, may have inaccuracies.

The acceptance of a new computational method should instead be based on whether it offers overall advantages compared to existing methods. In our case, we firmly believe this to be true: we have highlighted significant advantages of our method over the existing Scissor method in the abstract, introduction, and discussion. We have demonstrated these advantages through both simulation data and real data. Notably, on this breast cancer dataset with an ordinal phenotype, the existing method, Scissor, is not even applicable.

Furthermore, we would like to reference Question 1 from Reviewer 2 in our previous submission. Similar to Reviewer 3, Reviewer 2 expressed doubts about a particular associa­tion we inferred: “If B cells infiltration and the formation of TLS is cancer-specific, shouldn’t B cells be positively associated with cancer as predicted by Scissor? To my understanding, a positive association signals cancer-specificity.” We addressed this concern in two ways: first, by explaining why cancer-specificity does not necessarily imply a positive association from a theoretical standpoint; and second, by citing literature that suggests the possibility of a positive association. It is important to note that we did not dismiss, nor did we attempt to dismiss, the possibility of an error on our part. Reviewer 2 did not further challenge us on this point, nor did they reject our paper because of it. Given the complexity of biology, the noise inherent in real data, and the unknown nature of the true answers in real data, expecting perfection may be overly stringent.

**References**

[1] Zewen Kelvin Tuong, Kevin W Loudon, Brendan Berry, Nathan Richoz, Julia Jones, Xiao Tan, Quan Nguyen, Anne George, Satoshi Hori, Sarah Field, et al. Resolving the immune landscape of human prostate at a single-cell level in health and cancer. *Cell Reports*, 37(12), 2021.
[2] Sitara Persad, Zi-Ning Choo, Christine Dien, Noor Sohail, Ignas Masilionis, Ronan Chaligné, Tal Nawy, Chrysothemis C Brown, Roshan Sharma, Itsik Pe’er, et al. Seacells infers transcriptional and epigenomic cellular states from single-cell genomics data. *Nature Biotechnology*, pages 1–12, 2023.
[3] Yael Baran, Akhiad Bercovich, Arnau Sebe-Pedros, Yaniv Lubling, Amir Giladi, Elad Chomsky, Zohar Meir, Michael Hoichman, Aviezer Lifshitz, and Amos Tanay. Meta-cell: analysis of single-cell rna-seq data using k-nn graph partitions. *Genome biology*, 20(1):1–19, 2019.
[4] Oren Ben-Kiki, Akhiad Bercovich, Aviezer Lifshitz, and Amos Tanay. Metacell-2: a divide-and-conquer metacell algorithm for scalable scrna-seq analysis. *Genome biology*, 23(1):1–18, 2022.
[5] Mariia Bilous, Loc Tran, Chiara Cianciaruso, Aurélie Gabriel, Hugo Michel, Santiago J Carmona, Mikael J Pittet, and David Gfeller. Metacells untangle large and complex single-cell transcriptome networks. *BMC bioinformatics*, 23(1):336, 2022.

**Third round of review**

**Reviewer 1**

I think the manuscript is ready for publication.
